# Adverse event profiles of immune checkpoint inhibitor-based strategies for metastatic prostate cancer: a systematic review and network meta-analysis

**DOI:** 10.3389/fmed.2026.1904711

**Published:** 2026-10-30

**Authors:** Qingyuan Wang, Chuang Hao, Jun Chen, Wenzhi Wang, Hao Zhang

**Affiliations:** Urology Surgery, The Second Affiliated Hospital of Zhejiang Chinese Medical University, Hangzhou, China

**Keywords:** adverse events, combination therapy, immune checkpoint inhibitors, metastatic prostate cancer, network meta-analysis

## Abstract

**Background:**

Immune checkpoint inhibitor (ICI)-based combination strategies are increasingly evaluated in metastatic prostate cancer (mPCa), but their comparative safety profiles remain uncertain. We conducted a systematic review and network meta-analysis (NMA) to compare adverse event (AE) profiles of ICI-based strategies in mPCa.

**Methods:**

PubMed, Embase, Cochrane Library, and Web of Science were searched. Ten randomized trials involving 8,197 participants and seven predefined treatment nodes were analyzed. Outcomes included any-grade AEs, grade ≥3 AEs, serious AEs, fatal AEs, any-cause AEs, and selected organ-specific immune-related AEs (diarrhea, rash, hypothyroidism, fatigue). Random-effects NMA estimated odds ratios (ORs) with 95% confidence intervals (CIs). SUCRA values were considered exploratory only. Transitivity was assessed qualitatively, and sensitivity analysis excluded metastatic hormone-sensitive prostate cancer (mHSPC) trials. Certainty was summarized using a structured qualitative assessment.

**Results:**

All outcome networks were star-shaped, with no closed loops; therefore, loop inconsistency could not be formally assessed. In the endocrine-therapy subgroup, adding an ICI to an ARPI-based endocrine backbone significantly increased any-grade AEs (OR 1.33, 95% CI 1.19–1.47), grade ≥3 AEs (OR 2.85, 2.36–3.45), serious AEs (OR 5.33, 3.89–7.31), and fatal AEs (OR 8.03, 1.84–35.02), but not any-cause AEs (OR 1.04, 0.92–1.18). Across the NMA, CTLA-4-containing regimens and PD-(L)1 plus endocrine therapy showed less favorable signals for serious and fatal AEs, whereas PD-1 plus chemotherapy ranked relatively favorably in exploratory SUCRA analyses for several outcomes. However, fatal-event estimates were based on few events, several CIs were wide, and sensitivity analysis excluding mHSPC trials changed the precision and, for some comparisons, the direction or statistical significance of estimates.

**Conclusions:**

In this exploratory NMA, ICI-based combination strategies in mPCa showed heterogeneous safety profiles. Adding ICIs to endocrine therapy was associated with increased AEs. PD-1 plus chemotherapy ranked relatively favorably in exploratory SUCRA analyses, but this should not be interpreted as confirmed safety superiority, and no regimen should be regarded as definitively safest. Certainty was limited by sparse, predominantly star-shaped networks, indirect comparisons, variable AE definitions, and imprecise estimates. Head-to-head randomized trials with standardized safety reporting are needed.

**Systematic Review Registration:**

PROSPERO identifier CRD420261342375.

## 1 Introduction

Prostate cancer (PC) remains the second most commonly diagnosed malignancy and the fifth leading cause of cancer-related mortality among men worldwide (1). The management of metastatic prostate cancer (mPCa) continues to pose a major clinical challenge. Epidemiological modeling by the US National Cancer Institute (NCI) estimated that approximately 120,400 men in the United States were living with metastatic prostate cancer in 2018; among them, 45% presented with *de novo* metastatic disease and 55% developed metastatic recurrence. This population is projected to increase to 192,500 by 2030, with marked racial disparities; the prevalence among Black men is substantially higher than that among White men, at 137.1 vs. 62.2 per 100,000, respectively (2). Progression to metastatic disease is not limited to patients who initially present with metastases. Some patients with localized prostate cancer may develop distant metastases during follow-up, even after receiving definitive treatment. Specifically, approximately 10%–20% of patients with high-risk localized prostate cancer progress to mPCa within 5 years of diagnosis (3, 4). Once metastasis occurs, patients frequently experience severe complications associated with bone metastases, including severe bone pain, pathological fractures, and spinal cord compression. These complications, together with fatigue and anemia, substantially impair quality of life and increase healthcare burden (5). Taxane-based chemotherapy and novel androgen receptor pathway inhibitors have been widely used across different stages of mPCa, and targeted radiopharmaceuticals have also demonstrated efficacy in advanced disease. Nevertheless, preventing disease progression and treating established mPCa remain challenging despite these therapeutic advances. In the United States, the overall incidence of mPCa increased between 2009 and 2013, with a persistent rise among men aged 45–64 years; moreover, the long-term prognosis remains poor (6, 7).

The advent of immune checkpoint inhibitors, particularly those targeting programmed cell death protein 1 (PD-1) and cytotoxic T-lymphocyte-associated protein 4 (CTLA-4), has transformed the treatment landscape for multiple solid tumors (8). PD-1 and CTLA-4 inhibitors have demonstrated clinical efficacy in melanoma, non-small cell lung cancer, triple-negative breast cancer, and mismatch repair-deficient colorectal cancer (9, 10). However, prostate cancer is generally regarded as an immunologically cold tumor, characterized by a low tumor mutational burden and limited infiltration of tumor-infiltrating lymphocytes (TILs). These features contribute to the limited efficacy of immune monotherapy (11). To overcome this immunosuppressive microenvironment, recent clinical investigations have increasingly focused on combination strategies. In these approaches, immune checkpoint inhibitors (ICIs) are combined with agents or modalities that induce immunogenic cell death or modulate the tumor microenvironment, including dual immunotherapy (PD-1 plus CTLA-4 blockade), vaccines, radiotherapy, and targeted or hormonal therapies, with the aim of synergistically enhancing antitumor efficacy (12). Several trials have reported encouraging activity of ICI-based combinations in selected mPCa populations. For example, in the CheckMate 650 trial, nivolumab plus ipilimumab achieved an objective response rate of 25% in chemotherapy-naive patients with metastatic castration-resistant prostate cancer (mCRPC), compared with 10% in post-chemotherapy patients. Four complete responses were observed, and the median overall survival was 19.0 and 15.2 months in the two cohorts, respectively (13). In the KEYNOTE-046 trial, the combination of a vaccine and a PD-1 inhibitor was associated with a median overall survival of 33.7 months (14). Similarly, cabozantinib combined with atezolizumab improved progression-free survival in the CONTACT-02 trial, with a median progression-free survival of 6.3 vs. 4.2 months (HR, 0.65; *P* = 0.0007) (15). These findings suggest that combination strategies may provide clinical benefit in selected patients with mPCa.

Although combination regimens may improve efficacy, they may also increase the risk of adverse events. If not promptly recognized and managed, immune-related toxicities may lead to treatment interruption, irreversible organ damage, or fatal outcomes (16–18). This safety concern is particularly relevant to patients with mPCa, who are predominantly older men with multiple comorbidities and limited physiological reserve (19, 20). Although several RCTs have compared individual ICI-containing regimens with conventional therapy, direct head-to-head comparisons among different ICI-based combination strategies remain scarce. To address this evidence gap, we conducted a systematic review and network meta-analysis. Compared with conventional direct comparisons or pairwise meta-analyses, network meta-analysis (NMA) integrates both direct and indirect evidence. This approach enables simultaneous comparison of the safety of multiple treatment strategies in the absence of head-to-head trials and allows exploratory ranking of adverse-event risks. The findings may provide comparative evidence on adverse-event risks and serve as an important reference for precise risk assessment and individualized treatment decision-making in clinical practice.

## 2 Methods

### 2.1 Study design and registration

This study was designed and reported in accordance with the Preferred Reporting Items for Systematic Reviews and Meta-Analyses (PRISMA) 2020 statement and was registered with PROSPERO (CRD420261342375).

### 2.2 Inclusion and exclusion criteria

The inclusion criteria were defined according to the PICOS framework: (1) Participants: male patients with clinically confirmed metastatic prostate cancer (mPCa); (2) Interventions: immune checkpoint inhibitors (ICIs) administered as monotherapy or in combination regimens; (3) Comparators: conventional therapy; (4) Outcomes: adverse events of any grade, grade ≥3 adverse events, serious adverse events, fatal adverse events, any-cause adverse events, and Specific adverse events (including diarrhea, rash, hypothyroidism and fatigue); and (5) Study design: eligible randomized controlled trials (RCTs).

The exclusion criteria were as follows: (1) non-English-language publications; (2) studies for which the full text was unavailable or from which insufficient data could be extracted; (3) non-original studies, including animal studies, meta-analyses, systematic reviews, conference abstracts, letters to the editor, editorial replies, clinical guidelines, and case reports; and (4) studies that did not include immune checkpoint inhibitor (ICI) interventions.

### 2.3 Search strategy

A comprehensive literature search was conducted to identify relevant randomized controlled trials (RCTs) investigating immune checkpoint inhibitors in prostate cancer. PubMed, Embase, the Cochrane Library, and Web of Science were searched from database inception to September 17, 2026. A systematic search strategy was developed by combining MeSH terms and free-text terms related to “prostate neoplasms,” “immune checkpoint inhibitors,” and specific drug names. The strategy was adapted as appropriate to the indexing system of each database. In addition, the reference lists of relevant systematic reviews and meta-analyses were manually screened to identify additional eligible studies. The detailed search strategy is provided in Supplementary Appendix S1.

### 2.4 Quality assessment

Two investigators independently assessed the risk of bias in the included RCTs using version 2 of the Cochrane Risk of Bias tool (RoB 2), which evaluates five domains: the randomization process, deviations from intended interventions, missing outcome data, outcome measurement, and selection of the reported result. Each domain was rated as having low risk, some concerns, or high risk. Any disagreements were resolved through discussion or adjudication by a third reviewer.

### 2.5 Statistical analysis

Network meta-analysis was performed using Stata version 16.0. A random-effects consistency model was fitted using the Stata network meta-analysis routine. Between-study heterogeneity was modeled using a common network-wide between-study variance (τ^2^), which was estimated for each outcome. Because the networks were sparse, *I*^2^ was not reliably estimable/reportable and was therefore not used as the primary heterogeneity metric, τ^2^ and qualitative assessment were used instead. Ranking probabilities for each treatment strategy and the surface under the cumulative ranking curve (SUCRA) were calculated using the network rank command with Monte Carlo simulations. These analyses estimated the probability that each regimen would occupy a more favorable safety position for each outcome. For dichotomous outcomes, effect estimates were expressed as odds ratios (ORs) with corresponding 95% confidence intervals (CIs). CI limits below 0.01 were reported as <0.01. Probability-based treatment rankings were used only as supplementary quantitative analyses and should not be directly interpreted as evidence of clinical superiority or safety. Comparison-adjusted funnel plots were used to assess small-study effects when a sufficient number of studies was available.

## 3 Results

### 3.1 Study selection

A total of 3,889 records were initially identified through the literature search. After duplicate records were removed, titles and abstracts were screened, and potentially eligible articles were retrieved for full-text assessment. Ultimately, 10 studies met the inclusion criteria and were included in the network meta-analysis (Figure 1) (15, 21–29).

**Figure 1:**
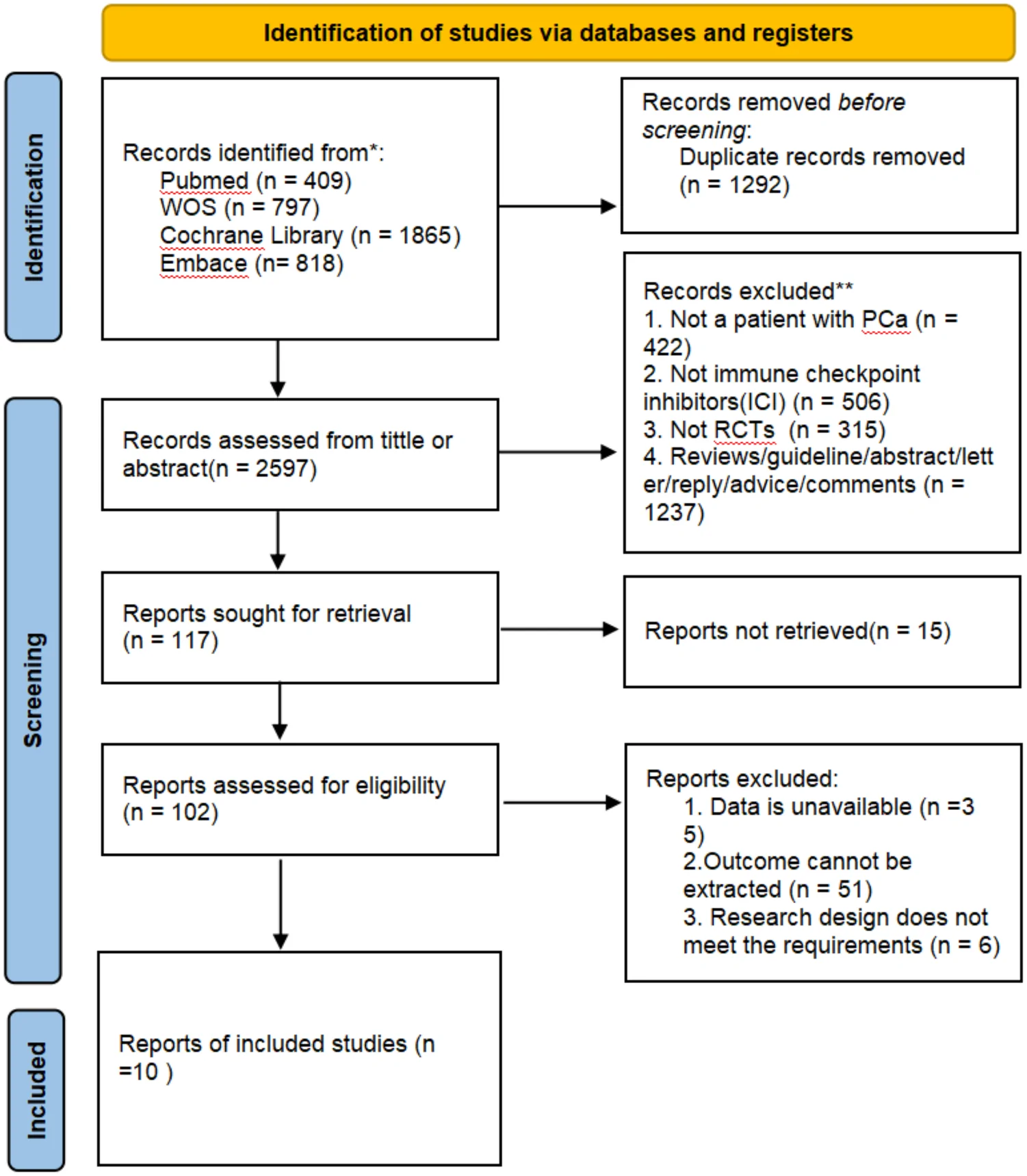
PRISMA flow diagram of study identification, screening, eligibility assessment, and inclusion. Ten randomized controlled trials were included in the systematic review and network meta-analysis.

### 3.2 Characteristics of the included studies

The 10 included studies enrolled a total of 8,197 participants and evaluated 7 different treatment interventions. These regimens included conventional therapy, ICI monotherapy with CTLA-4 inhibitors, and ICI-based combination strategies involving endocrine therapy, chemotherapy, targeted therapy, or radiotherapy. Treatments grouped within the same node were considered comparable at the level of drug class and background therapy. The conventional therapy node included different control regimens, which may have different baseline toxicity profiles; this was considered a potential source of heterogeneity and is discussed in the Limitations. Serious and fatal adverse events were not mutually exclusive categories; serious adverse events often include death as a subcategory. These outcomes were extracted as reported in the original studies. Detailed baseline characteristics, node definitions, specific regimens, background therapies, disease settings, adverse-event definitions, grading systems, attribution, safety populations, follow-up durations, and event counts per arm are summarized in Supplementary Tables S1-1–S1-5.

### 3.3 Quality assessment

The methodological quality of the 10 included RCTs was assessed using version 2 of the Cochrane Risk of Bias tool (RoB 2). For the randomization process domain, two studies were judged as low risk and eight as having some concerns. With respect to bias arising from deviations from intended interventions, nine studies were assessed as having low risk, and one was assessed as having some concerns. All 10 studies were considered to have a low risk of bias due to missing outcome data. Outcome measurement was judged to have a low risk of bias in five studies, whereas the remaining five studies were rated as having some concerns. In addition, one study was considered to have a low risk of bias in the selection of the reported result, whereas nine studies were rated as having some concerns. The risk-of-bias assessment results for the 10 included RCTs are presented in Figure 2 and Supplementary Figure S1. The included trials were judged to have at least some concerns in several risk-of-bias domains, and a structured qualitative certainty assessment indicated low certainty for all outcomes because of risk of bias, indirectness, imprecision, heterogeneity, and network sparsity in Supplementary Table S1-6.

**Figure 2:**
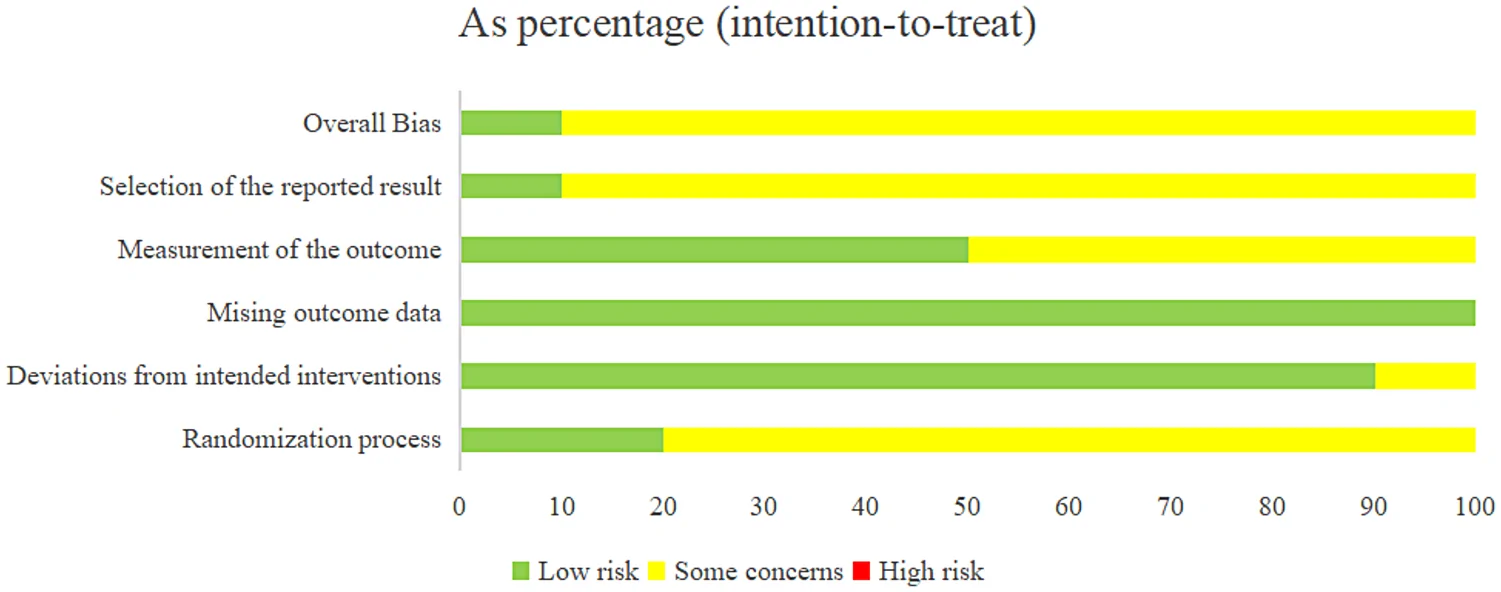
Risk-of-bias assessment of the 10 included randomized controlled trials using the Cochrane RoB 2 tool. Bars show the percentage of studies rated as low risk, some concerns, or high risk for each domain and for overall bias. Study-level judgments are presented in Supplementary Figure S1.

### 3.4 Results of the network meta-analysis

#### 3.4.1 Network evidence plots

Figure 3 illustrates the geometry of the treatment network for each safety outcome. Each node represents a specific intervention, and the node size is proportional to the total number of participants assigned to that intervention. The connecting lines, or edges, indicate direct head-to-head comparisons, and their thickness corresponds to the number of trials contributing to each comparison. All outcome networks were star-shaped, with conventional therapy as the common comparator; no closed loops were identified. Therefore, loop inconsistency could not be formally assessed for any outcome. Between-study heterogeneity was modeled using a common network-wide *τ*^2^ estimated across all comparisons in the random-effects NMA. The estimated *τ*^2^ values were 0.04 for any-grade adverse events, 0 for grade ≥3 adverse events, 0 for serious adverse events, 1.52 for fatal adverse events, 0.02 for any-cause adverse events, 0.11 for diarrhea, 0 for rash, 0 for hypothyroidism, and 0 for fatigue (Supplementary Table S1-7). *I*^2^ was not reported because it is not a standard output of the NMA and is not reliably estimable with sparse networks; *τ*^2^ and qualitative assessment were used instead. These estimates should be interpreted cautiously because several networks were sparse.

**Figure 3:**
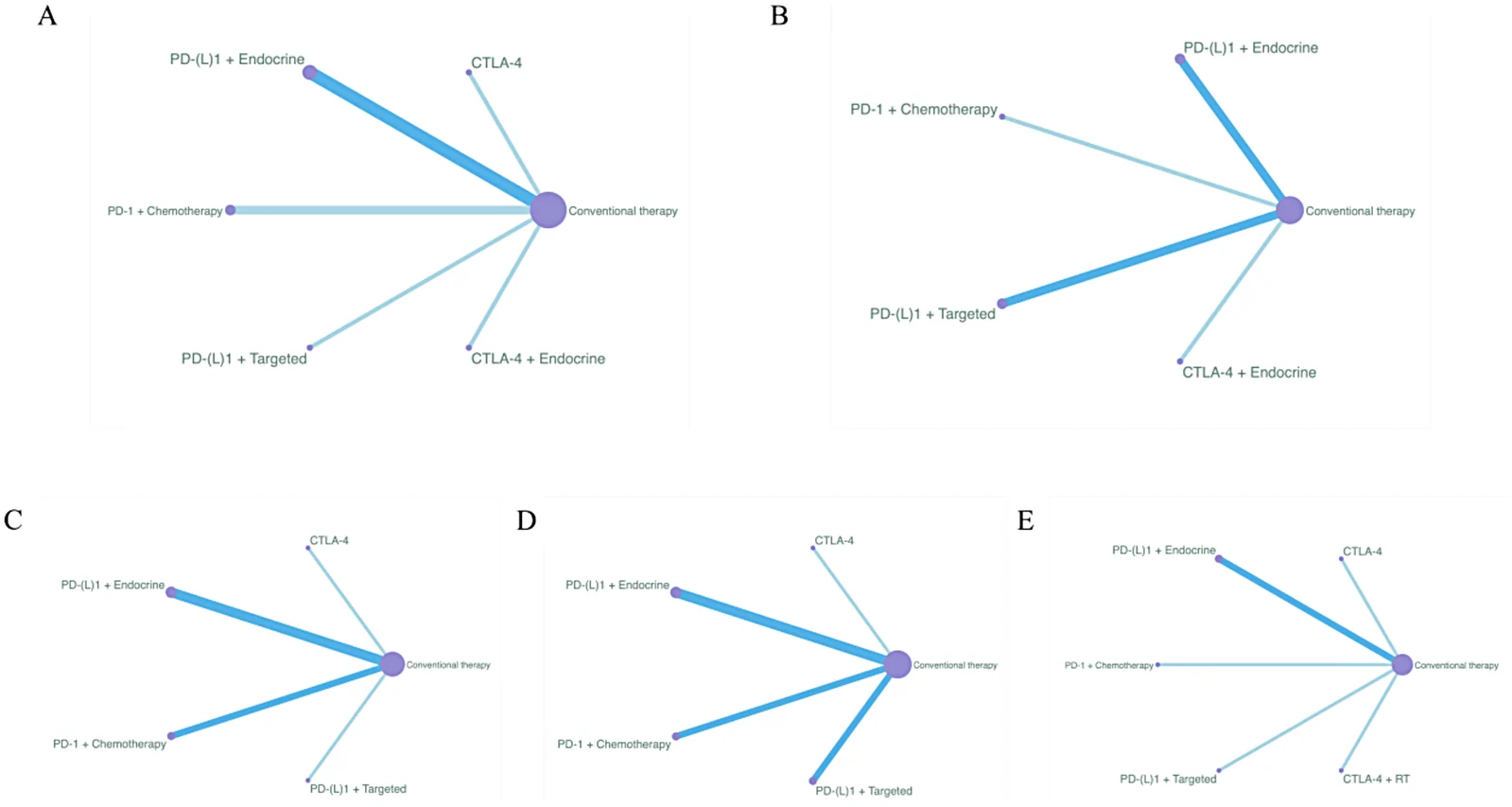
Network plots for **(A)** any-grade adverse events, **(B)** grade ≥3 adverse events, **(C)** serious adverse events, **(D)** fatal adverse events, and **(E)** any-cause adverse events. The size of each node represents the number of participants receiving the corresponding intervention, and the thickness of the lines connecting interventions represents the number of studies included in each comparison.

#### 3.4.2 Any-grade adverse events

For the risk of any-grade adverse events, six interventions were analyzed. A total of 8 studies involving 6,791 patients were included, and the corresponding network plot is shown in Figure 3A. Conventional therapy was associated with a lower risk of any-grade adverse events than PD-(L)1 plus endocrine therapy [OR = 0.34, 95% CI (0.26–0.45)], CTLA-4 [OR = 0.22, 95% CI (0.13–0.38)], and PD-(L)1 plus targeted therapy [OR = 0.14, 95% CI (0.08–0.23)] (all *P* < 0.05). However, because comparisons with conventional therapy provide limited information for differentiating among ICI-based regimens, we focused primarily on pairwise comparisons among the other ICI-based regimens. Specifically, several treatment strategies, including PD-(L)1 plus endocrine therapy [OR = 0.32, 95% CI (0.19–0.55)], CTLA-4 [OR = 0.21, 95% CI (0.10–0.43)], and PD-(L)1 plus targeted therapy [OR = 0.13, 95% CI (0.07–0.26)], were associated with a higher risk of any-grade adverse events than PD-1 plus chemotherapy (all *P* < 0.05). Furthermore, PD-(L)1 plus targeted therapy was also associated with a higher risk of any-grade adverse events than PD-(L)1 plus endocrine therapy [OR = 0.41, 95% CI (0.22–0.74)] (*P* < 0.05). The results of the pairwise comparisons are presented in Supplementary Table S2.

According to the SUCRA-based analysis, among the interventions included in this comparison, PD-1 plus chemotherapy achieved the highest SUCRA value for this outcome (91%, Rank 1), indicating the lowest incidence of adverse events, followed by conventional therapy (87.4%, Rank 2) and PD-(L)1 plus endocrine therapy (55.4%, Rank 3). In contrast, CTLA-4 plus endocrine therapy had the lowest SUCRA value (15.5%, Rank 6), corresponding to the highest adverse event incidence rate, while PD-(L)1 plus targeted therapy (15.7%, Rank 5) and CTLA-4 monotherapy (35%, Rank 4) also exhibited relatively high adverse event rates. It should be emphasized that SUCRA rankings reflect only the relative probabilistic hierarchy of interventions; statistically significant differences between interventions should still be determined based on the effect estimates and their 95% confidence intervals. The ranking results for all interventions are shown in Figure 4A, and the complete SUCRA values and rankings for all outcomes are presented in Table 1.

**Figure 4:**
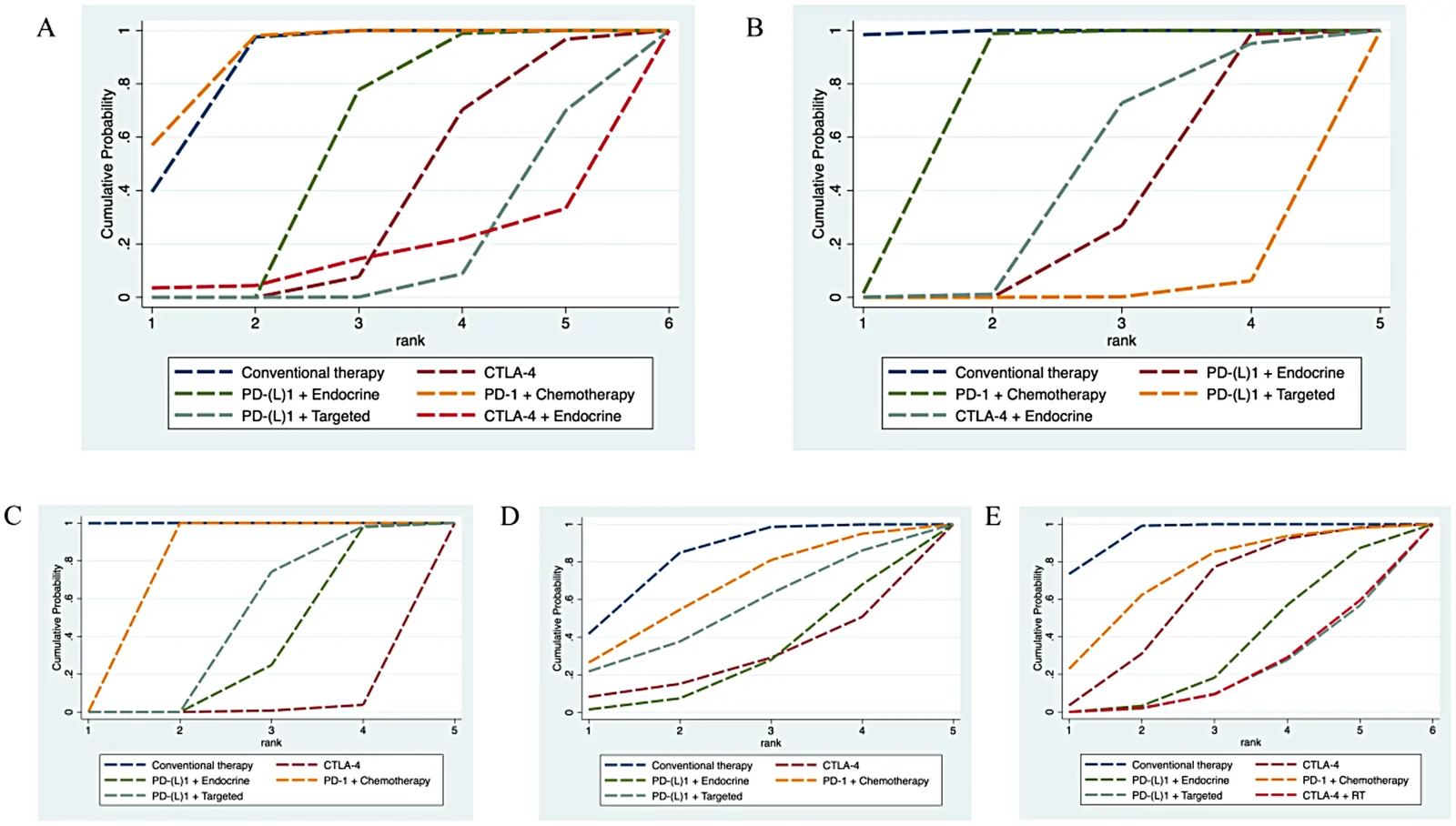
Cumulative ranking curves for any-grade adverse events **(A)**, grade ≥3 adverse events **(B)**, serious adverse events **(C)**, fatal adverse events **(D)**, and any-cause adverse events **(E)**

**Table 1:** Ranking of safety of each treatment.—, not available. Data are presented as SUCRA values (%) and ranks.

Treatment	Any-grade adverse events	Rank	Grade ≥3 adverse events	Rank	Serious adverse events	Rank	Fatal adverse events	Rank	Any-cause adverse events	Rank
Conventional therapy	87.4%	2	99.7%	1	100%	1	81.4%	1	94.5%	1
CTLA-4	35%	4	–	–	1.1%	5	25.8%	5	60.5%	3
PD-(L)1 + Endocrine	55.4%	3	31.5%	4	30.7%	4	26.2%	4	33.2%	4
PD-1 + Chemotherapy	91%	1	75.1%	2	75%	2	64.4%	2	72.5%	2
PD-(L)1 + Targeted	15.7%	5	1.7%	5	43.2%	3	52.2%	3	19.2%	6
CTLA-4 + RT	–	–	–	–	–	–	–	–	20.1%	5
CTLA-4 + Endocrine	15.5%	6	42%	3	–	–	–	–	–	–

#### 3.4.3 Grade ≥3 adverse events

For grade ≥3 adverse events, five treatment interventions were evaluated in this comparison; the corresponding network plot is shown in Figure 3B. Compared with conventional therapy, several ICI-containing regimens were associated with a higher risk of grade ≥3 adverse events (all *P* < 0.05). PD-1 plus chemotherapy was associated with a lower risk of grade ≥3 adverse events than CTLA-4 plus endocrine [OR = 0.41, 95% CI (0.19–0.88)], PD-(L)1 plus endocrine therapy [OR = 0.32, 95% CI (0.23–0.44)], and PD-(L)1 plus targeted therapy [OR = 0.21, 95% CI (0.14–0.32)] (all *P* < 0.05; Supplementary Table S3). In the SUCRA analysis, PD-1 plus chemotherapy had a relatively high SUCRA value for grade ≥3 adverse events, whereas PD-(L)1 plus targeted therapy had a relatively low SUCRA value (Figure 4B).

#### 3.4.4 Serious adverse events

For serious adverse events, five treatment interventions were analyzed in this comparison, and the corresponding network plot is shown in Figure 3C. Conventional therapy was associated with a lower risk of serious adverse events than PD-1 plus chemotherapy [OR = 0.69, 95% CI (0.55–0.86)], PD-(L)1 plus targeted therapy [OR = 0.21, 95% CI (0.11–0.42)], PD-(L)1 plus endocrine therapy [OR = 0.16, 95% CI (0.12–0.23)], and CTLA-4 [OR = 0.07, 95% CI (0.03–0.16)] (all *P* < 0.05). Other pairwise comparisons indicated that PD-1 plus chemotherapy was associated with a lower risk of serious adverse events than PD-(L)1 plus targeted therapy [OR = 0.31, 95% CI (0.15–0.64)], PD-(L)1 plus endocrine therapy [OR = 0.24, 95% CI (0.16–0.35)] and CTLA-4 [OR = 0.10, 95% CI (0.05–0.23)] (all *P* < 0.05). Compared with CTLA-4, PD-(L)1 plus targeted therapy [OR = 0.33, 95% CI (0.12–0.94)] was associated with a lower risk of serious adverse events (*P* < 0.05; Supplementary Table S4). For serious adverse event incidence, conventional therapy (CT) had the highest SUCRA value (100%, Rank 1), suggestive of the lowest serious adverse event risk. By contrast, CTLA-4 monotherapy showed the lowest SUCRA value (1.1%, Rank 5), with the highest serious adverse event incidence (Figure 4C).

#### 3.4.5 Fatal adverse events

For fatal adverse events, five treatment interventions were analyzed in this comparison, and the corresponding network plot is shown in Figure 3D. Compared with conventional therapy, no treatment intervention was associated with a significantly lower risk of fatal adverse events (all *P* > 0.05; Supplementary Table S5). In the SUCRA-based hierarchy analysis for fatal adverse event incidence, conventional therapy had the highest SUCRA value (81.4%, Rank 1), suggestive of the lowest fatal adverse event incidence, whereas CTLA-4 monotherapy had the lowest SUCRA value (25.8%, Rank 5), corresponding to the highest fatal adverse event incidence (Figure 4D).

#### 3.4.6 Any-cause adverse events

For any-cause adverse events, six treatment interventions were analyzed in this comparison, and the corresponding network plot is shown in Figure 3E. Only comparisons involving conventional therapy reached statistical significance. Conventional therapy was associated with a lower risk of any-cause adverse events than PD-(L)1 plus endocrine therapy [OR = 0.32, 95% CI (0.18–0.57)], CTLA-4 plus radiotherapy [OR = 0.24, 95% CI (0.11–0.57)], and PD-(L)1 plus targeted therapy [OR = 0.24, 95% CI (0.11–0.55)] (all *P* < 0.05; Supplementary Table S6), whereas all other pairwise comparisons were not statistically significant. Because most comparisons were not statistically significant, SUCRA rankings for this outcome should be regarded as exploratory and should not replace inference based on effect estimates and confidence intervals. Therefore, interpretation of this outcome was based primarily on pairwise comparison results, with SUCRA used only as an exploratory reference. Although conventional therapy (94.5%, Rank 1) and PD-1 plus chemotherapy (72.5%, Rank 2) had the highest SUCRA values, these results should be interpreted only as exploratory ranking trends rather than evidence of definitive between-treatment differences (Figure 4E).

### 3.5 Supplementary analysis of organ-specific immune-related adverse events

The overall adverse event analyses described above evaluated the general safety risks associated with different ICI-based regimens, whereas differences in organ-specific immune-related adverse events (irAEs) are also clinically relevant. Further supplementary analyses of common irAEs, including diarrhea, rash, hypothyroidism, and fatigue, showed that different combination strategies exhibited distinct patterns of organ-specific toxicity. For diarrhea, PD-1 plus chemotherapy was associated with a lower risk than CTLA-4 [OR = 0.25, 95% CI (0.09–0.72)], PD-(L)1 plus targeted therapy [OR = 0.20, 95% CI (0.06–0.72)] and CTLA-4 plus endocrine therapy [OR = 0.12, 95% CI (0.02–0.59)]. PD-(L)1 plus endocrine therapy was associated with a lower risk than CTLA-4 plus endocrine therapy [OR = 0.21, 95% CI (0.04–0.96)]. Conventional therapy was associated with a lower risk than most of the ICI regimens including PD-(L)1 plus endocrine therapy [OR = 0.49, 95% CI (0.28–0.84)], CTLA-4 [OR = 0.21, 95% CI (0.09–0.46)], PD-(L)1 plus targeted therapy [OR = 0.17, 95% CI (0.06–0.49)] and CTLA-4 plus endocrine therapy [OR = 0.10, 95% CI (0.02–0.42)] (Supplementary Table S7). SUCRA analysis likewise indicated that, among all included regimens, conventional therapy had the lowest risk of diarrhea (93.7%, Rank 1), followed by PD-1 plus chemotherapy (83.7%, Rank 2) and PD-(L)1 plus endocrine therapy (60.1%, Rank 3); CTLA-4 plus endocrine therapy had the highest risk of diarrhea (10.3%, Rank 6), while PD-(L)1 plus targeted therapy also showed a relatively high risk (22.4%, Rank 5) (Supplementary Figures S2–S4, Table 2).

**Table 2:** Ranking of safety of each treatment. —, not available. Data are presented as SUCRA values (%) and ranks.

Treatment	Diarrhea	Rank	Rash	Rank	Hypothyroidism	Rank	Fatigue	Rank
Conventional therapy	93.7%	1	99.7%	1	93.5%	1	88.4%	1
CTLA-4	29.7%	4	37.7%	3	–	–	20.2%	5
PD-(L)1 + Endocrine	60.1%	3	53.7%	2	32.2%	3	62%	3
PD-1 + Chemotherapy	83.7%	2	–	–	–	–	86.3%	2
PD-(L)1 + Targeted	22.4%	5	8.9%	4	2.3%	4	27.6%	4
CTLA-4 + RT	–	–	–	–	–	–	–	–
CTLA-4 + Endocrine	10.3%	6	-	-	72%	2	15.5%	6

For rash, PD-(L)1 plus endocrine therapy [OR = 0.20, 95% CI (0.15–0.27)], CTLA-4 [OR = 0.16, 95% CI (0.09–0.29)] and PD-(L)1 plus targeted therapy [OR = 0.04, 95% CI (0.00–0.58)] were associated with a higher risk than conventional therapy (Supplementary Table S8). Correspondingly, conventional therapy had the highest SUCRA value for rash (99.7%, Rank 1), whereas PD-(L)1 plus targeted therapy had the lowest SUCRA value (8.9%, Rank 4) (Supplementary Figures S5–S7, Table 2).

For hypothyroidism, PD-(L)1 plus endocrine therapy [OR = 0.27, 95% CI (0.18–0.39)] and PD-(L)1 plus targeted therapy [OR = 0.08, 95% CI (0.02–0.33)] were associated with a significantly higher risk than conventional therapy; PD-(L)1 plus targeted therapy was also associated with a significantly higher risk than CTLA-4 plus endocrine therapy [OR = 0.12, 95% CI (0.02–0.63)] (Supplementary Table S9). SUCRA analysis suggested that conventional therapy had the highest SUCRA value for hypothyroidism, whereas PD-(L)1 plus targeted therapy had the lowest SUCRA value (Supplementary Figures S8–S10).

For fatigue, PD-(L)1 plus targeted therapy [OR = 0.57, 95% CI (0.39–0.83)], CTLA-4 [OR = 0.51, 95% CI (0.32–0.81)] and CTLA-4 plus endocrine therapy [OR = 0.45, 95% CI (0.22–0.93)] were associated with a significantly higher risk than conventional therapy. PD-(L)1 plus targeted therapy [OR = 0.57, 95% CI (0.36–0.91)], CTLA-4 [OR = 0.51, 95% CI (0.30–0.87)] and CTLA-4 + Endocrine [OR = 0.45, 95% CI (0.21–0.97)] also had a significantly higher risk than PD-1 plus chemotherapy, and CTLA-4 was associated with a significantly higher risk than PD-(L)1 plus endocrine therapy [OR = 0.60, 95% CI (0.36–0.99)] (Supplementary Table S10). SUCRA analysis showed that conventional therapy had the highest SUCRA value for fatigue, whereas CTLA-4 plus endocrine therapy had the lowest SUCRA value (Supplementary Figures S11–S13).

Table 2 presents the complete SUCRA values and treatment rankings for all organ-specific irAE outcomes. It should be noted that SUCRA rankings reflect only the relative probabilistic hierarchy among interventions and do not directly indicate statistical significance. Therefore, interpretation should still be based on the effect estimates and their 95% confidence intervals.

### 3.6 Subgroup and sensitivity analyses of endocrine therapy

In prostate cancer, “endocrine therapy” refers to systemic treatment targeting the androgen axis, including androgen deprivation therapy (ADT) and androgen receptor pathway inhibitors (ARIs) such as enzalutamide (30). Because the preceding network meta-analysis suggested potentially less favorable safety signals for the PD-(L)1 plus endocrine therapy node across several outcomes, we performed a subgroup analysis of endocrine-based combination regimens, supplemented by sensitivity analyses, to evaluate the consistency of these findings and explore potential sources of heterogeneity.

This subgroup included studies that compared the safety of adding an ICI vs. not adding an ICI to an ARPI-based androgen-axis therapy. The ARPI backbone was enzalutamide in three trials (22, 23, 27) and abiraterone plus apalutamide in one trial (29). ADT was administered concurrently in some studies, and the ICIs included pembrolizumab, atezolizumab, or ipilimumab. Therefore, in the present study, the “ICI plus endocrine therapy” subgroup refers to comparisons of safety outcomes according to whether an ICI was added to an ARPI-based endocrine backbone, and is not restricted to enzalutamide or to PD-(L)1 inhibitors.

Random-effects models were used for all endocrine therapy subgroup analyses, and heterogeneity was quantified using I^2^ statistics. For any-grade adverse events, four studies were included (22, 23, 27, 29). Heterogeneity was low (*I*^2^ = 0.0%, *P* = 0.530), and the pooled analysis showed that ICI plus endocrine therapy significantly increased the risk of any-grade adverse events (OR = 1.33, 95% CI 1.19–1.47; Supplementary Figure S14). Sensitivity analysis showed a consistent direction of effect; however, owing to the limited number of included studies, stability could not be established and precision was limited (Supplementary Figure S15). For grade ≥3 adverse events, three studies were included (22, 23, 29). Heterogeneity was low (*I*^2^ = 0.0%, *P* = 0.482), and the pooled analysis showed that this combination significantly increased the risk of grade ≥3 adverse events (OR = 2.85, 95% CI 2.36–3.45; Supplementary Figure S16). Sensitivity analysis demonstrated only minor changes in the pooled effect, indicating that the direction and statistical significance were largely unchanged, but precision was limited by the small number of studies (Supplementary Figure S17). For serious adverse events, three studies were included (22, 23, 27). Heterogeneity was low (*I*^2^ = 0.0%, *P* = 0.636), and the pooled analysis showed that ICI plus endocrine therapy was significantly associated with an increased risk of serious adverse events (OR = 5.33, 95% CI 3.89–7.31; Supplementary Figure S18). Sensitivity analysis showed that both the direction and statistical significance of the effect remained consistent (Supplementary Figure S19). For fatal adverse events, three studies were included (22, 23, 27). Heterogeneity was also low (*I*^2^ = 0.0%, *P* = 0.953), and the pooled analysis showed a statistically significant but imprecise increase in fatal AEs (OR = 8.03, 95% CI 1.84–35.02; Supplementary Figure S20), based on few events. Sensitivity analysis suggested that the direction of the result remained consistent, although the confidence intervals were relatively wide, indicating limited precision of the effect estimate for this low-frequency outcome (Supplementary Figure S21). For any-cause adverse events, only two studies were included (22, 27). Heterogeneity was low (*I*^2^ = 0.0%, *P* = 0.923), and the pooled analysis showed no significant difference between ICI plus endocrine therapy and control (OR = 1.04, 95% CI 0.92–1.18; Supplementary Figure S22). Leave-one-out sensitivity analysis showed that the pooled OR remained consistently close to 1, the pooled estimate remained similar, but this analysis included only two studies (Supplementary Figure S23).

Overall, ICI plus endocrine therapy was associated with an increased risk across any-grade, grade ≥3, serious, and fatal adverse events. These findings suggest that, within an ARPI-based endocrine treatment framework, the addition of ICIs may impose a relatively consistent additional safety burden. In contrast, the lack of a significant difference for any-cause adverse events may reflect the limited number of studies and the high baseline rate of any-cause events in both arms. Considering that the background endocrine regimens, namely enzalutamide ± ADT, and the types of ICIs used were not entirely identical across the included studies, and that any-grade adverse events and adverse event-related discontinuation are more readily affected by study implementation and assessment methods, these factors may represent important sources of heterogeneity. However, their exact contribution should be interpreted cautiously. It should also be noted that this subgroup included only two to four studies; therefore, funnel plots had very limited ability to detect publication bias and should be interpreted descriptively (Supplementary Figures S24–S28). The power of sensitivity analyses to assess the direction and stability of effect estimates was also restricted. Therefore, these findings should be regarded primarily as descriptive and supportive evidence and interpreted with caution. No further subgroup analyses were performed, mainly because the limited number of studies available for some outcomes would have resulted in sparse data after additional stratification, thereby reducing the stability and interpretability of the results.

### 3.7 Assessment of publication bias

Funnel plots were generated for each outcome, with each data point representing an individual study estimate. Visual inspection did not suggest marked asymmetry in most funnel plots. However, because the number of included studies was limited for some outcomes and several outlying estimates were observed, the ability of the funnel plots to detect small-study effects or publication bias was limited. Therefore, these findings should be interpreted with caution (Figure 5).

**Figure 5:**
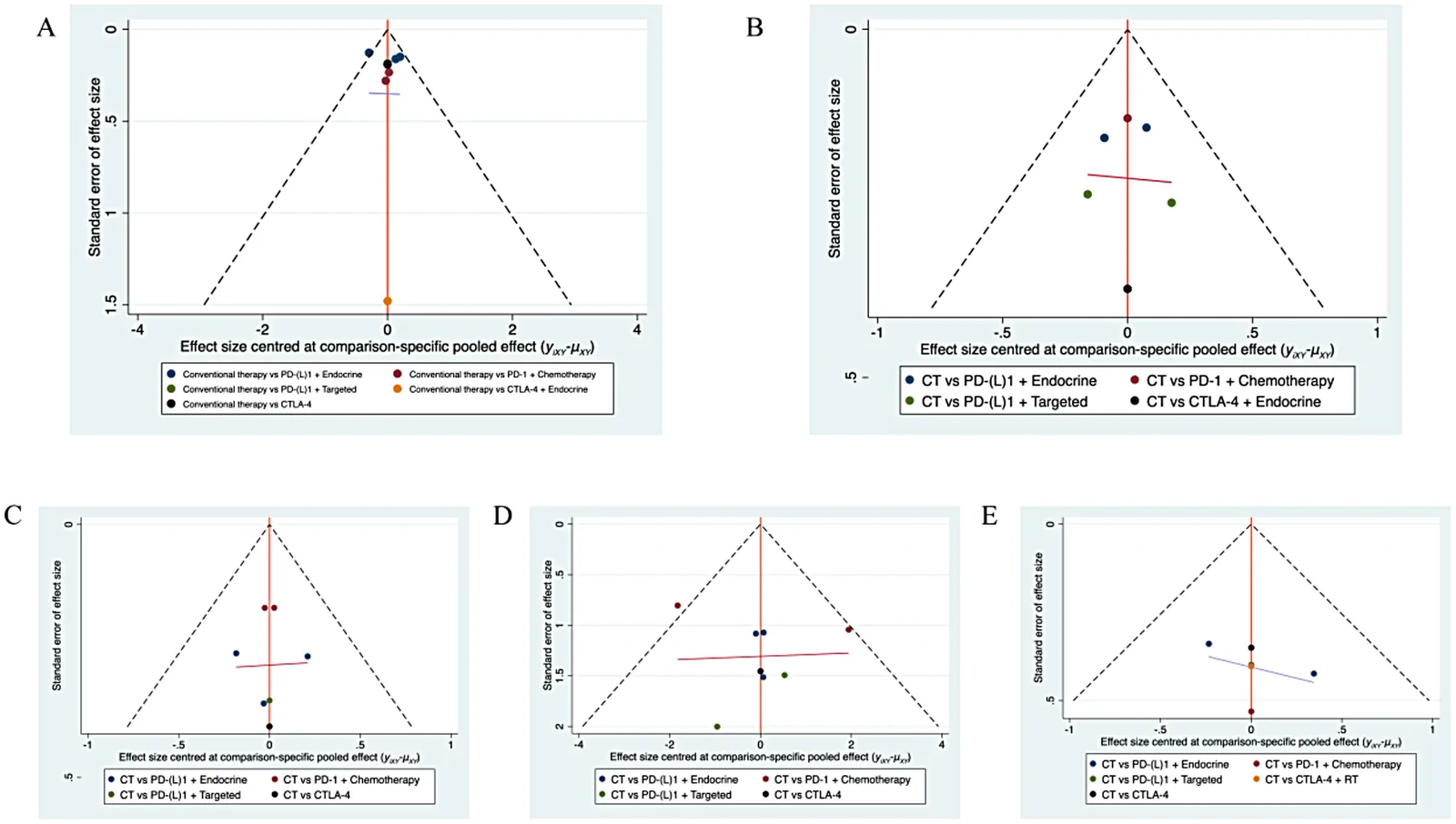
Comparison-adjusted funnel plots for each safety outcome: any-grade adverse events **(A)**, grade ≥3 adverse events **(B)**, serious adverse events **(C)**, fatal adverse events **(D)**, and any-cause adverse events **(E)**. CT, conventional therapy.

### 3.8 Sensitivity analysis after excluding mHSPC

To examine whether the inclusion of studies involving metastatic hormone-sensitive prostate cancer (mHSPC) influenced the overall safety estimates, we performed a sensitivity analysis by excluding mHSPC-related studies and re-estimating the available pairwise comparisons. The results are summarized in Table 3. Before excluding mHSPC, PD-1 plus chemotherapy was associated with a lower risk of any-grade adverse events than PD-(L)1 plus targeted therapy (OR 0.13, 95% CI 0.07–0.26); after exclusion, this association was no longer statistically significant and the direction of the effect changed (OR 1.60, 95% CI 0.76–3.38). Similarly, PD-1 plus chemotherapy was associated with a lower risk of grade ≥3 adverse events than CTLA-4 plus endocrine therapy before exclusion (OR 0.41, 95% CI 0.19–0.88); after exclusion, this estimate was attenuated and no longer statistically significant (OR 1.15, 95% CI 0.52–2.56). For serious adverse events, the comparison between PD-(L)1 plus endocrine therapy and PD-(L)1 plus targeted therapy yielded an OR of 0.77 (95% CI 0.36–1.62) before exclusion and 0.10 (95% CI 0.05–0.23) after exclusion. No statistical changes were observed for fatal adverse events, any-cause adverse events, diarrhea, and fatigue before and after excluding mHSPC studies. For organ-specific immune-related adverse events, most comparisons for rash and hypothyroidism showed markedly wider confidence intervals after exclusion and were no longer statistically significant, although the point estimates were largely unchanged (e.g., rash for PD-(L)1 plus targeted therapy vs. conventional therapy: OR 0.04 [95% CI 0.00–0.58] before exclusion vs. 0.04 [95% CI 0.00–7.56] after; hypothyroidism for PD-(L)1 plus targeted therapy vs. conventional therapy: OR 0.08 [95% CI 0.02–0.33] vs. 0.08 [95% CI 0.01–1.08]). Overall, these findings suggest that excluding mHSPC studies may affect the precision and, for some comparisons, the direction or statistical significance of safety estimates. Therefore, the results of this sensitivity analysis should be interpreted cautiously and regarded as exploratory.

**Table 3:** Sensitivity analysis of safety outcomes before and after excluding mHSPC studies.

Adverse events type	Treatment measures	Before excluding mHSPC	After excluding mHSPC
Any-grade adverse events	PD-1 + Chemotherapy vs. PD-(L)1 + Targeted	0.13 (0.07, 0.26)	1.60 (0.76, 3.38)
Grade ≥3 adverse events	PD-1 + Chemotherapy vs. CTLA-4 + Endocrine	0.41 (0.19, 0.88)	1.15 (0.52, 2.56)
Serious adverse events	PD-(L)1 + Targeted vs. PD-(L)1 + Endocrine	0.77 (0.36, 1.62)	0.10 (0.05, 0.23)
Fatal adverse events	NA	NA	NA
Any-cause adverse events	NA	NA	NA
Diarrhea	NA	NA	NA
Rash	Conventional therapy vs. PD-(L)1 + Targeted	0.04 (0.00, 0.58)	0.04 (0.00, 7.56)
Rash	Conventional therapy vs. PD-(L)1 + Endocrine	0.20 (0.15, 0.27)	0.18 (0.00, 1,175.80)
Rash	Conventional therapy vs. CTLA-4	0.16 (0.09, 0.29)	0.16 (0.00, 655.36)
Hypothyroidism	Conventional therapy vs. PD-(L)1 + Endocrine	0.27 (0.18, 0.39)	0.27 (0.05, 1.52)
Hypothyroidism	Conventional therapy vs. PD-(L)1 + Targeted	0.08 (0.02, 0.33)	0.08 (0.01, 1.08)
Hypothyroidism	CTLA-4 + Endocrine vs. PD-(L)1 + Targeted	0.12 (0.02, 0.63)	0.12 (0.00, 3.31)
Fatigue	NA	NA	NA

Values are odds ratios (ORs) with 95% confidence intervals (CIs). mHSPC, metastatic hormone-sensitive prostate cancer.

## 4 Discussion

### 4.1 Summary of the main findings

This study synthesized 10 randomized comparative trials involving 8,197 participants and indirectly compared adverse-event risks across ICI-based strategies in metastatic prostate cancer. Safety rankings varied by outcome, and no regimen was consistently ranked as safest. According to Table 1, conventional therapy had the highest SUCRA values for grade ≥3 adverse events (99.7%), serious adverse events (100%), fatal adverse events (81.4%), and any-cause adverse events (94.5%), and ranked second for any-grade adverse events (87.4%). Among ICI-containing regimens, PD-1 plus chemotherapy showed relatively favorable exploratory rankings across outcomes: any-grade (91%), grade ≥3 (75.1%), serious (75%), fatal (64.4%), and any-cause adverse events (72.5%). CTLA-4-containing regimens generally showed less favorable rankings, particularly CTLA-4 monotherapy for serious (1.1%) and fatal (25.8%) adverse events, and CTLA-4 plus endocrine therapy for any-grade (15.5%) and grade ≥3 (42%) adverse events. PD-(L)1 plus targeted therapy ranked poorly for any-grade (15.7%), grade ≥3 (1.7%), and any-cause (19.2%) adverse events but was intermediate for serious (43.2%) and fatal (52.2%) adverse events. PD-(L)1 plus endocrine therapy showed intermediate rankings across outcomes.

For organ-specific immune-related adverse events (Table 2), toxicity patterns also differed by regimen. Conventional therapy had the highest SUCRA values for diarrhea, rash, hypothyroidism, and fatigue. PD-1 plus chemotherapy ranked high for diarrhea (83.7%) and fatigue (86.3%); PD-(L)1 plus endocrine therapy was intermediate for most organ-specific outcomes; PD-(L)1 plus targeted therapy ranked low for most irAEs; and CTLA-4 plus endocrine therapy ranked lowest for diarrhea (10.3%) and fatigue (15.5%) but highest among ICI regimens for hypothyroidism (72%). These findings suggest that safety evaluation should integrate specific toxicity patterns rather than rely on a single overall endpoint. SUCRA values should be regarded as exploratory and hypothesis-generating.

### 4.2 Comparison with previous studies

Systematic reviews and meta-analyses of immune checkpoint inhibitors (ICIs) in prostate cancer have increased in recent years. However, most previous reviews focused on the overall efficacy or safety of ICIs. Their comparative frameworks generally remained at the level of “ICI vs. non-ICI treatment,” which limited their ability to assess relative safety differences among specific combination strategies. The meta-analysis by Noori et al. (31), which included various prostate cancer populations, suggested that ICIs may increase the risk of certain treatment-related adverse events. However, because it was based mainly on conventional pairwise pooling, it could not provide comparative rankings across different combination regimens. The meta-analysis by Zhang et al. (32), which focused on metastatic castration-resistant prostate cancer (mCRPC), placed greater emphasis on survival benefit and overall safety. It suggested that ICIs provide limited efficacy in unselected populations but did not further clarify differences in adverse event patterns among distinct combination strategies. Hong et al. (33) specifically examined the efficacy and safety of PD-1/PD-L1 inhibitors in mCRPC and provided a more systematic summary of immune-related adverse events. Nevertheless, CTLA-4-based regimens were not included, and comprehensive comparisons across different treatment backgrounds, such as chemotherapy, radiotherapy, targeted therapy, endocrine therapy, and dual immunotherapy, were not performed. In contrast, the present study used a network meta-analysis approach, which allowed simultaneous comparison of multiple ICI-related treatment strategies in the absence of head-to-head trials and thereby partly addressed the limitations of conventional pairwise meta-analyses in multi-regimen ranking. Furthermore, this study incorporated not only overall safety outcomes, including any-grade adverse events, grade ≥3 adverse events, serious adverse events, fatal adverse events, and any-cause adverse events, but also organ-specific immune-related adverse events, such as diarrhea, rash, hypothyroidism, and fatigue. In doing so, this study provides a more comprehensive characterization of the types and distribution patterns of adverse events across regimens. Therefore, compared with previous studies, the present work more directly addresses the clinically relevant question of the relative safety and major adverse-event risks of different ICI-based combination strategies, although the certainty of many comparisons remains limited because of sparse networks and predominantly indirect evidence.

### 4.3 Mechanistic interpretation

Immune checkpoint inhibitor-related adverse events are generally considered to arise from non-tumor-specific immune activation after disruption of immune tolerance. Compared with PD-1/PD-L1 inhibitors, which primarily act in peripheral tissues and the tumor microenvironment and generally have a more manageable safety profile, CTLA-4 inhibitors act during the initial phase of T-cell activation. They may therefore be more likely to induce broader immune activation, leading to a higher incidence of high-grade or multi-organ adverse events (34–36). A similar pattern has been observed in other solid tumors. For example, CheckMate 067 showed that nearly 60% of patients with advanced melanoma treated with ipilimumab plus nivolumab experienced grade 3–4 adverse events. Similarly, in non-small cell lung cancer, CTLA-4-containing regimens have consistently been associated with a higher incidence of severe toxicity (37–39). The findings of the present study are consistent with this body of evidence, suggesting that CTLA-4-containing regimens are generally associated with less favorable safety profiles in metastatic prostate cancer.

Meanwhile, the network meta-analysis showed that safety profiles varied across ICI-containing regimens. PD-1 plus chemotherapy showed relatively high SUCRA values for any-grade adverse events (91%, rank 1), grade ≥3 adverse events (75.1%, rank 2), serious adverse events (75%, rank 2), fatal adverse events (64.4%, rank 2), and any-cause adverse events (72.5%, rank 2), suggesting a potentially more favorable exploratory safety profile among ICI-based regimens. In contrast, CTLA-4-containing regimens and PD-(L)1 plus endocrine therapy showed less favorable safety signals, particularly for serious and fatal adverse events [e.g., CTLA-4: serious 1.1%, rank 5; fatal 25.8%, rank 5; PD-(L)1 plus endocrine therapy: serious 30.7%, rank 4; fatal 26.2%, rank 4]. Notably, the safety profile of PD-(L)1 plus endocrine therapy was not consistent across all adverse-event categories. This regimen was associated with a higher estimated risk of fatal adverse events, whereas its rankings for organ-specific immune-related adverse events were variable (e.g., hypothyroidism 32.2%, rank 3; diarrhea 60.1%, rank 3). One possible, but unconfirmed, explanation is a competing-risk mechanism, whereby early severe toxicity and death may reduce the opportunity to observe delayed immune-related adverse events. The IMbassador250 trial was terminated early because it failed to demonstrate a survival benefit, and serious treatment-related deaths were reported (27). However, this interpretation remains speculative because patient-level time-to-event safety data were unavailable in the present study (40).

In addition, metastatic prostate cancer is widely regarded as a prototypical “cold tumor,” and ICI monotherapy alone often fails to achieve satisfactory efficacy. Accordingly, ICIs are frequently combined in clinical practice with chemotherapy, radiotherapy, targeted therapy, or endocrine therapy. However, although such combinations may enhance antitumor efficacy, they may also impose an additional safety burden (41, 42). In this context, the relatively high SUCRA values observed for PD-1 plus chemotherapy in analyses of any-grade adverse events (91%, rank 1), grade ≥3 adverse events (75.1%, rank 2), serious adverse events (75%, rank 2), fatal adverse events (64.4%, rank 2), and any-cause adverse events (72.5%, rank 2) may be related to differences in concomitant treatment and baseline patient characteristics (23). This finding is not entirely consistent with some phase III studies reporting a higher incidence of grade 3–4 adverse events with docetaxel plus PD-1 inhibitors (28, 43). One possible, but unconfirmed, explanation is that taxane-based chemotherapy is commonly accompanied by systemic glucocorticoids, which may attenuate acute immune activation. However, this hypothesis remains speculative because patient-level data on glucocorticoid exposure, dose intensity, treatment duration, and timing of adverse events were unavailable. From the perspective of toxicity patterns, this finding may suggest that adverse events associated with this regimen are more likely to present as relatively manageable hematologic toxicities, such as neutropenia, rather than as refractory or life-threatening immune-mediated injuries (44).

### 4.4 Clinical implications

The clinical implications of the present study can be considered from several perspectives. First, in patients with metastatic prostate cancer, safety should be considered alongside efficacy because it directly affects treatment accessibility, adherence, and continuity. Because these patients are typically older, often have multiple comorbidities, and frequently receive complex treatment regimens, clinical evaluation of ICI-based therapies should consider not only potential therapeutic benefits but also the risk of adverse events. Second, this study demonstrated that the burden of adverse events differed across ICI-based combination strategies, with CTLA-4-containing regimens and PD-(L)1 plus endocrine therapy being associated with higher risks of serious and fatal adverse events. These findings suggest that, in clinical practice, comprehensive assessment of baseline patient risk, treatment tolerability, and toxicity monitoring strategies is particularly important. Finally, the present results further suggest that, in unselected populations, broad empiric ICI combination strategies may not necessarily confer a more favorable risk–benefit profile and may instead impose an additional toxicity burden (45). Future studies should further focus on populations most likely to benefit and comprehensively evaluate the treatment-related harm and potential benefits of different combination regimens. Such efforts may help more precisely define the appropriate role and risk–benefit characteristics of ICI therapy in metastatic prostate cancer (46, 47). However, because many comparisons were indirect, the networks were sparse, and several estimates had wide confidence intervals, these clinical implications should be regarded as exploratory and should not be used to declare any regimen as definitively “safest” or clinically superior in safety.

### 4.5 Limitations and future directions

Several limitations of this study should be acknowledged. First, all analyzed networks were star-shaped and no closed loops were present, which made it impossible to formally assess loop inconsistency. The transitivity assumption may also be challenged by differences in disease state (mHSPC vs. mCRPC), line of therapy, prior chemotherapy, background androgen-axis therapy, age, performance status, treatment duration, and duration of safety follow-up. We therefore performed a sensitivity analysis excluding mHSPC-related studies; this changed the precision and, for some comparisons, the direction or statistical significance of the estimates (Table 3), further supporting a cautious interpretation. In addition, several treatment nodes were relatively broad, especially conventional therapy, and the specific regimens, background therapies, and disease settings assigned to each node are listed in Supplementary Table S1-2. Narrower node-definition sensitivity analyses were not feasible because of sparse data. Therefore, the ranking results should be regarded as hypothesis-generating rather than as a definitive basis for clinical decision-making.

Second, the final analysis was restricted to randomized comparative trials; no single-arm or non-comparative cohort studies were used to estimate relative treatment effects. This restriction reduces concern about pooling randomized and observational designs, but it also limits the evidence base. The included trials were judged to have at least some concerns in several risk-of-bias domains, and a structured qualitative certainty assessment indicated low certainty for all outcomes because of risk of bias, indirectness, imprecision, heterogeneity, and network sparsity in Supplementary Table S1-6.

Third, baseline patient characteristics, disease burden, prior treatments, concomitant medications, and follow-up duration varied across studies. In particular, glucocorticoids were routinely used in chemotherapy regimens, and the ARPI/ADT background was not entirely consistent across endocrine-based combination regimens. These factors may have influenced the occurrence and presentation of adverse events, thereby making it difficult to attribute toxicities specifically to ICIs rather than to concomitant therapies or background treatments. In addition, the definitions and attribution criteria for adverse events were not entirely consistent across the original studies. Some studies reported treatment-related adverse events, whereas others reported overall adverse events; the original adverse-event definitions, grading systems, attribution, safety populations, and follow-up durations are summarized in Supplementary Tables S1-4. Sensitivity analysis excluding studies with clearly incompatible outcome definitions was not feasible because this would have further reduced the already sparse network. It should also be noted that serious and fatal adverse events are not mutually exclusive categories; serious adverse events often include death as a subcategory. We extracted these outcomes as reported, and double-counting or definitional inconsistency may affect their independence. This heterogeneity should be emphasized as a limitation of the pooled estimates.

Fourth, for low-frequency outcomes, such as serious adverse events and fatal adverse events, due to adverse events, the limited number of included studies may have reduced the precision of the effect estimates and weakened the stability and interpretability of the SUCRA rankings. Several odds ratios had very wide 95% confidence intervals, consistent with rare-event and small-sample instability. Event counts and participants per arm for key comparisons are provided in Supplementary Tables S1-5, and these counts should be considered when interpreting statistical significance. Comparison-adjusted funnel plots also had limited ability to detect small-study effects or publication bias because of the small number of studies per outcome.

Fifth, future research could be strengthened in several ways. First, more head-to-head randomized controlled trials are needed, particularly prospective studies directly comparing different ICI-based combination strategies, to enhance the reliability of comparative results. Second, larger sample sizes and more complete data accumulation for key safety outcomes are needed, especially for low-frequency but clinically important endpoints such as serious adverse events and fatal adverse events. In addition, standardized definitions, grading, and attribution criteria for adverse events are necessary to improve comparability across studies and the reliability of pooled analyses. Third, patient stratification based on disease stage, prior treatment background, and biomarker characteristics should be further optimized to better identify populations most likely to benefit. This may facilitate the transition of immunotherapy for metastatic prostate cancer from empiric combination strategies toward precision treatment.

## 5 Conclusion

This systematic review and network meta-analysis provides an exploratory comparison of the safety profiles of different immune checkpoint inhibitor-containing treatment strategies in metastatic prostate cancer, based on 10 randomized comparative trials involving 8,197 participants. The findings suggest that safety profiles varied across regimens. In exploratory SUCRA analyses, PD-1 plus chemotherapy ranked relatively favorably across multiple safety outcomes, including any-grade adverse events, grade ≥3 adverse events, serious adverse events, fatal adverse events, and any-cause adverse events, whereas CTLA-4-containing regimens and PD-(L)1 plus endocrine therapy showed less favorable safety signals, particularly for serious and fatal adverse events. Organ-specific toxicity patterns also differed across regimens. However, because the evidence was based largely on indirect comparisons, sparse networks, and probabilistic rankings, and because several estimates were imprecise, these findings should be interpreted as hypothesis-generating rather than definitive evidence for clinical decision-making. No regimen should be regarded as definitively “safest” on the basis of these exploratory rankings alone. Further high-quality head-to-head comparative studies are needed to better define the risk–benefit profiles of ICI-based combination strategies and to optimize patient selection and safety risk stratification in metastatic prostate cancer.

## Data Availability

The original contributions presented in the study are included in the article/Supplementary Material, further inquiries can be directed to the corresponding author.
