# Supplementary material for: Adverse event profiles of immune checkpoint inhibitor-based strategies for metastatic prostate cancer: a systematic review and network meta-analysis
Source: Front Med (Lausanne). 2026 Oct 30;13:1904711. doi: 10.3389/fmed.2026.1904711 (PMC13759361; doi:10.3389/fmed.2026.1904711)
Supplement: Supplementary file 1 [file Datasheet1.pdf]

SUPPLEMENT

Adverse Event Profiles of Immune Checkpoint Inhibitor-Based Strategies for Metastatic Prostate Cancer: A Systematic Review and Network Meta-Analysis

Table of contents

Supplementary tables

Supplementary Table S1（1-7）. Basic information of all the papersNote.
Supplementary Table S2. Relative effects of different interventions on any-grade adverse events. Supplementary.
Table S3. Relative effects of different interventions on grade ≥3 adverse events.
Supplementary Table S4. Relative effects of different interventions on serious adverse events.
Supplementary Table S5. Relative effects of different interventions on fatal adverse events.
Supplementary Table S6. Relative effects of Any-cause adverse events.
Supplementary Table S7. Relative effects of different interventions on diarrhea.
Supplementary Table S8. Relative effects of different interventions on rash.
Supplementary Table S9. Relative effects of different interventions on Hypothyroidism.
Supplementary Table S10. Relative effects of different interventions on Fatigue.

Supplementary figures

Figure S1 Risk of bias assessment included in the study.
Figure S2 The evidence network of all papers on any grade of diarrhea as an adverse event.
Figure S3. The SUCRA values of each dose modality for diarrhea.
Figure S4. Comparison-adjusted funnel plot for diarrhea.
Figure S5 The evidence network of all papers on any grade of rash as an adverse event.
Figure S6. The SUCRA values of each dose modality for rash.
Figure S7. Comparison-adjusted funnel plot for rash.
Figure S8 The evidence network of all papers on any grade of Hypothyroidism as an adverse event.
Figure S9. The SUCRA values of each dose modality for Hypothyroidism.
Figure S10. Comparison-adjusted funnel plot for Hypothyroidism.
Figure S11 The evidence network of all papers on any grade of Fatigue as an adverse event.
Figure S12. The SUCRA values of each dose modality for Fatigue.
Figure S13. Comparison-adjusted funnel plot for Fatigue.

Figure S14. Meta analysis of ICI plus endocrine therapy versus control for any-grade adverse events.
Figure S15. Leave-one-out sensitivity analysis of the endocrine therapy subgroup for any-grade adverse events
Figure S16. Meta analysis of ICI plus endocrine therapy versus control for grade ≥3 adverse events.
Figure S17. Leave-one-out sensitivity analysis of the endocrine therapy subgroup for grade ≥3 adverse events.
Figure S18. Meta analysis of ICI plus endocrine therapy versus control for serious adverse events.
Figure S19. Leave-one-out sensitivity analysis of the endocrine therapy subgroup for serious adverse events.
Figure S20. Meta analysis of ICI plus endocrine therapy versus control for fatal adverse events.
Figure S21. Leave-one-out sensitivity analysis of the endocrine therapy subgroup for fatal adverse events.
Figure S22. Meta analysis of ICI plus endocrine therapy versus control for any-cause adverse events.
Figure S23. Leave-one-out sensitivity analysis of the endocrine therapy subgroup for any-cause adverse events.
Figure S24. Funnel plot with pseudo 95% confidence limits for the endocrine therapy subgroup for any-grade adverse events.
Figure S25. Funnel plot with pseudo 95% confidence limits for the endocrine therapy subgroup for grade ≥3 adverse events.
Figure S26. Funnel plot with pseudo 95% confidence limits for the endocrine therapy subgroup for serious adverse events.
Figure S27. Funnel plot with pseudo 95% confidence limits for the endocrine therapy subgroup for fatal adverse events.
Figure S28. Funnel plot with pseudo 95% confidence limits for the endocrine therapy subgroup for any-cause adverse events.

Appendix

Appendix S1. Search strategy

Supplementary Table S1-1 Basic information of all the papersNote-characteristics of included studies

**Author (year)**	**Region**	**Phase**	**Sample size (n)**		**Median age (years)**		**Median PSA level (ng/mL)**		**Median duration of treatment (month)**		**Intervention characteristics**		**Outcomes**
			**T**	**C**	**T**	**C**	**T**	**C**	**T**	**C**	**Treatment**	**Control**	
Beer et al. (2016)	International multicenter	Phase III	399	199	70	69	41.2	49.5	NR	NR	CTLA-4	Conventional Therapy	①③④⑤⑥⑦⑨
Gratzke et al. (2025)	International multicenter	Phase III	626	625	68	68	6.0	6.7	17.6	18.4	PD-(L)1 + Endocrine	Conventional Therapy	①②③④⑤⑥⑦⑧⑨
Graff et al. (2025)	International multicenter	Phase III	621	623	71	70	24.4	22.0	8.3	8.1	PD-(L)1 + Endocrine	Conventional Therapy	①②③④⑥⑦⑧⑨
Petrylak et al. (2025)	International multicenter	Phase III	515	515	71	71	30.4	28.7	7.8	8.1	PD-1 + Chemotherapy	Conventional Therapy	①②③④⑤⑥⑨
Antonarakis et al. (2023)	International multicenter	Phase III	529	264	71	69	52.9	42.6	5.0	4.1	PD-(L)1 + Targeted	Conventional Therapy	①②④⑤⑥⑦⑧⑨
Agarwal et al. (2025)	International multicenter	Phase III	289	286	71	71	31.4	26.9	6.3	4.2	PD-(L)1 + Targeted	Conventional Therapy	②③④
Fizazi et al. (2020)	International multicenter	Phase III	399	400	69.0	67.5	138.5	176.5	NR	NR	CTLA-4 + RT	Conventional Therapy	⑤
Powles et al. (2022)	International multicenter	Phase III	379	380	NR	NR	NR	NR	3.5	4.1	PD-(L)1 + Endocrine	Conventional Therapy	①③④⑤
Fizazi et al. (2026)	International multicenter	Phase III	510	510	69	70	33.9	29.9	7.6	7.8	PD-1 + Chemotherapy	Conventional Therapy	①③④

Aparicio et al. (2024)	USA	Phase II	64	64	68	66	11.3	14.1	NR	NR	CTLA-4 +Endocrine	Conventional Therapy	①②⑥⑧⑨

Notes：①Any-grade adverse events, ②Grade ≥3 adverse events, ③Serious adverse events, ④Fatal adverse events, ⑤Any-cause adverse events, ⑥Diarrhea, ⑦Rash, ⑧Hypothyroidism, ⑨Fatigue.

Supplementary Table S1-2 Basic information of all the papersNote-treatment node definitions and regimens

**Node**	**Trial / Study**	**Experimental regimen**	**Background therapy**	**Disease setting**	**Control / conventional therapy**	**Design**
CTLA-4	Beer et al. (2016)	Ipilimumab 10 mg/kg IV q3w for up to 4 doses; maintenance q3mo for nonprogressing patients	Ongoing ADT / castration	mCRPC, chemotherapy-naive, asymptomatic or minimally symptomatic, no visceral metastases	Placebo	Randomized, double-blind, phase III
PD-(L)1 + Endocrine	Gratzke et al. (2025)	Pembrolizumab 200 mg IV q3w for ≤35 cycles + enzalutamide 160 mg PO daily + ADT	ADT + enzalutamide	mHSPC, NHA-naive	Placebo + enzalutamide + ADT	Randomized, double-blind, phase III
PD-(L)1 + Endocrine	Graff et al. (2025)	Pembrolizumab 200 mg IV q3w for ≤35 cycles + enzalutamide 160 mg PO daily	Enzalutamide	Chemotherapy-naive mCRPC; prior abiraterone permitted	Placebo + enzalutamide	Randomized, double-blind, phase III
PD-(L)1 + Endocrine	Powles et al. (2022)	Atezolizumab 1200 mg IV q3w + enzalutamide 160 mg PO daily	Enzalutamide	mCRPC progressed on abiraterone; taxane ineligible, refused, or progressed	Enzalutamide alone	Randomized, open-label, phase III
PD-1 + Chemotherapy	Petrylak et al. (2025)	Pembrolizumab 200 mg IV q3w for ≤35 cycles + docetaxel 75 mg/m^2 IV q3w for ≤10 cycles + prednisone 5 mg BID	Docetaxel + prednisone	ARPI-pretreated, chemotherapy-naive mCRPC	Placebo + docetaxel + prednisone	Randomized, double-blind, phase III

PD-1 + Chemotherapy	Fizazi et al. (2026)	Nivolumab 360 mg IV q3w + docetaxel 75 mg/m² IV q3w for up to 10 doses + prednisone 5 mg BID; then nivolumab 480 mg IV q4w maintenance	Docetaxel + prednisone	ARPI-pretreated, chemotherapy-naive mCRPC	Placebo + docetaxel + prednisone	Randomized, double-blind, phase III
PD-(L)1 + Targeted	Antonarakis et al. (2023)	Pembrolizumab 200 mg IV q3w for ≤35 cycles + olaparib 300 mg PO BID	Olaparib	Biomarker-unselected mCRPC; progressed on or after abiraterone or enzalutamide (not both) and docetaxel	NHA switch: abiraterone 1000 mg qd + prednisone 5 mg BID if prior enzalutamide; or enzalutamide 160 mg qd if prior abiraterone	Randomized, open-label, phase III
PD-(L)1 + Targeted	Agarwal et al. (2025)	Cabozantinib 40 mg PO daily + atezolizumab 1200 mg IV q3w	Cabozantinib + atezolizumab	mCRPC with measurable extrapelvic soft-tissue metastases; progressed on one prior ARPI	ARPI switch: abiraterone 1000 mg qd + prednisone 5 mg BID, or enzalutamide 160 mg qd	Randomized, open-label, phase III
CTLA-4 + RT	Fizazi et al. (2020)	Bone-directed RT 8 Gy single fraction + ipilimumab 10 mg/kg IV q3w for up to 4 doses; maintenance q3mo	RT + ongoing ADT / castration	Post-docetaxel mCRPC with at least one bone metastasis	RT + placebo	Randomized, double-blind, phase III
CTLA-4 + Endocrine	Aparicio et al. (2024)	Ipilimumab 3 mg/kg IV for up to 4 cycles + AAPA: abiraterone 1000 mg qd + prednisone 5 mg BID + apalutamide 240 mg qd	AAPA	mCRPC; after 8-week AAPA lead-in with satisfactory status: ≥50% PSA decline and ≤5 CTC/7.5 mL	AAPA alone	Randomized, open-label, single-center, phase II

Supplementary Table S1-3 Basic information of all the papersNote-potential effect modifiers and heterogeneity

**Node / comparison**	**Disease state**	**Line of therapy**	**Prior chemotherapy**	**Background androgen-axis therapy**	**Median age**	**ECOG PS**	**Treatment duration**	**Safety follow-up**
CTLA-4 (Beer et al. 2016)	mCRPC, chemotherapy-naive	First-line mCRPC	No	Ongoing ADT/castration	69–70 y	0–1	Up to 4 doses + maintenance	Until 90 d after last dose
PD-(L)1 + Endocrine (Gratzke et al. 2025)	mHSPC, NHA-naive	First-line mHSPC	No	ADT + enzalutamide	68 y	0–1	Median 17.6 mo	Until 90 d after last dose
PD-(L)1 + Endocrine (Graff et al. 2025)	Chemotherapy-naive mCRPC	First-line mCRPC	No	Enzalutamide	70–71 y	0–1	Median 8.3 mo	Until 30–90 d after last dose
PD-(L)1 + Endocrine (Powles et al. 2022)	mCRPC, post-abiraterone	Second-line mCRPC	Taxane ineligible/refused/progressed	Enzalutamide	69–70 y	0–1	Median 3.5–4.5 mo	Until 30–90 d after last dose
PD-1 + Chemotherapy (Petrylak et al2025)	ARPI-pretreated, chemotherapy-naive mCRPC	Post-ARPI, first chemo	No	Docetaxel + prednisone	71 y	0–1	Median 7.8–8.1 mo	Until 30–90 d after last dose
PD-1 + Chemotherapy (Fizazi et al. 2026)	ARPI-pretreated, chemotherapy-naive mCRPC	Post-ARPI, first chemo	No	Docetaxel + prednisone	70 y	0–1	Median 7.6–7.8 mo	Until 30–100 d after last dose
PD-(L)1 + Targeted (Antonarakis et al. 2023)	Biomarker-unselected mCRPC, post-abiraterone/enzalutamide and docetaxel	Third-line or later	Yes	Olaparib	69–71 y	0–1	Median 5.0 mo	Until 30–90 d after last dose

PD-(L)1 + Targeted (Agarwal et al. 2025)	mCRPC with extrapelvic soft-tissue metastases, post-ARPI	Post-ARPI	Docetaxel allowed in mHSPC	Cabozantinib + atezolizumab	71–72 y	0–1	Median 6.3 mo	Until 30–100 d after last dose
CTLA-4 + RT (Fizazi et al. 2020)	Post-docetaxel mCRPC with bone metastases	Post-docetaxel	Yes	RT + ADT/castration	67.5–69 y	0–1	Up to 4 doses + maintenance	Until 70 d after last dose
CTLA-4 + Endocrine (Aparicio et al. 2024)	mCRPC after 8-week AAPA with satisfactory status	First-line mCRPC	No	AAPA	NA	0–2	Up to 4 ipilimumab cycles + AAPA	Until progression

Supplementary Table S1-4 Basic information of all the papersNote-adverse event definitions and safety follow-up

Study	Original AE definition	Grading system	Treatment-related vs all-cause	Safety-analysis population	Treatment duration / safety follow-up
Beer et al. (2016)	Treatment-related AEs and all-cause AEs	NCI CTCAE v3.0	Both reported	As-treated: 399 ipilimumab, 199 placebo	Treatment until progression/toxicity; safety follow-up until 90 days after last dose
Gratzke et al. (2025)	Any-cause AEs; treatment-related AEs; grade ≥3 AEs; SAEs	Not explicitly stated	Both reported	As-treated: 625 per arm	Median 17.6 months; safety follow-up 30–90 days
Graff et al. (2025)	Treatment-related AEs; any-grade, any-cause AEs	NCI CTCAE v4.0	Both reported	As-treated: 615 pembrolizumab+enzalutamide; 620 placebo+enzalutamide	Median 8.3 months; safety follow-up 30 days (90 days for SAEs)
Powles et al. (2022)	AEs; treatment-related AEs; grade 3/4 AEs; SAEs	NCI CTCAE v4.0	Both reported	Safety population: 374 atezolizumab+enzalutamide; 376 enzalutamide	Median 3.5–4.5 months; follow-up not explicitly stated

Petrylak et al. (2025)	Treatment-related AEs; all-cause AEs; SAEs	Not explicitly stated	Both reported	As-treated: 514 per arm	Median 7.8–8.1 months; safety follow-up 30 days
Antonarakis et al. (2023)	Treatment-related AEs; any-grade, any-cause AEs	Not explicitly stated	Both reported	As-treated: 526 pembrolizumab+olaparib; 256 NHA	Median 5.0 months; safety follow-up 30 days
Fizazi et al. (2020)	On-study AEs; immune-related AEs	NCI CTCAE v3.0	All-cause on-study AEs; irAEs reported separately	Treated: 393 ipilimumab; 396 placebo	Up to 4 doses + maintenance; safety follow-up 70 days
Agarwal et al. (2025)	Treatment-emergent AEs (TEAEs); treatment-related SAEs	NCI CTCAE v5.0	Both reported	Safety population: 284 per arm	Median 6.3 months; safety follow-up 30 and 100 days
Fizazi et al. (2026)	Treatment-related AEs; immune-mediated AEs	NCI CTCAE v5.0	Primarily treatment-related; immune-mediated reported separately	Treated: 510 per arm	Median 7.6–7.8 months; safety follow-up 30 days (100 days for immune-mediated)
Aparicio et al. (2024)	Treatment-related AEs (≥10% in any module)	NCI CTCAE v4.0	Treatment-related only	Treated: 64 per arm	Treatment until progression; follow-up not explicitly stated

Supplementary Table S1-5 Basic information of all the papersNote-event counts and sample sizes by direct comparison

**Comparison**	**Studies, n**	**Experimental participants, n**	**Experimental events, n**	**Control participants, n**	**Control events, n**
CTLA-4 vs CT	1	399	325	199	98
PD-(L)1 + Endocrine vs CT	3	1614	1320	1621	993
PD-1 + Chemotherapy vs CT	2	1024	956	1024	959
PD-(L)1 + Targeted vs CT	1	526	464	256	130

CTLA-4 + Endocrine vs CT	1	64	64	64	58
PD-(L)1 + Endocrine vs CT	2	1240	453	1245	154
PD-1 + Chemotherapy vs CT	1	514	222	514	188
PD-(L)1 + Targeted vs CT	2	810	295	540	46
CTLA-4 + Endocrine vs CT	1	64	37	64	19
CTLA-4 vs CT	1	399	135	199	7
PD-(L)1 + Endocrine vs CT	3	1614	255	1621	48
PD-1 + Chemotherapy vs CT	2	1024	211	1024	155
PD-(L)1 + Targeted vs CT	1	284	45	284	11
CTLA-4 vs CT	1	399	9	199	0
PD-(L)1 + Endocrine vs CT	3	1614	16	1621	2
PD-1 + Chemotherapy vs CT	2	1024	14	1024	8
PD-(L)1 + Targeted vs CT	2	810	4	540	0
CTLA-4 vs CT	1	399	381	199	182
PD-(L)1 + Endocrine vs CT	2	999	979	1001	940
PD-1 + Chemotherapy vs CT	1	514	508	514	505
PD-(L)1 + Targeted vs CT	1	526	516	256	237
CTLA-4 + RT vs CT	1	393	385	396	365

CTLA-4 vs CT	1	399	171	199	27
PD-(L)1 + Endocrine vs CT	2	1240	133	1245	70
PD-1 + Chemotherapy vs CT	1	514	178	514	158
PD-(L)1 + Targeted vs CT	1	526	66	256	6
CTLA-4 + Endocrine vs CT	1	64	21	64	3
CTLA-4 vs CT	1	399	132	199	15
PD-(L)1 + Endocrine vs CT	2	1240	240	1245	57
PD-(L)1 + Targeted vs CT	1	526	27	256	0
PD-(L)1 + Endocrine vs CT	2	1240	131	1245	38
PD-(L)1 + Targeted vs CT	1	526	47	256	2
CTLA-4 + Endocrine vs CT	1	64	15	64	11
CTLA-4 vs CT	1	399	97	199	28
PD-(L)1 + Endocrine vs CT	2	1240	280	1245	247
PD-1 + Chemotherapy vs CT	1	514	159	514	159
PD-(L)1 + Targeted vs CT	1	526	135	256	42
CTLA-4 + Endocrine vs CT	1	64	31	64	19

Supplementary Table S1-6 Basic information of all the papersNote-qualitative certainty assessment

**Outcome**	**Risk of bias**	**Indirectness**	**Imprecision**	**Heterogeneity**	**Network sparsity**	**Overall certainty**

Any-grade adverse events	Most RCTs had some concerns, mainly in randomization, outcome measurement, and reporting	Serious: mixed disease states, prior therapy, background therapy, and AE definitions	Serious: Sparse network; most nodes included only 1–3 RCTs; predominantly indirect evidence	Not reported for all nodes	Very serious: star-shaped, no closed loops	Low
Grade ≥3 adverse events	Most RCTs had some concerns, mainly in randomization, outcome measurement, and reporting	Serious: mixed disease states, lines of therapy, and background therapies	Serious: Sparse network; most nodes included only 1–2 RCTs; predominantly indirect evidence	Not reported for all nodes	Very serious: star-shaped, no closed loops	Low
Serious adverse events	Most RCTs had some concerns, mainly in randomization, outcome measurement, and reporting	Serious: mixed AE attribution and safety populations	Serious: Sparse network; most nodes included only 1–3 RCTs; predominantly indirect evidence	Not reported for all nodes	Very serious: star-shaped, no closed loops	Low
Fatal adverse events	Most RCTs had some concerns, mainly in randomization, outcome measurement, and reporting	Serious: mixed definitions of treatment-related death	Serious: Sparse network; most nodes included only 1–3 RCTs; predominantly indirect evidence	Not reported for all nodes	Very serious: star-shaped, no closed loops	Low
Any-cause adverse events	Most RCTs had some concerns, mainly in randomization, outcome measurement, and reporting	Serious: all-cause vs TEAE definitions differ across trials	Serious: Sparse network; most nodes included only 1–2 RCTs; predominantly indirect evidence	Not reported for all nodes	Very serious: star-shaped, no closed loops	Low

Diarrhea	Most RCTs had some concerns, mainly in randomization, outcome measurement, and reporting	Serious: organ-specific attribution and definitions vary	Serious: Sparse network; most nodes included only 1–2 RCTs; predominantly indirect evidence	Not reported for all nodes	Very serious: star-shaped, no closed loops	Low
Rash	Most RCTs had some concerns, mainly in randomization, outcome measurement, and reporting	Serious: rash definitions and attribution differ	Serious: Sparse network; most nodes included only 1–2 RCTs; predominantly indirect evidence	Not reported for all nodes	Very serious: star-shaped, no closed loops	Low
Hypothyroidism	Most RCTs had some concerns, mainly in randomization, outcome measurement, and reporting	Serious: thyroid AE definitions and monitoring differ	Serious: Sparse network; most nodes included only 1–2 RCTs; predominantly indirect evidence	Not reported for all nodes	Very serious: star-shaped, no closed loops	Low
Fatigue	Most RCTs had some concerns, mainly in randomization, outcome measurement, and reporting	Serious: fatigue vs asthenia definitions differ	Serious: Sparse network; all nodes included only 1 RCTs; predominantly indirect evidence	Not reported for all nodes	Very serious: star-shaped, no closed loops	Low

Supplementary Table S1-7 Between-study heterogeneity parameters for each outcome in the random-effects network meta-analysis

Outcome	Trials per node	I^2	τ^2	Interpretation
Any-grade adverse events	Most nodes 1–3 trials	Not estimable	0.04	Common τ^2 estimated across the network; sparse data and few trials per comparison limit precision.
Grade ≥3 adverse events	Most nodes 1–2 trials	Not estimable	0	Common τ^2 estimated across the network; sparse data and few trials per comparison limit precision.

Serious adverse events	Most nodes 1–3 trials	Not estimable	0	Common τ^2 estimated across the network; sparse data and few trials per comparison limit precision.
Fatal adverse events	Most nodes 1–3 trials	Not estimable	1.52	Common τ^2 estimated across the network; sparse data and few trials per comparison limit precision.
Any-cause adverse events	Most nodes 1–2 trials	Not estimable	0.02	Common τ^2 estimated across the network; sparse data and few trials per comparison limit precision.
Diarrhea	Most nodes 1–2 trials	Not estimable	0.11	Common τ^2 estimated across the network; sparse data and few trials per comparison limit precision.
Rash	Most nodes 1–2 trials	Not estimable	0	Common τ^2 estimated across the network; sparse data and few trials per comparison limit precision.
Hypothyroidism	Most nodes 1–2 trials	Not estimable	0	Common τ^2 estimated across the network; sparse data and few trials per comparison limit precision.
Fatigue	All nodes 1 trials	Not estimable	0	Common τ^2 estimated across the network; sparse data and few trials per comparison limit precision.

Note: τ^2, common network-wide between-study variance estimated under the random-effects consistency NMA. I^2 is not a standard NMA output and was not reliably estimable because the networks were sparse; therefore, τ^2 and qualitative assessment were used to describe heterogeneity.

Supplementary Table S2. Relative effects of different interventions on any-grade adverse events.

PD-1 + Chemotherapy					
0.95 (0.61,1.49)	Conventional therapy				
0.32 (0.19,0.55)	0.34 (0.26,0.45)	PD-(L)1 + Endocrine			
0.21 (0.10,0.43)	0.22 (0.13,0.38)	0.65 (0.35,1.20)	CTLA-4		
0.13 (0.07,0.26)	0.14 (0.08,0.23)	0.41 (0.22,0.74)	0.62 (0.29,1.34)	PD-(L)1 + Targeted	
0.07 (0.00,1.28)	0.07 (0.00,1.30)	0.21 (0.01,3.88)	0.32 (0.02,6.18)	0.51 (0.03,9.89)	CTLA-4 + Endocrine

Note: Effect estimates are presented as odds ratios (ORs) with 95% confidence intervals (CIs). Table cells are color-coded to facilitate interpretation. Interventions are listed in the purple columns. Statistically significant results (95% CI excluding 1) are highlighted in yellow, whereas non-significant results (95% CI including 1) are shown in light blue.

Supplementary Table S3. Relative effects of different interventions on grade ≥3 adverse events.

Conventional therapy				
0.76 (0.59,0.97)	PD-1 + Chemotherapy			
0.31 (0.15,0.64)	0.41 (0.19,0.88)	CTLA-4 + Endocrine		
0.24 (0.20,0.30)	0.32 (0.23,0.44)	0.79 (0.37,1.69)	PD-(L)1 + Endocrine	
0.16 (0.11,0.22)	0.21 (0.14,0.32)	0.52 (0.23,1.15)	0.65 (0.44,0.97)	PD-(L)1 + Targeted

Note: Effect estimates are presented as odds ratios (ORs) with 95% confidence intervals (CIs). Table cells are color-coded to facilitate interpretation. Interventions are listed in the purple columns. Statistically significant results (95% CI excluding 1) are highlighted in yellow, whereas non-significant results (95% CI including 1) are shown in light blue.

Supplementary Table S4. Relative effects of different interventions on serious adverse events.

Conventional therapy				
0.69 (0.55,0.86)	PD-1 + Chemotherapy			
0.21 (0.11,0.42)	0.31 (0.15,0.64)	PD-(L)1 + Targeted		
0.16 (0.12,0.23)	0.24 (0.16,0.35)	0.77 (0.36,1.62)	PD-(L)1 + Endocrine	
0.07 (0.03,0.16)	0.10 (0.05,0.23)	0.33 (0.12,0.94)	0.43 (0.19,1.01)	CTLA-4

Note: Effect estimates are presented as odds ratios (ORs) with 95% confidence intervals (CIs). Table cells are color-coded to facilitate interpretation. Interventions are listed in the purple columns. Statistically significant results (95% CI excluding 1) are highlighted in yellow, whereas non-significant results (95% CI including 1) are shown in light blue.

Supplementary Table S5. Relative effects of different interventions on fatal adverse events.

Conventional therapy				
0.64 (0.07,5.47)	PD-1 + Chemotherapy			
0.41 (0.02,7.74)	0.65 (0.02,24.66)	PD-(L)1 + Targeted		
0.15 (0.02,1.05)	0.23 (0.01,4.25)	0.36 (0.01,12.23)	PD-(L)1 + Endocrine	
0.10 (0.00,4.32)	0.16 (0.00,12.01)	0.25 (0.00,28.85)	0.69 (0.01,46.82)	CTLA-4

Note: Effect estimates are presented as odds ratios (ORs) with 95% confidence intervals (CIs). Table cells are color-coded to facilitate interpretation. Interventions are listed in the purple columns. Statistically significant results (95% CI excluding 1) are highlighted in yellow, whereas non-significant results (95% CI including 1) are shown in light blue.

Supplementary Table S6. Relative effects of Any-cause adverse events.

Conventional therapy					
0.66 (0.23,1.95)	PD-1 + Chemotherapy				
0.51 (0.24,1.06)	0.76 (0.21,2.82)	CTLA-4			
0.32 (0.18,0.57)	0.48 (0.14,1.63)	0.63 (0.25,1.60)	PD-(L)1 + Endocrine		
0.24 (0.11,0.57)	0.37 (0.09,1.45)	0.48 (0.16,1.48)	0.77 (0.28,2.13)	CTLA-4 + RT	
0.24 (0.11,0.55)	0.36 (0.09,1.42)	0.48 (0.16,1.45)	0.76 (0.28,2.09)	0.99 (0.30,3.21)	PD-(L)1 + Targeted

Note: Effect estimates are presented as odds ratios (ORs) with 95% confidence intervals (CIs). Table cells are color-coded to facilitate interpretation. Interventions are listed in the purple columns. Statistically significant results (95% CI excluding 1) are highlighted in yellow, whereas non-significant results (95% CI including 1) are shown in light blue.

Supplementary Table S7. Relative effects of different interventions on diarrhea.

Conventional therapy					
0.84 (0.42,1.69)	PD-1 + Chemotherapy				
0.49 (0.28,0.84)	0.58 (0.24,1.42)	PD-(L)1 + Endocrine			
0.21 (0.09,0.46)	0.25 (0.09,0.72)	0.43 (0.16,1.13)	CTLA-4		
0.17 (0.06,0.49)	0.20 (0.06,0.72)	0.34 (0.10,1.15)	0.80 (0.21,3.02)	PD-(L)1 + Targeted	
0.10 (0.02,0.42)	0.12 (0.02,0.59)	0.21 (0.04,0.96)	0.48 (0.09,2.46)	0.60 (0.10,3.58)	CTLA-4 + Endocrine

Note: Effect estimates are presented as odds ratios (ORs) with 95% confidence intervals (CIs). Table cells are color-coded to facilitate interpretation. Interventions are listed in the purple columns. Statistically significant results (95% CI excluding 1) are highlighted in yellow, whereas non-significant results (95% CI including 1) are shown in light blue.

Supplementary Table S8. Relative effects of different interventions on rash.

Conventional therapy			
0.20 (0.15,0.27)	PD-(L)1 + Endocrine		
0.16 (0.09,0.29)	0.82 (0.43,1.56)	CTLA-4	
0.04 (0.00,0.58)	0.18 (0.01,2.96)	0.21 (0.01,3.74)	PD-(L)1 + Targeted

Note: Effect estimates are presented as odds ratios (ORs) with 95% confidence intervals (CIs). Table cells are color-coded to facilitate interpretation. Interventions are listed in the purple columns. Statistically significant results (95% CI excluding 1) are highlighted in yellow, whereas non-significant results (95% CI including 1) are shown in light blue.

Supplementary Table S9. Relative effects of different interventions on Hypothyroidism.

Conventional therapy			
0.68 (0.28,1.62)	CTLA-4 + Endocrine		
0.27 (0.18,0.39)	0.39 (0.15,1.01)	PD-(L)1 + Endocrine	
0.08 (0.02,0.33)	0.12 (0.02,0.63)	0.30 (0.07,1.31)	PD-(L)1 + Targeted

Note: Effect estimates are presented as odds ratios (ORs) with 95% confidence intervals (CIs). Table cells are color-coded to facilitate interpretation. Interventions are listed in the purple columns. Statistically significant results (95% CI excluding 1) are highlighted in yellow, whereas non-significant results (95% CI including 1) are shown in light blue.

Supplementary Table S10. Relative effects of different interventions on Fatigue.

Conventional therapy					
1.00 (0.77,1.30)	PD-1 + Chemotherapy				
0.85 (0.70,1.03)	0.85 (0.61,1.18)	PD-(L)1 + Endocrine			
0.57 (0.39,0.83)	0.57 (0.36,0.91)	0.67 (0.44,1.03)	PD-(L)1 + Targeted		
0.51 (0.32,0.81)	0.51 (0.30,0.87)	0.60 (0.36,0.99)	0.90 (0.49,1.63)	CTLA-4	
0.45 (0.22,0.93)	0.45 (0.21,0.97)	0.53 (0.25,1.12)	0.79 (0.35,1.80)	0.88 (0.37,2.08)	CTLA-4 + Endocrine

Note: Effect estimates are presented as odds ratios (ORs) with 95% confidence intervals (CIs). Table cells are color-coded to facilitate interpretation. Interventions are listed in the purple columns. Statistically significant results (95% CI excluding 1) are highlighted in yellow, whereas non-significant results (95% CI including 1) are shown in light blue.

Figure S1 Risk of bias assessment included in the study.

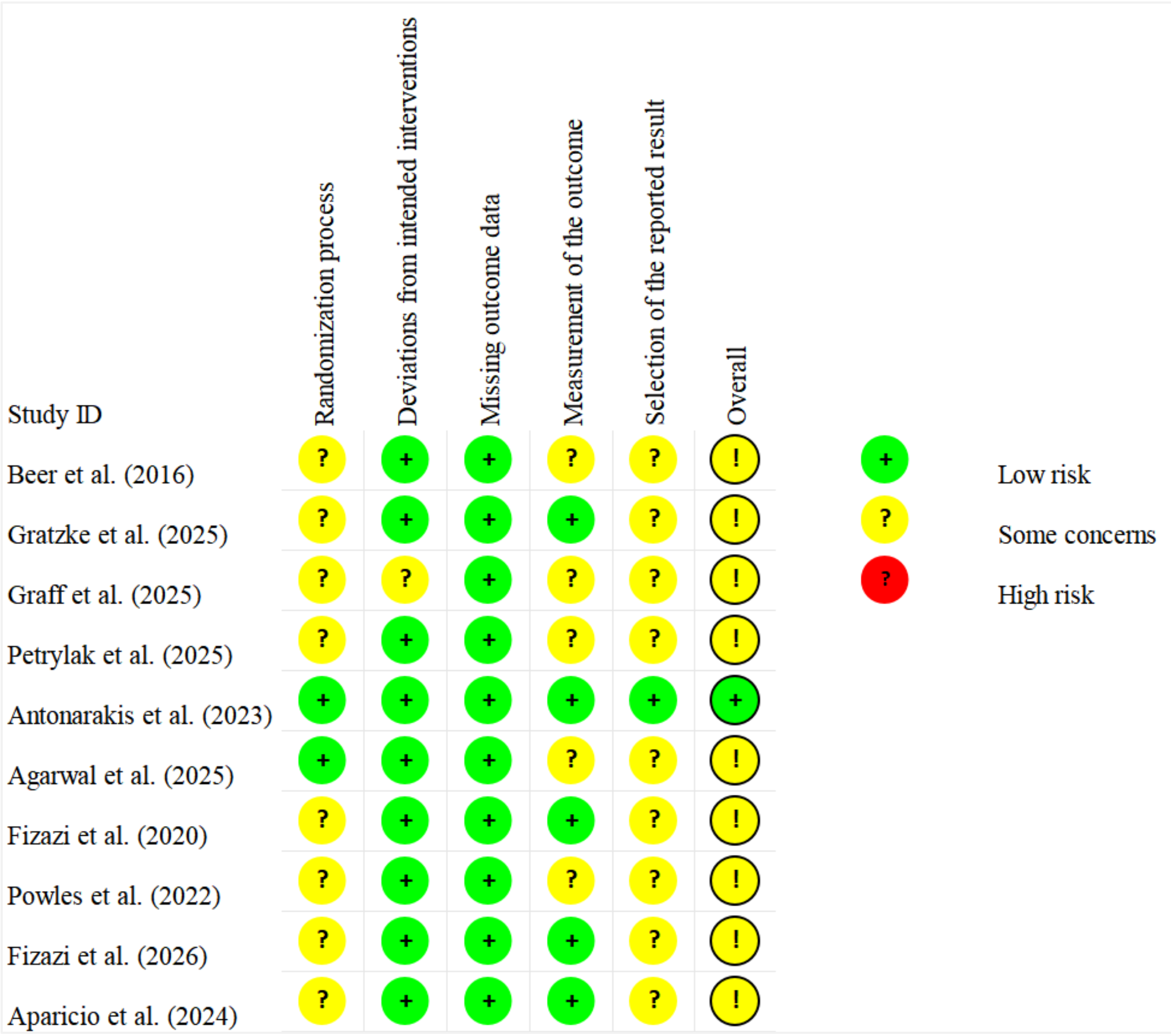

Figure S2 The evidence network of all papers on any grade of diarrhea as an adverse event.

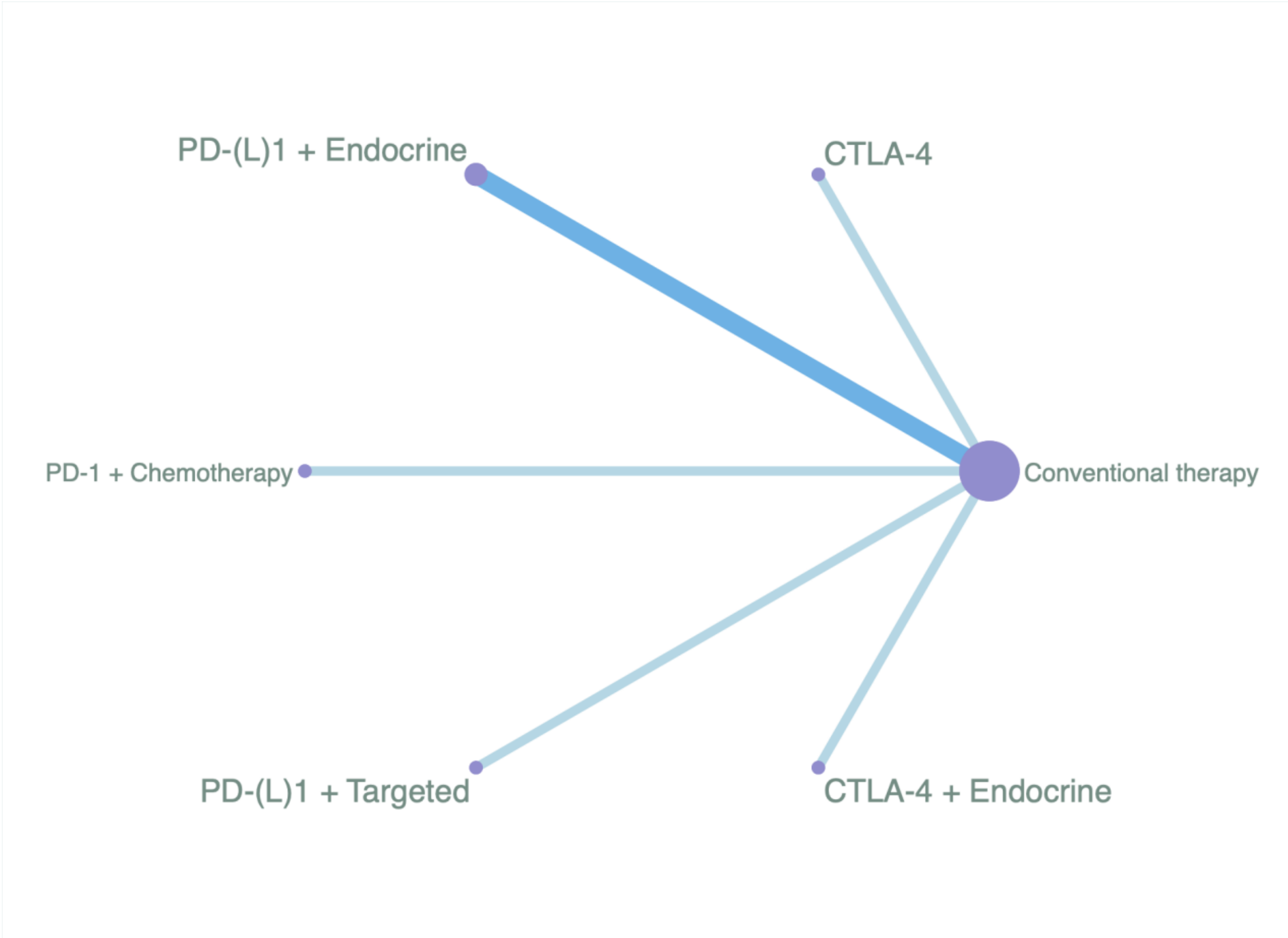

Figure S3. The SUCRA values of each dose modality for diarrhea.

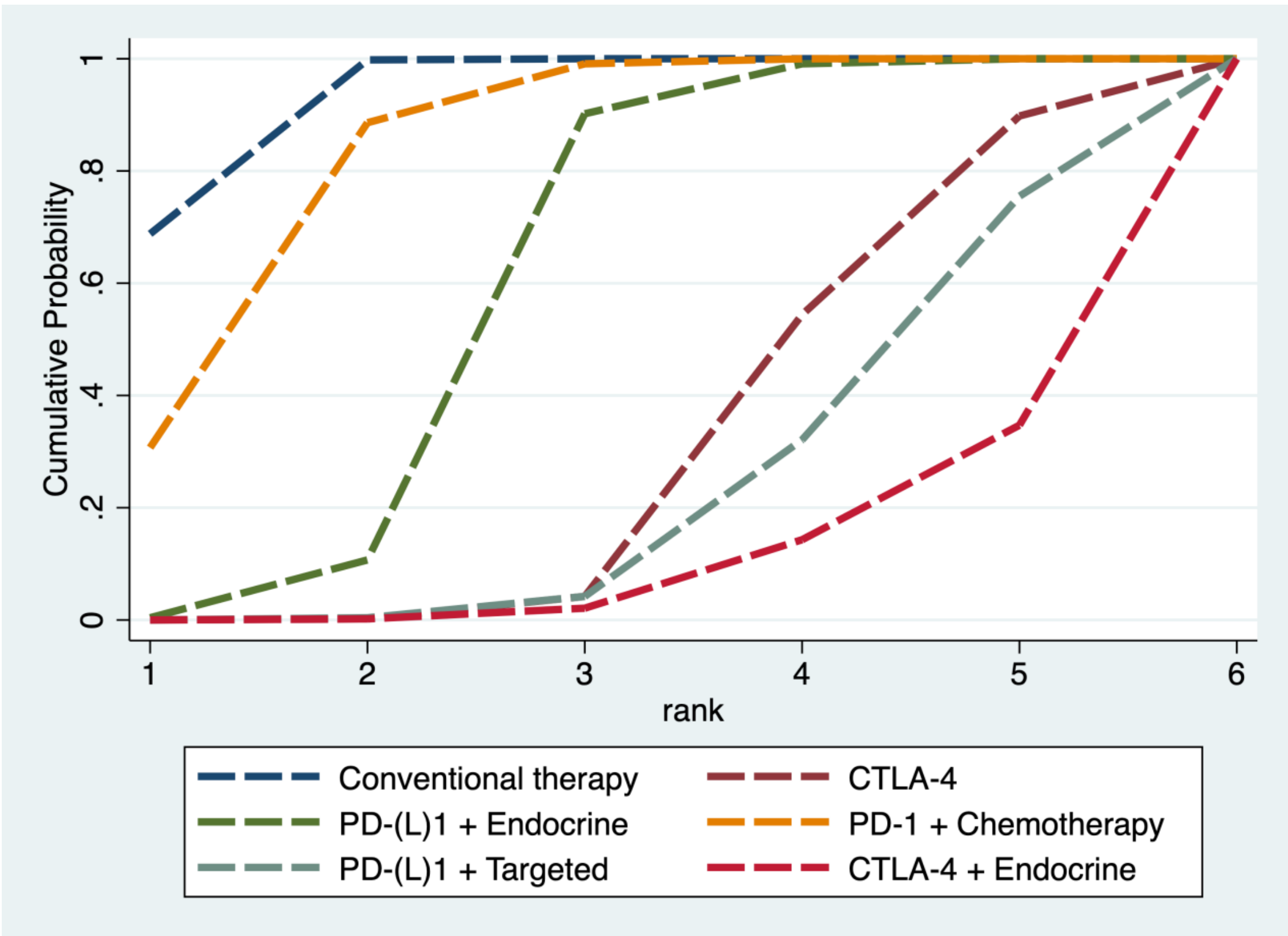

Figure S4. Comparison-adjusted funnel plot for diarrhea.

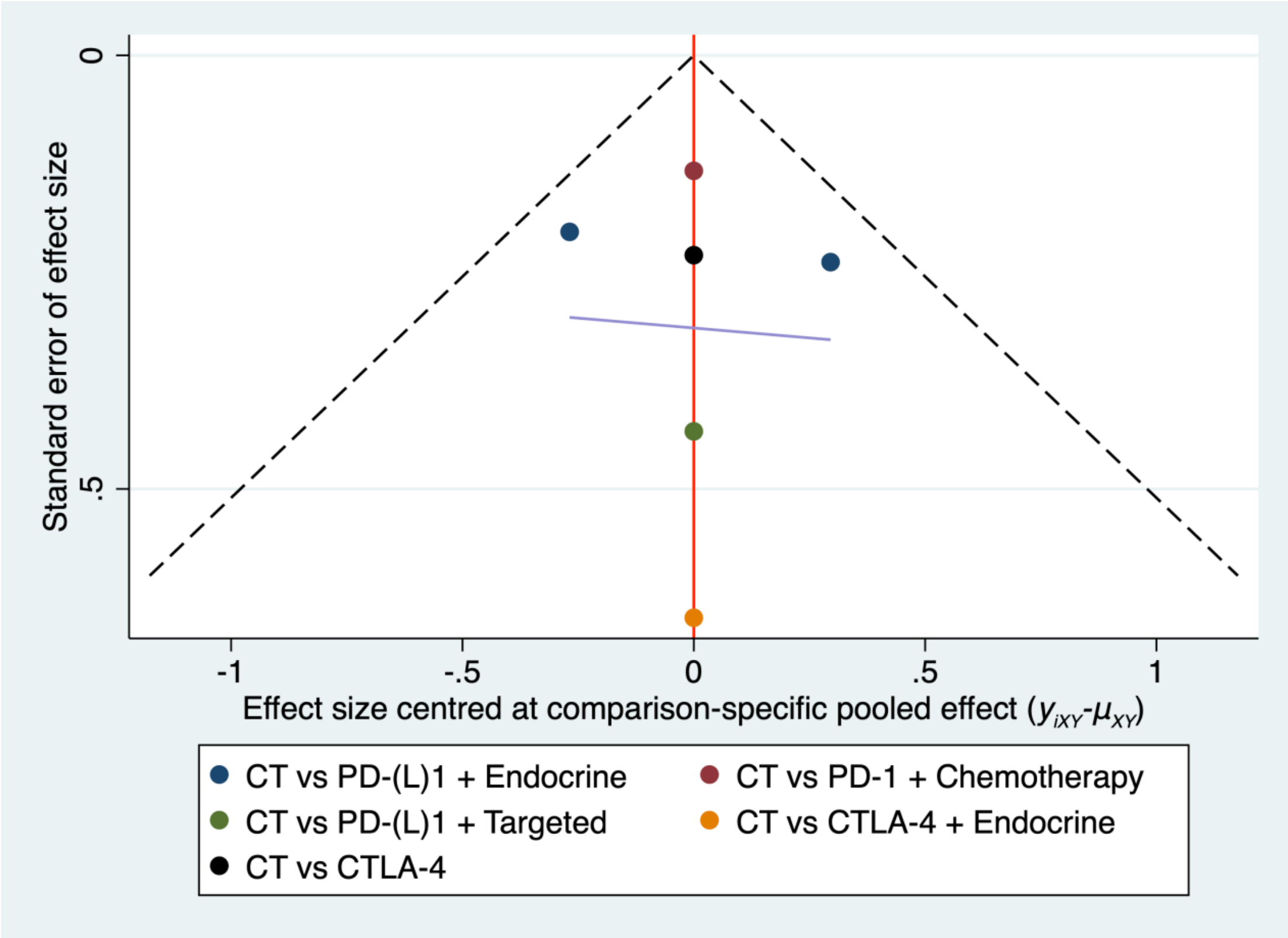

Figure S5 The evidence network of all papers on any grade of rash as an adverse event.

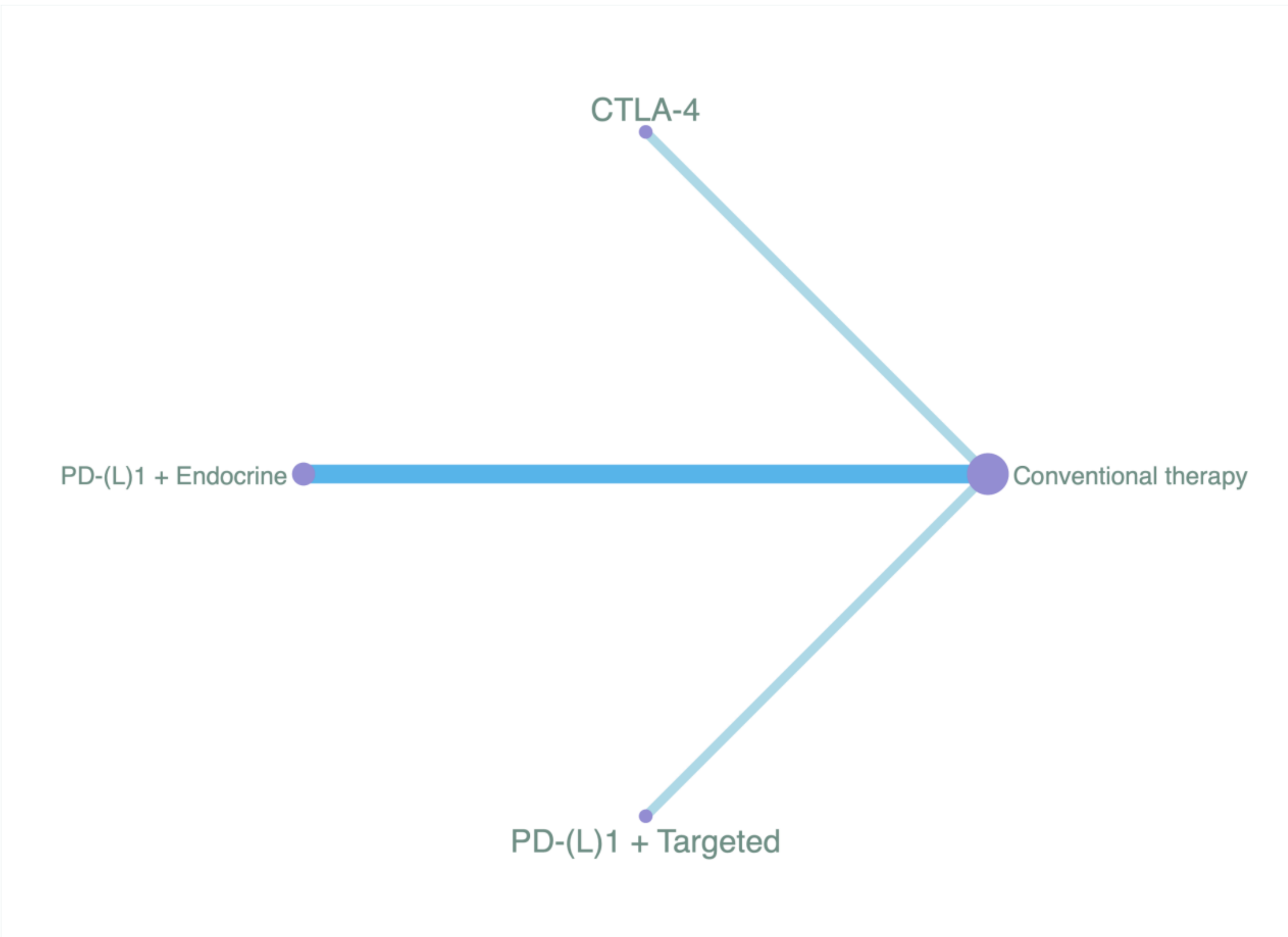

Figure S6. The SUCRA values of each dose modality for rash.

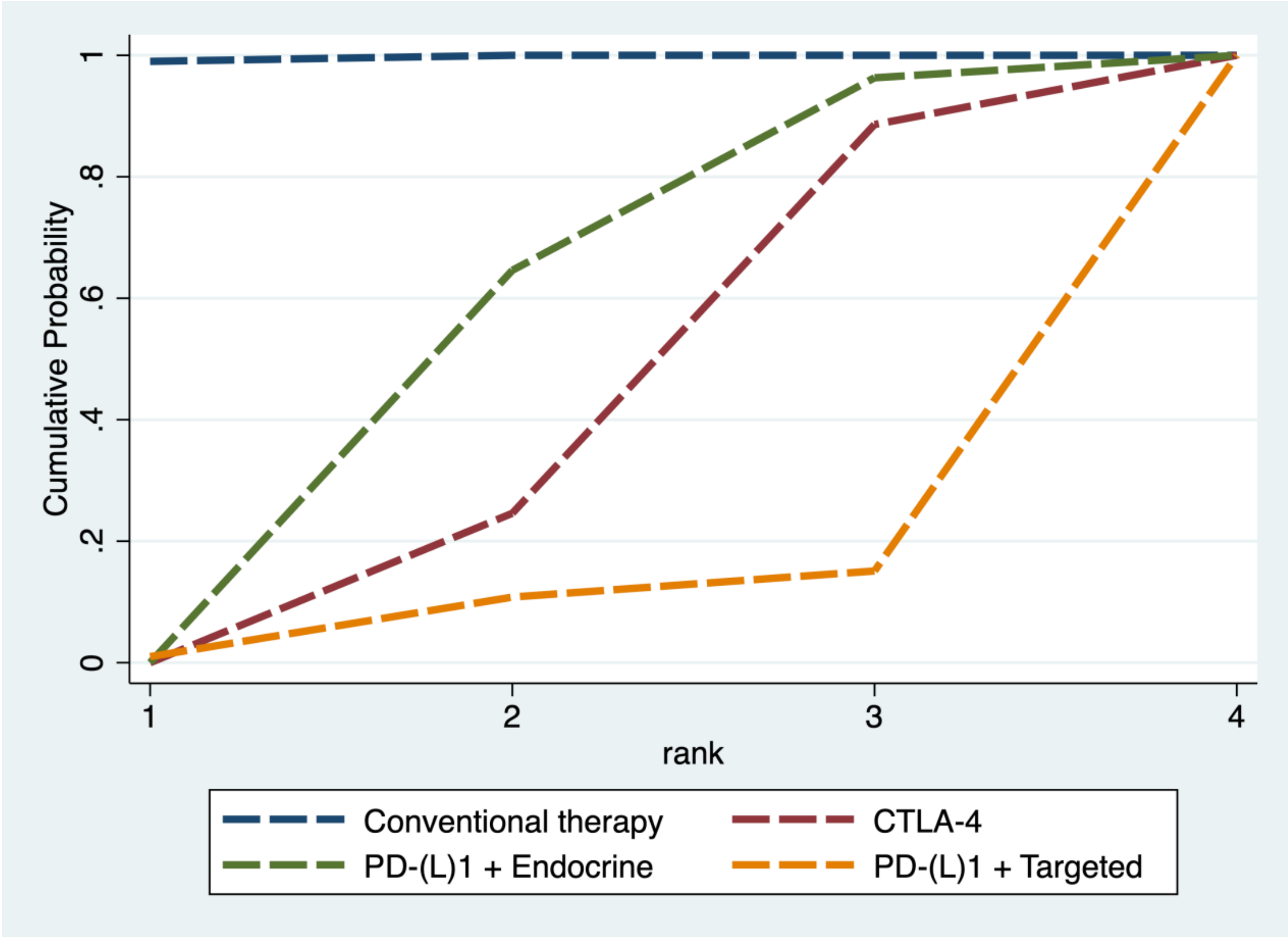

Figure S7. Comparison-adjusted funnel plot for rash.

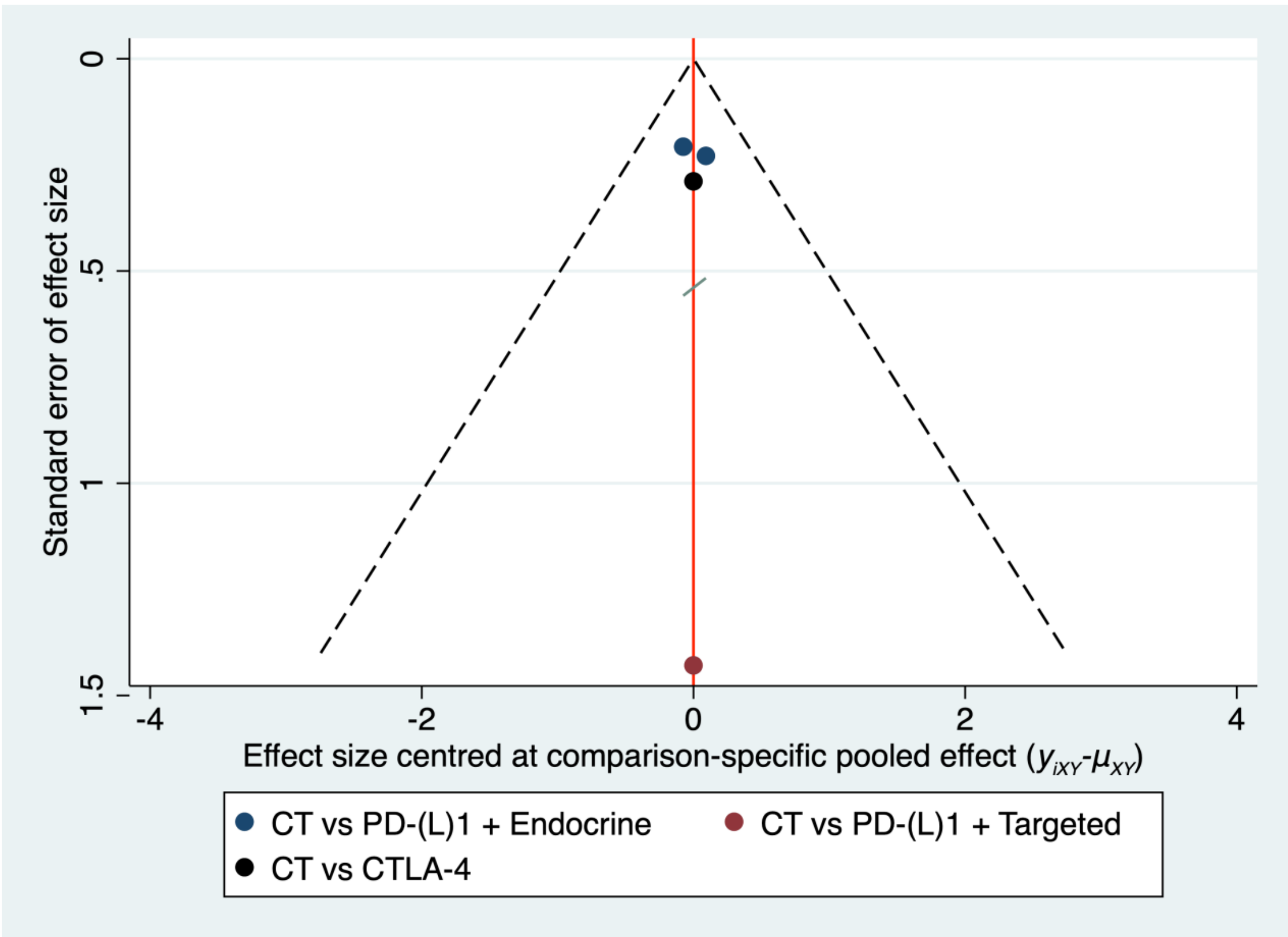

Figure S8 The evidence network of all papers on any grade of Hypothyroidism as an adverse event.

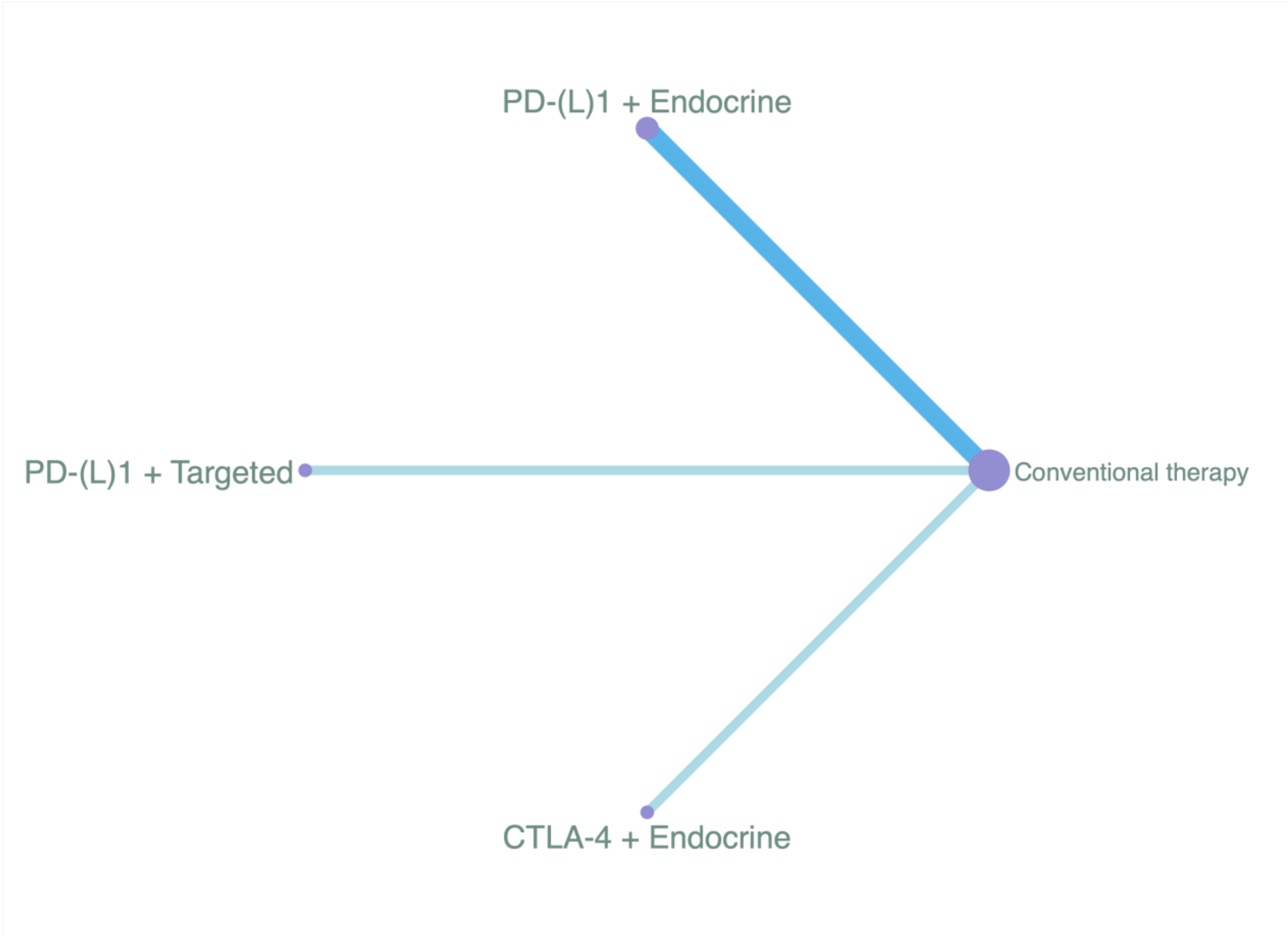

Figure S9. The SUCRA values of each dose modality for Hypothyroidism.

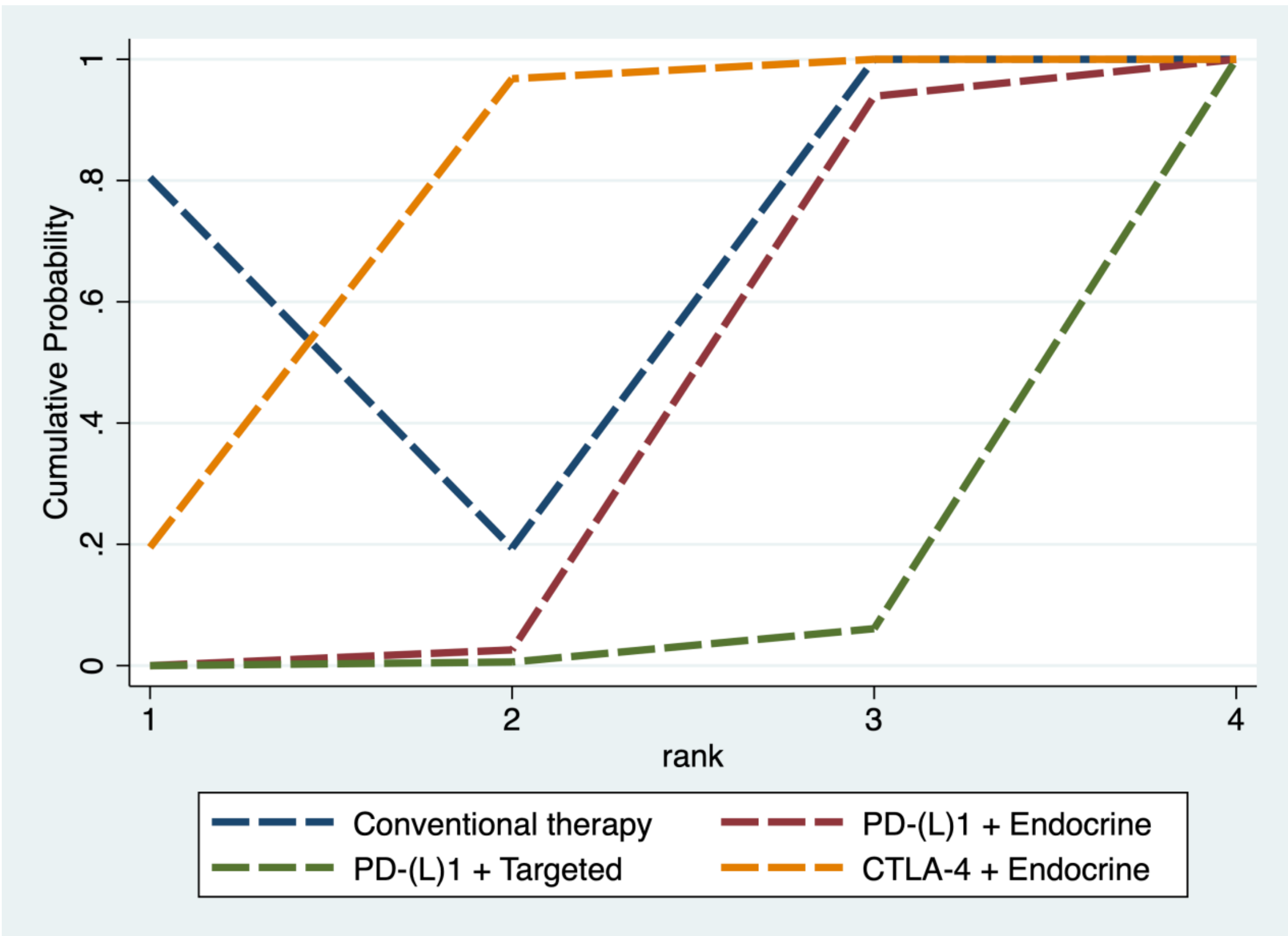

Figure S10. Comparison-adjusted funnel plot for Hypothyroidism.

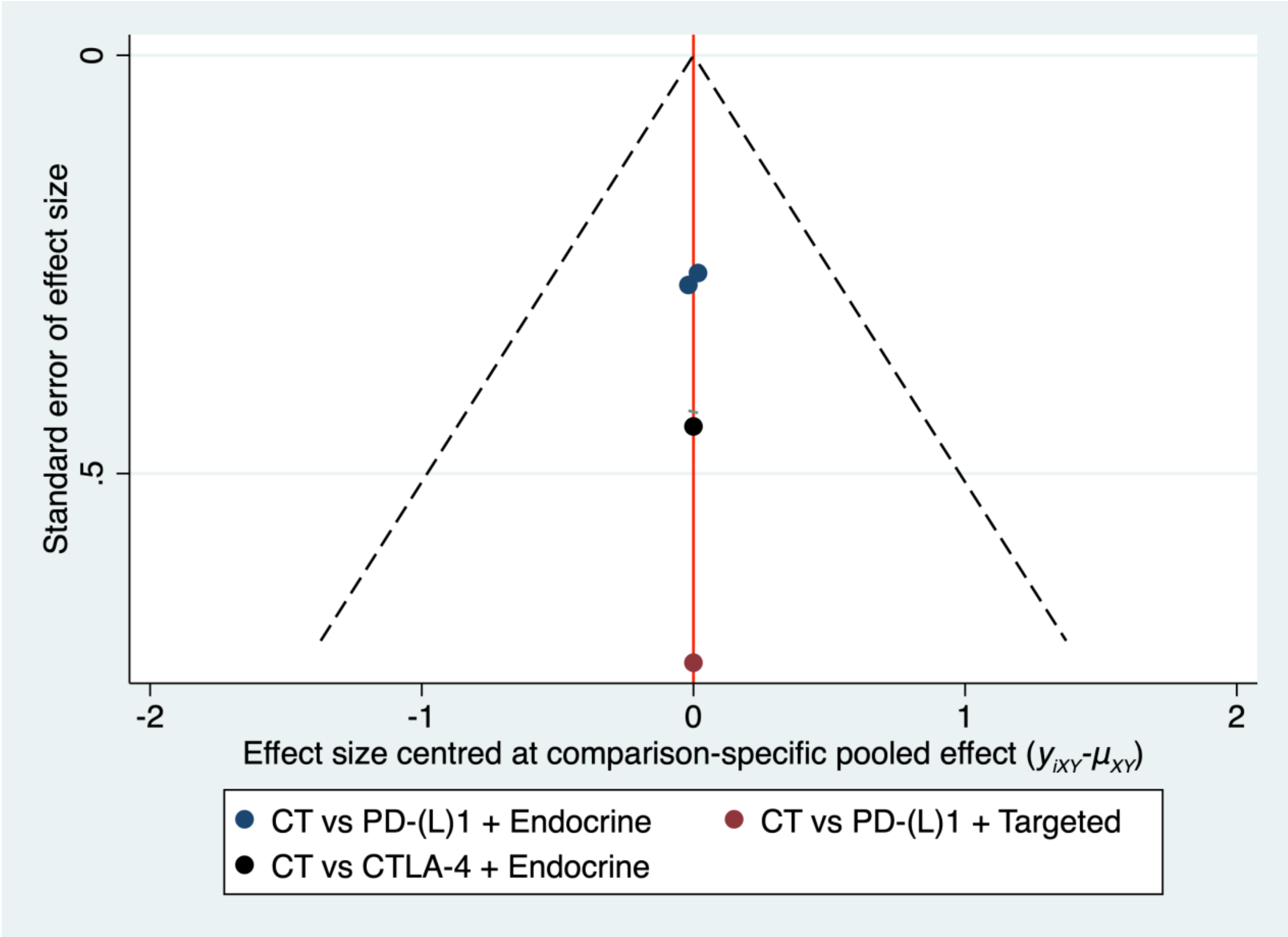

Figure S11 The evidence network of all papers on any grade of Fatigue as an adverse event.

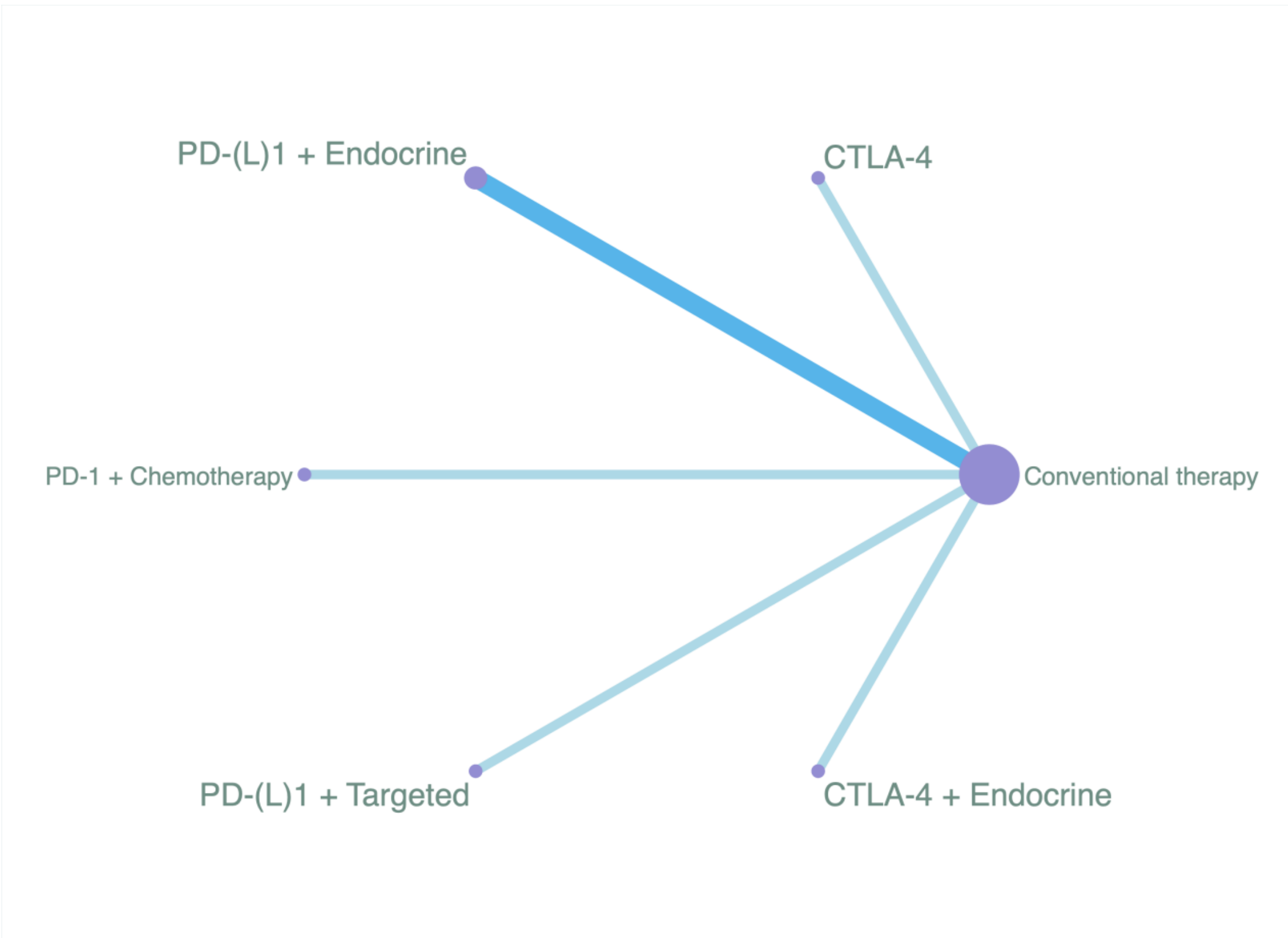

Figure S12. The SUCRA values of each dose modality for Fatigue.

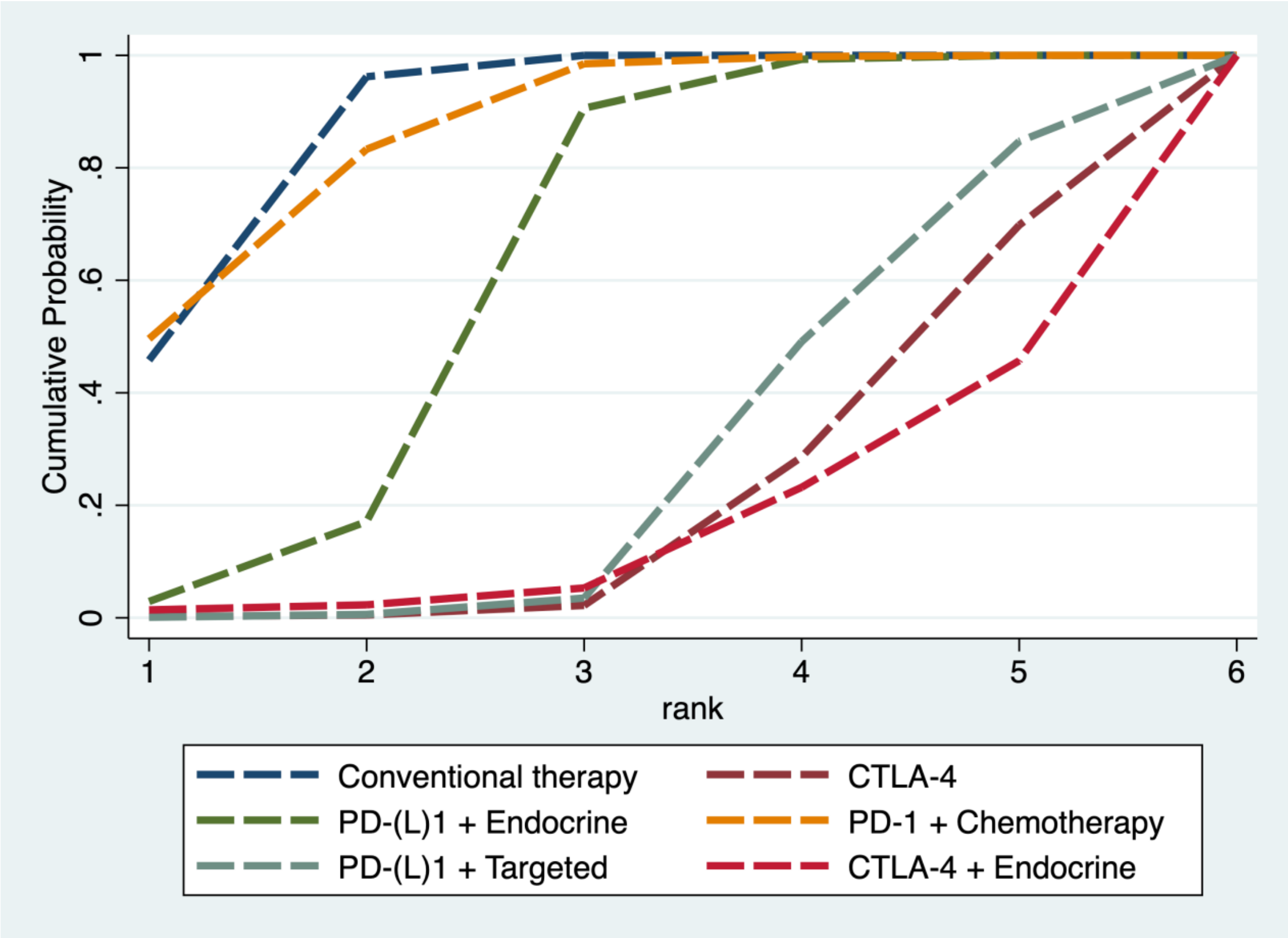

Figure S13. Comparison-adjusted funnel plot for Fatigue.

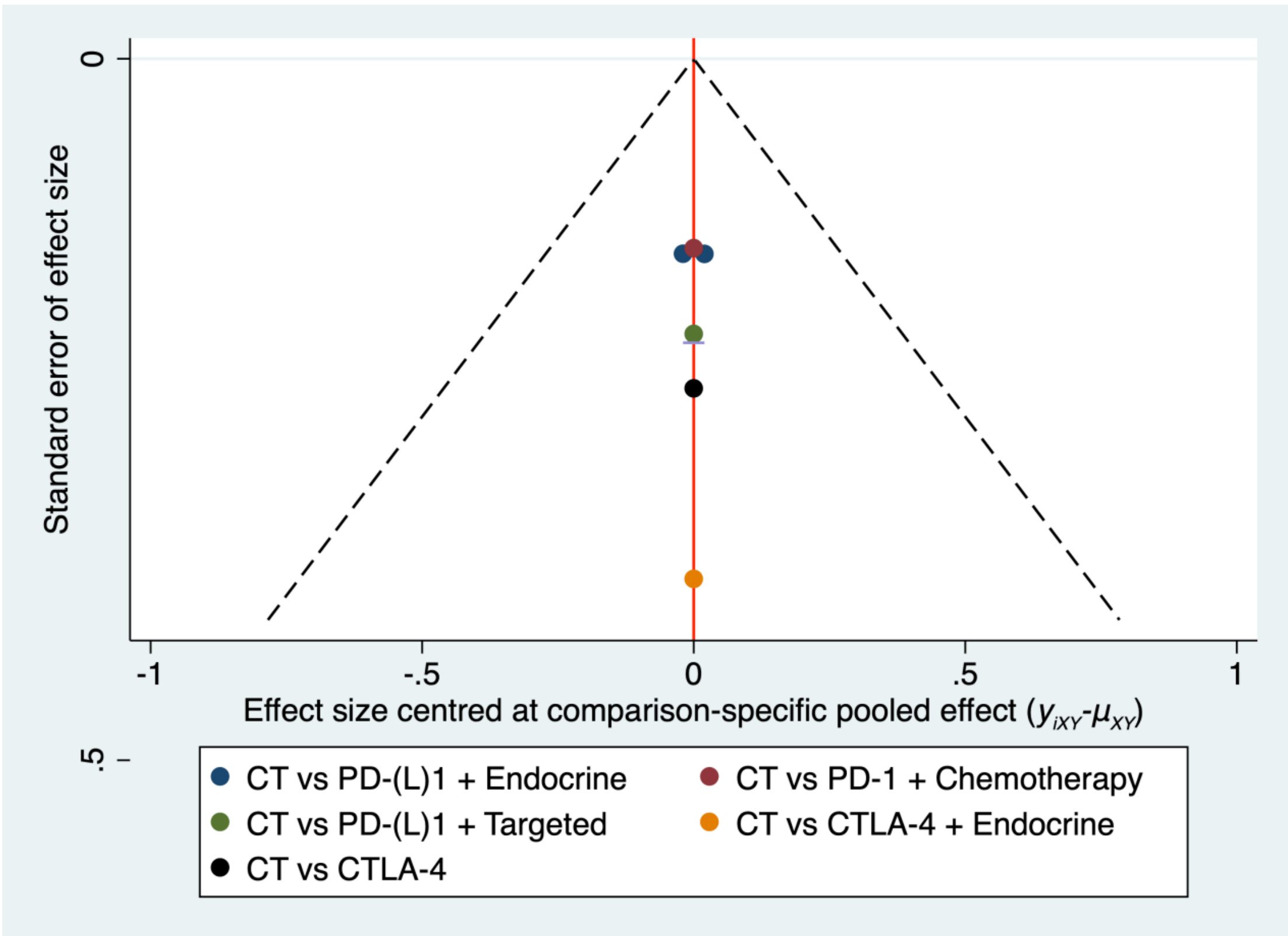

Figure S14. Meta analysis of ICI plus endocrine therapy versus control for any-grade adverse events.

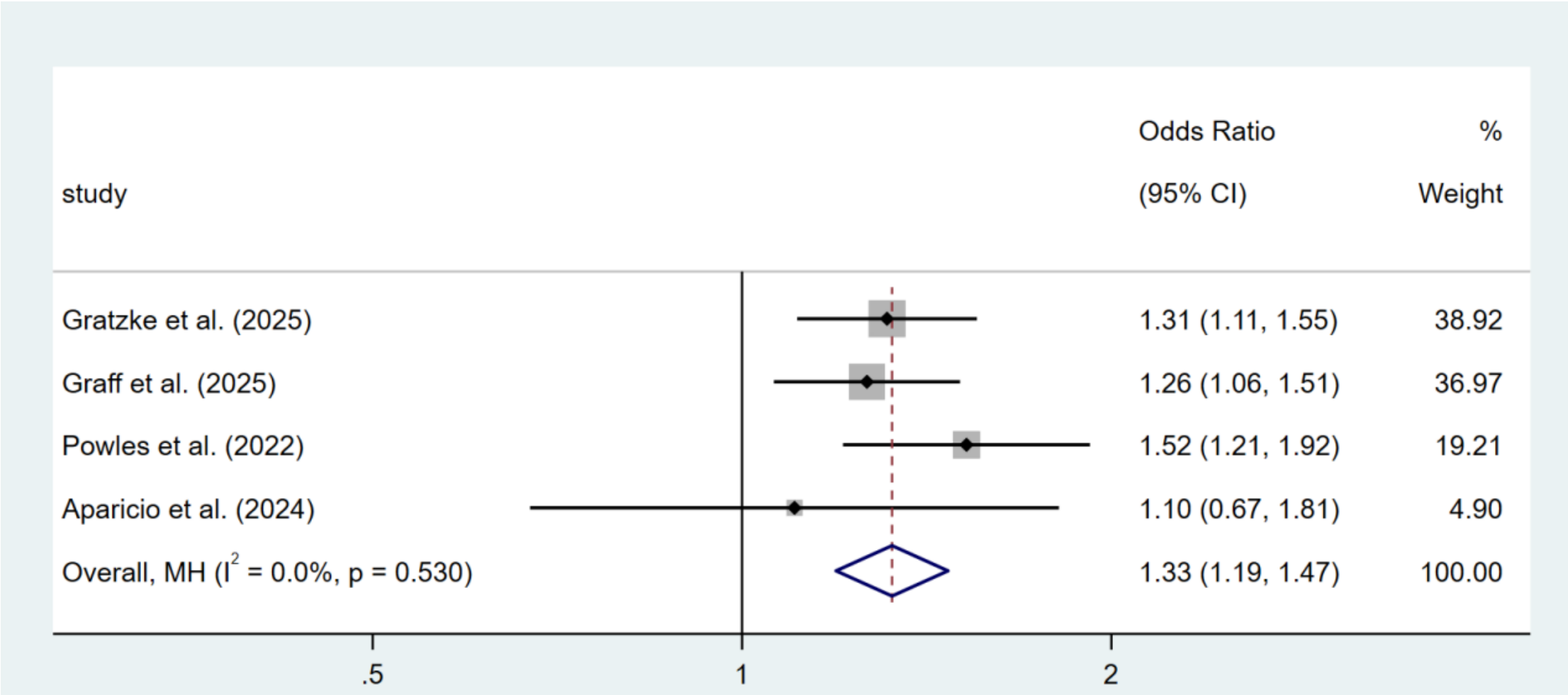

Figure S15. Leave-one-out sensitivity analysis of the endocrine therapy subgroup for any-grade adverse events.

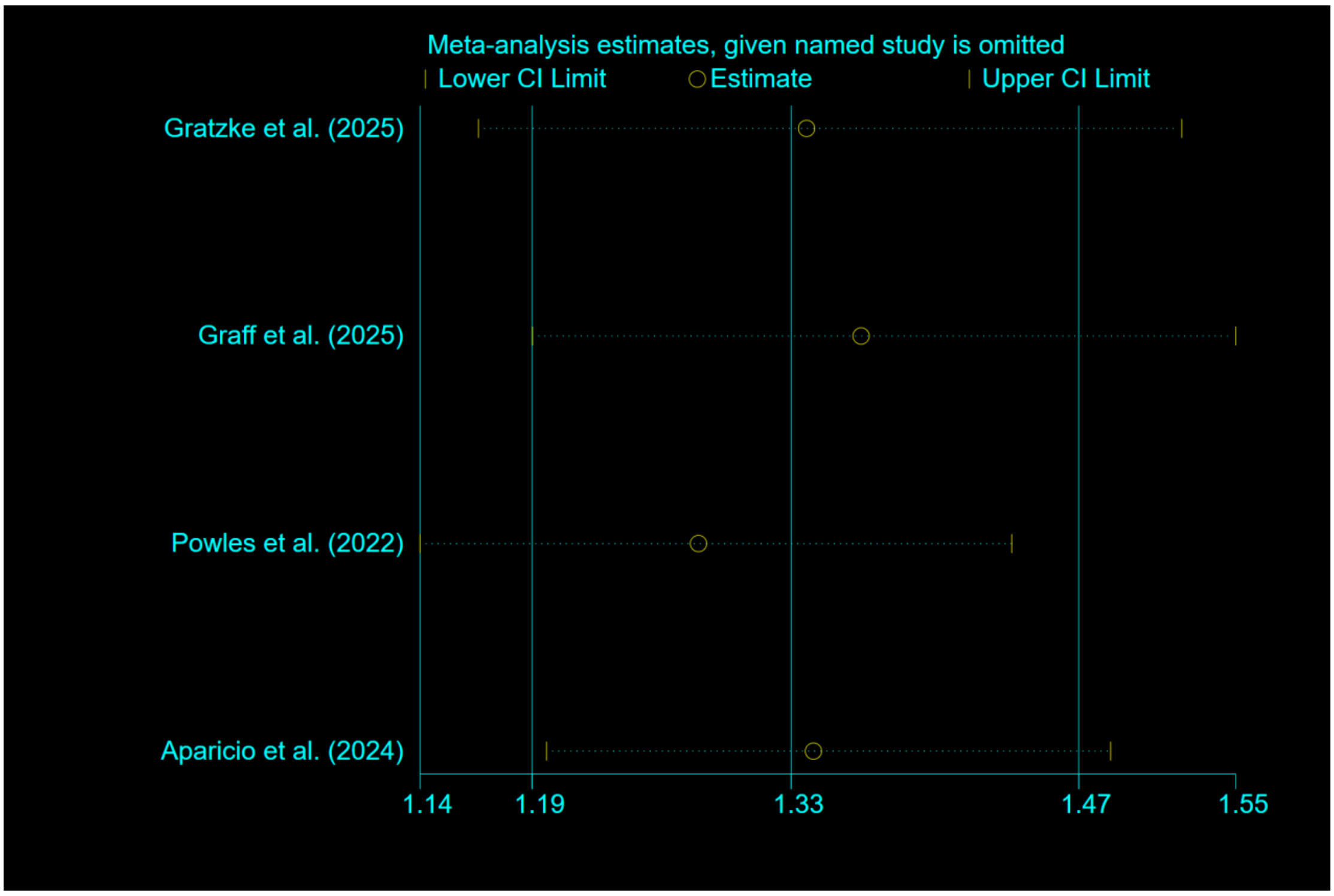

Figure S16. Meta analysis of ICI plus endocrine therapy versus control for grade ≥3 adverse events.

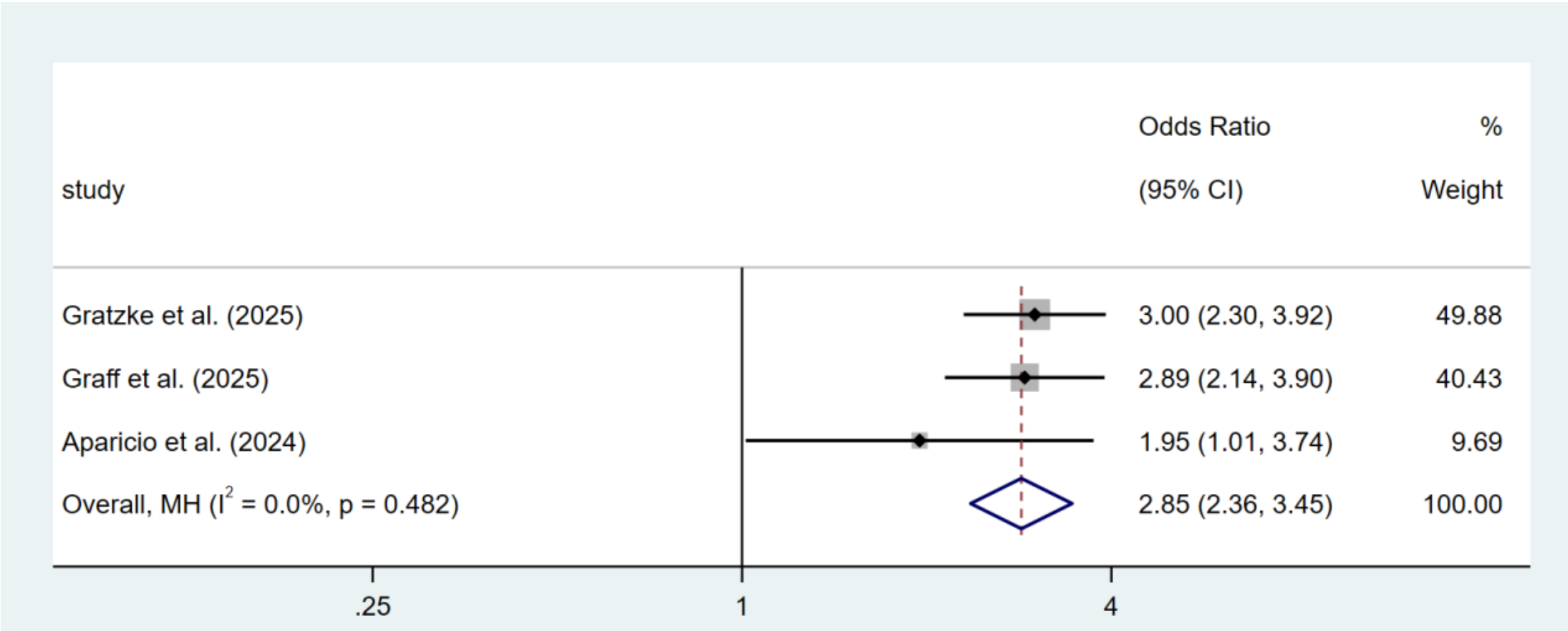

Figure S17. Leave-one-out sensitivity analysis of the endocrine therapy subgroup for grade ≥3 adverse events.

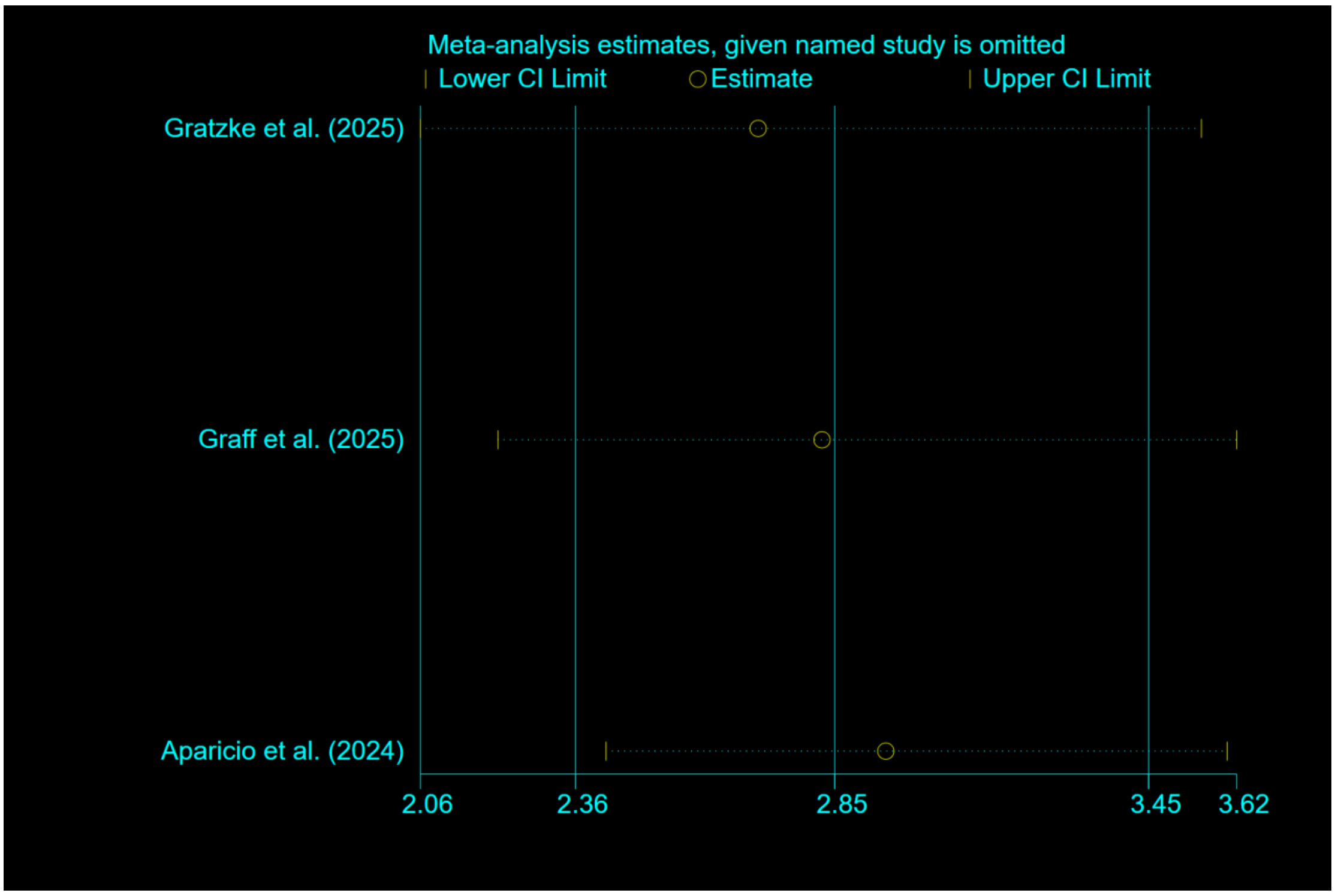

Figure S18. Meta analysis of ICI plus endocrine therapy versus control for serious adverse events.

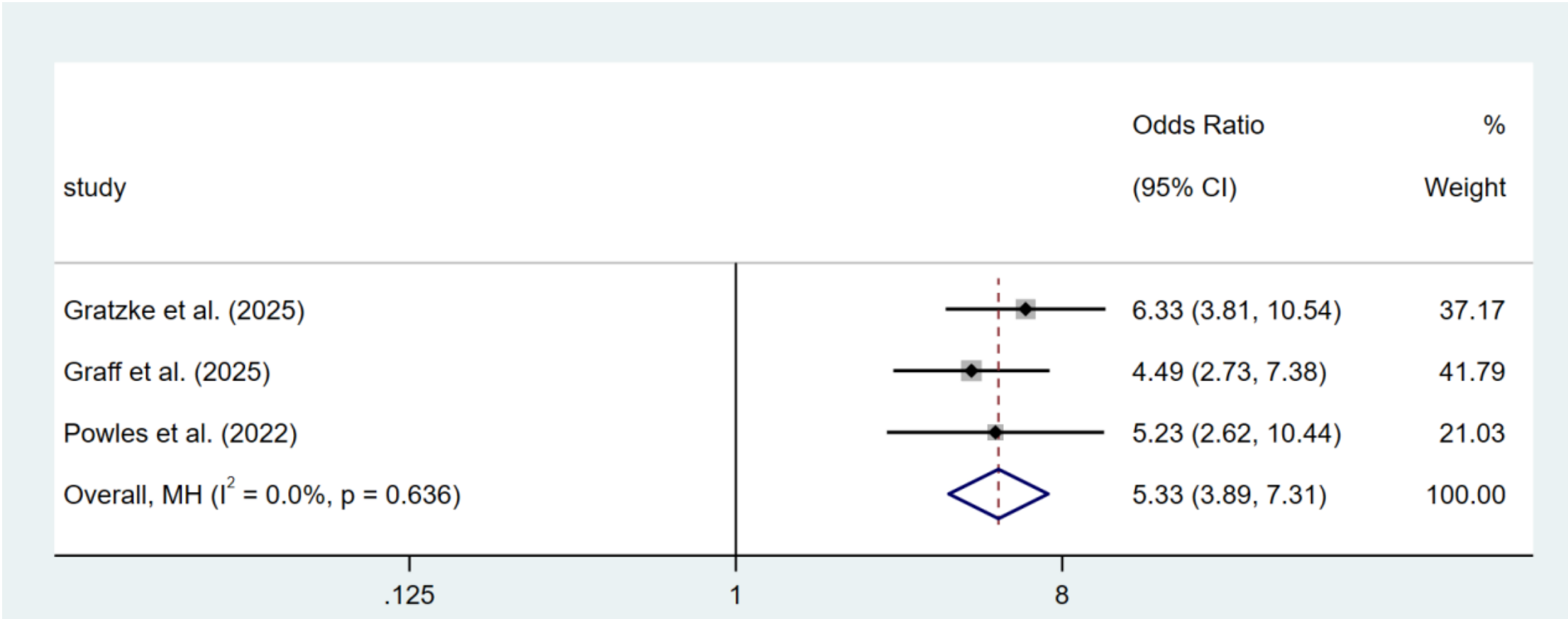

Figure S19. Leave-one-out sensitivity analysis of the endocrine therapy subgroup for serious adverse events.

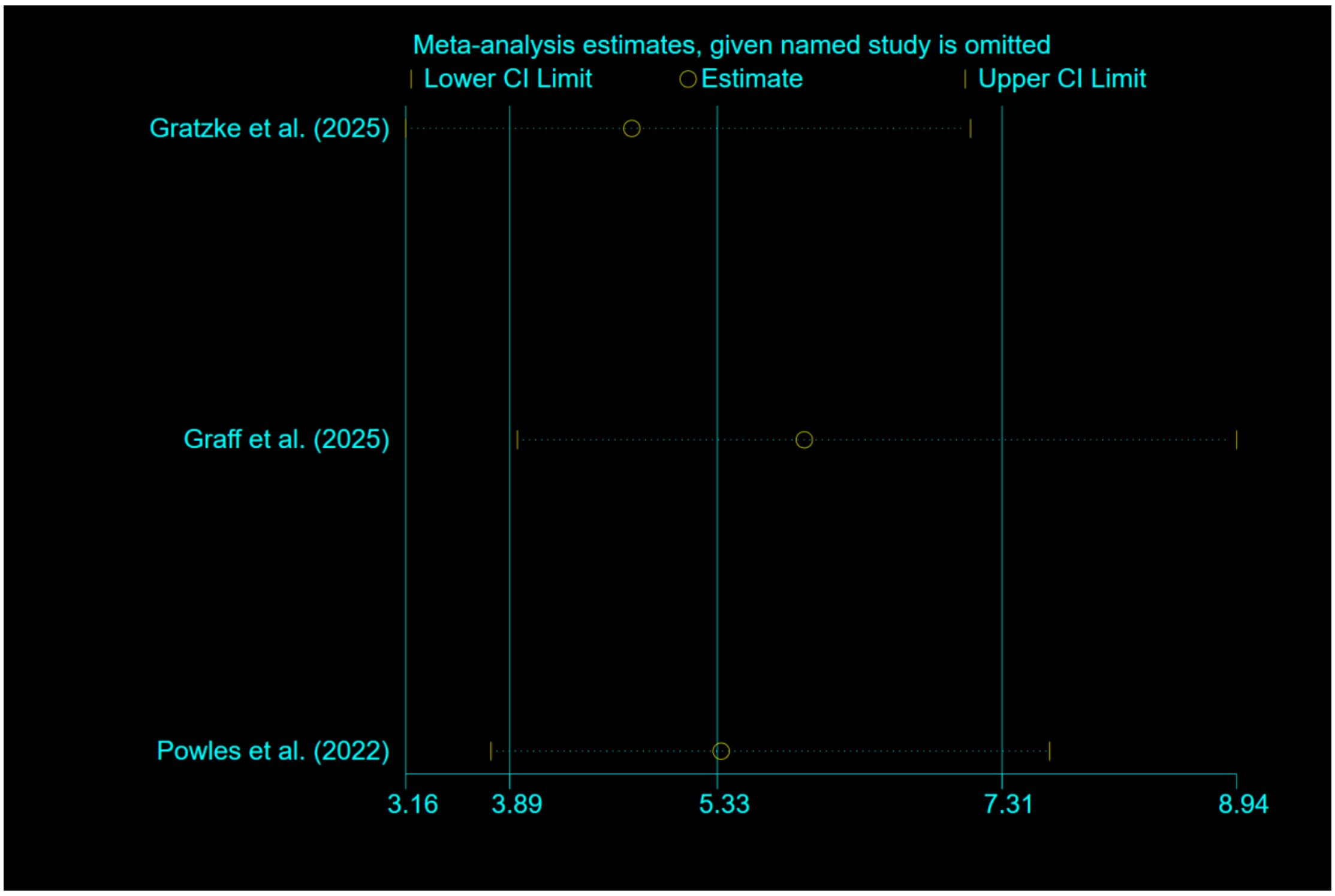

Figure S20. Meta analysis of ICI plus endocrine therapy versus control for fatal adverse events.

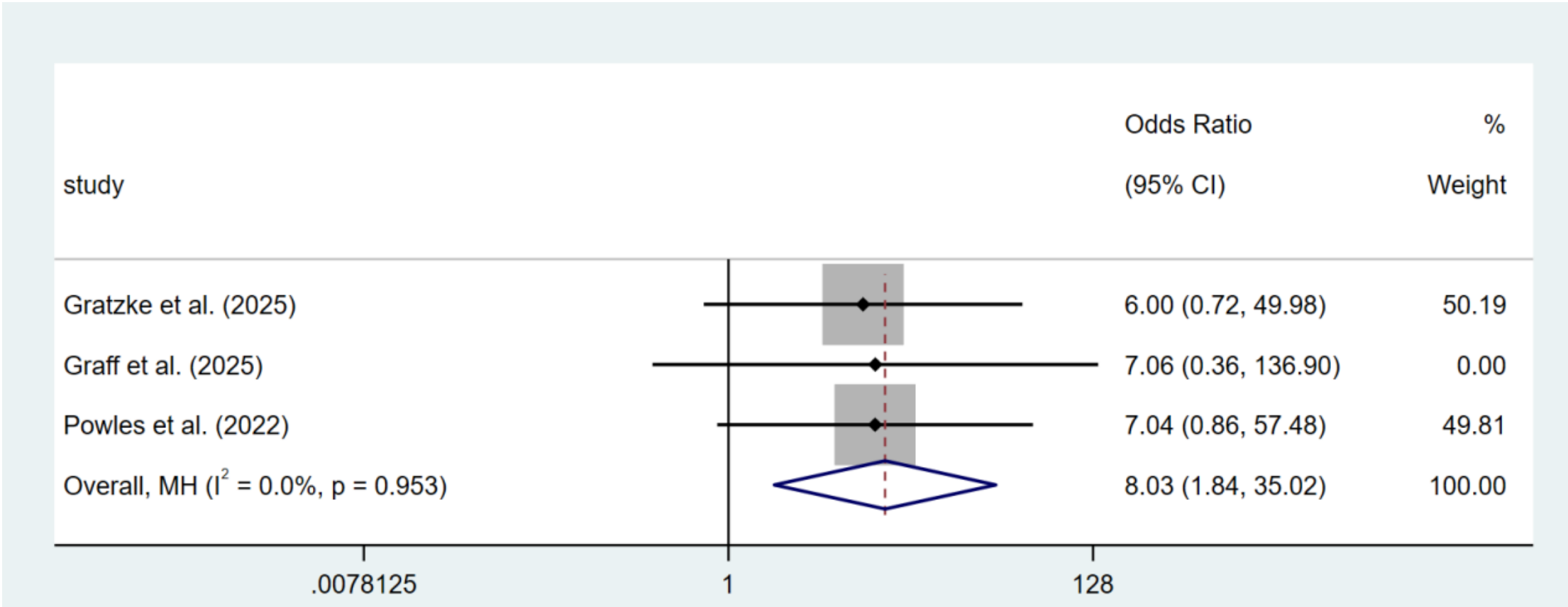

Figure S21. Leave-one-out sensitivity analysis of the endocrine therapy subgroup for fatal adverse events.

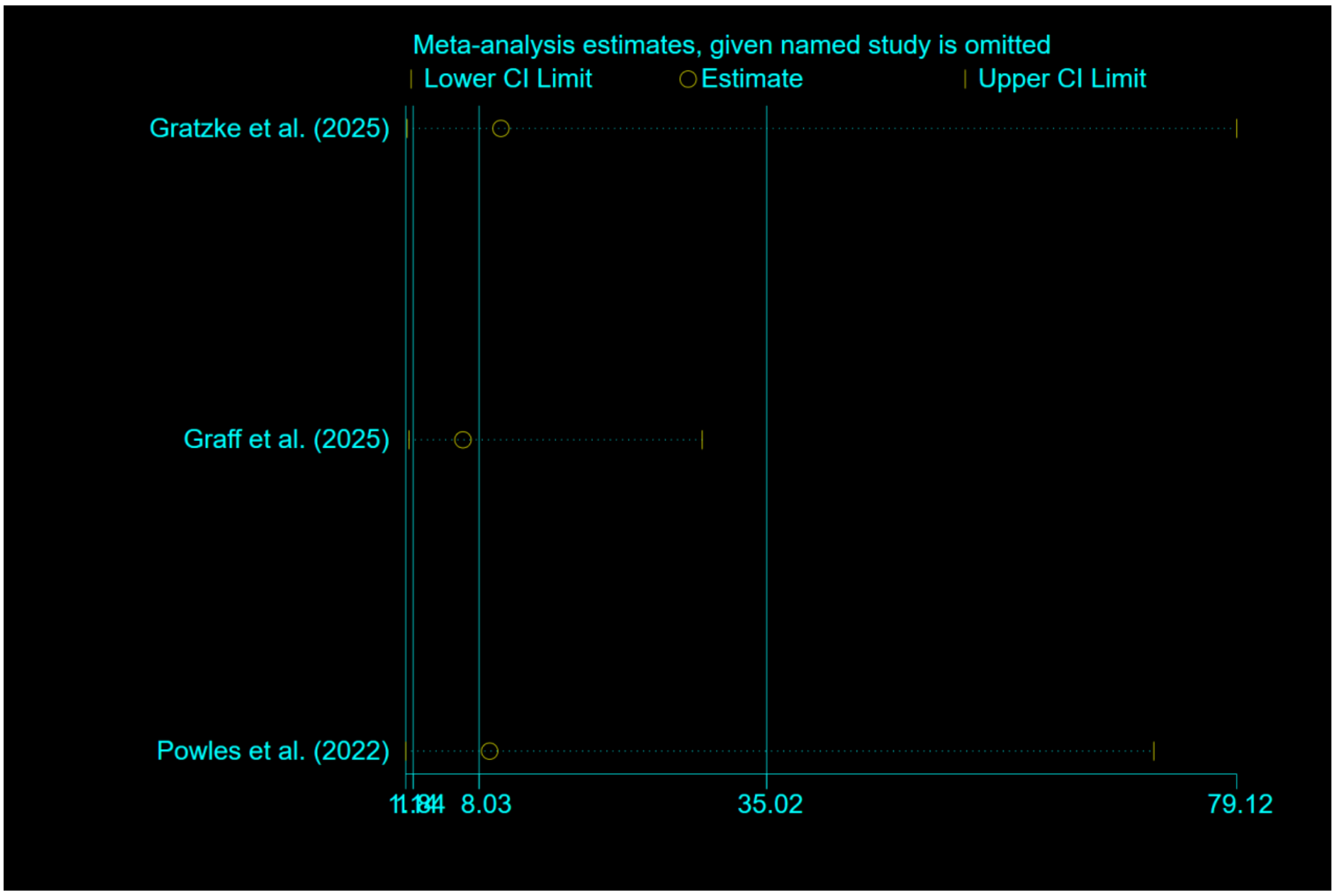

Figure S22. Meta analysis of ICI plus endocrine therapy versus control for any-cause adverse events.

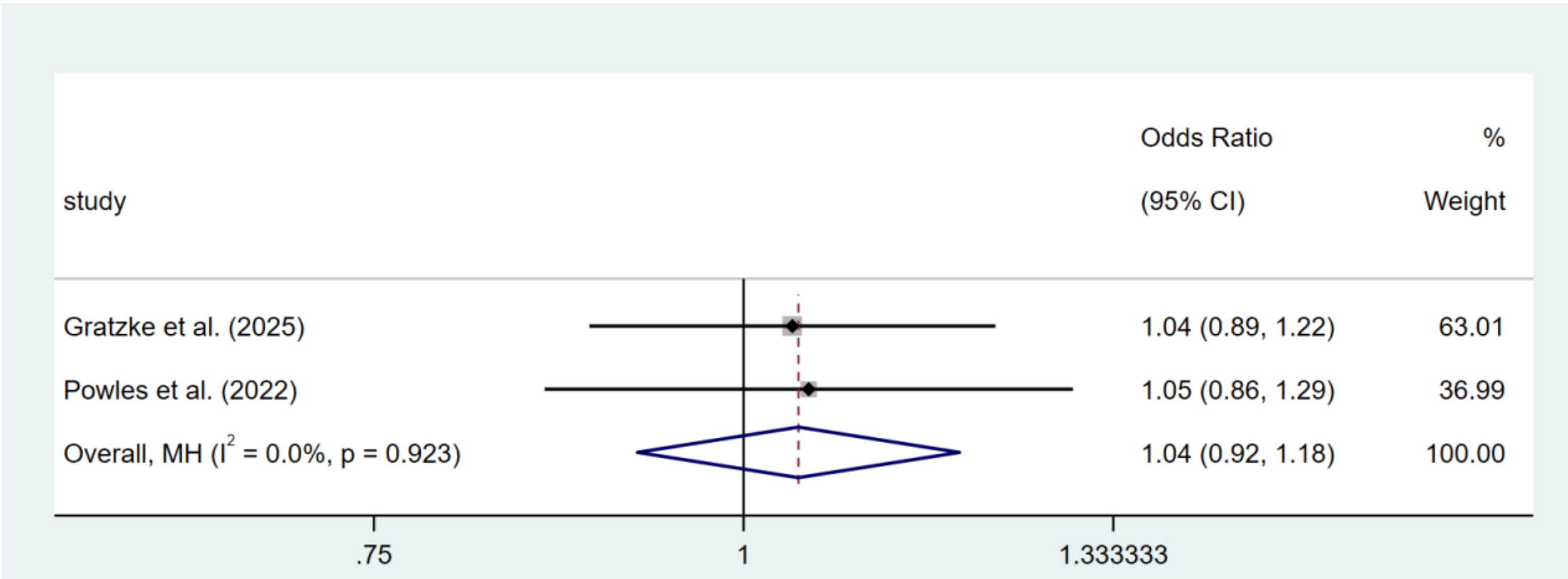

Figure S23. Leave-one-out sensitivity analysis of the endocrine therapy subgroup for any-cause adverse events.

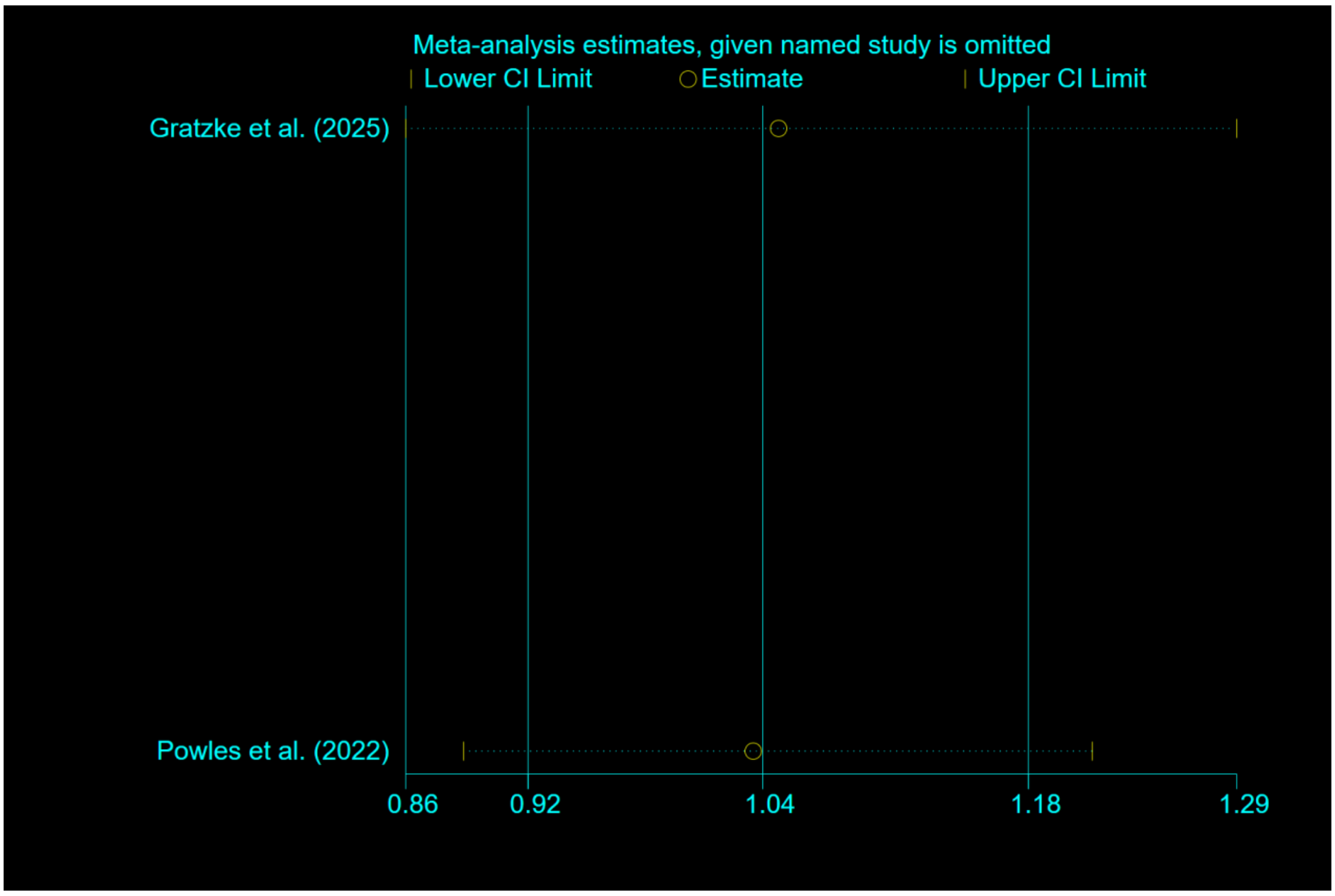

Figure S24. Funnel plot with pseudo 95% confidence limits for the endocrine therapy subgroup for any-grade adverse events.

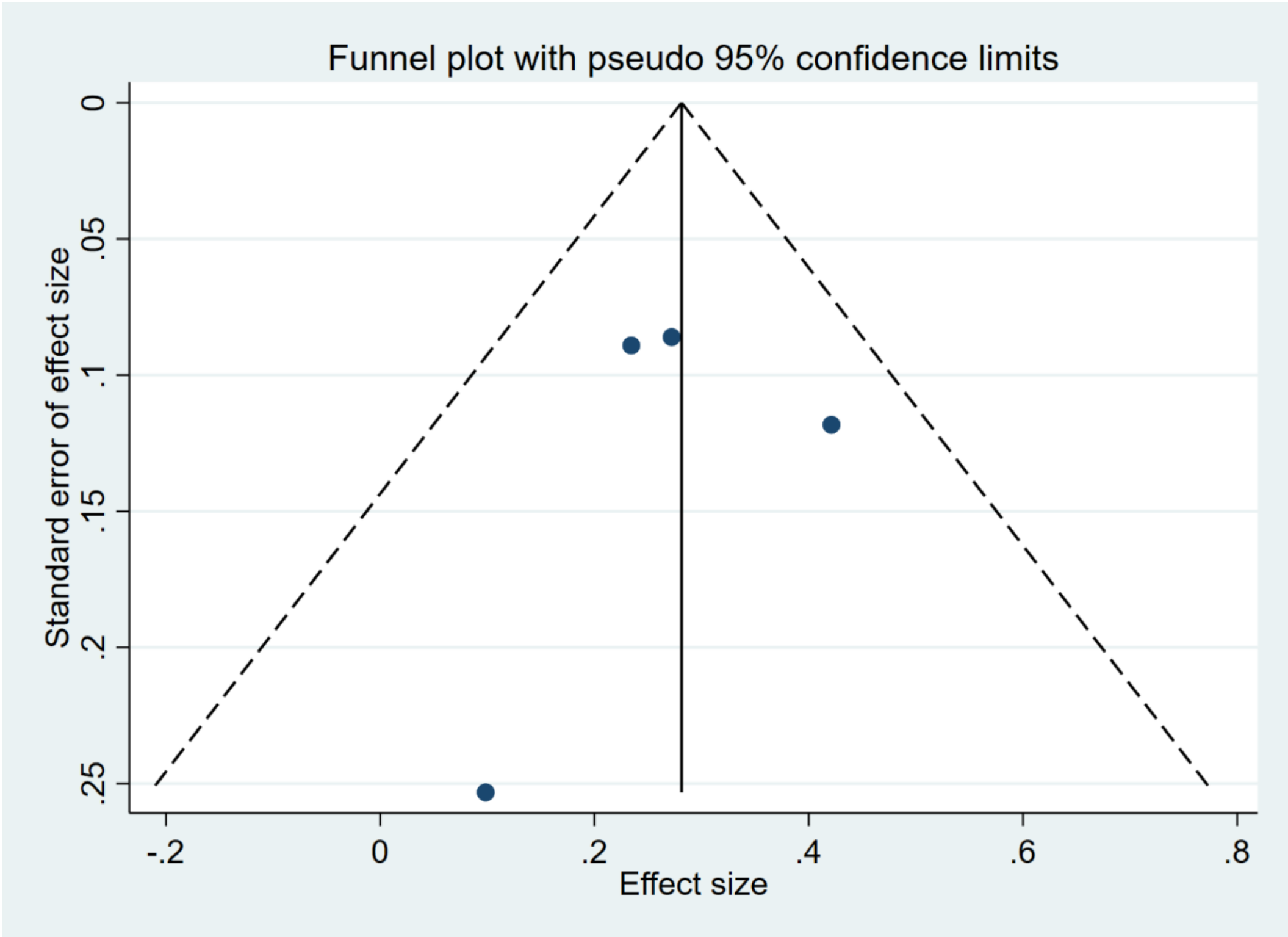

Figure S25. Funnel plot with pseudo 95% confidence limits for the endocrine therapy subgroup for grade ≥3 adverse events.

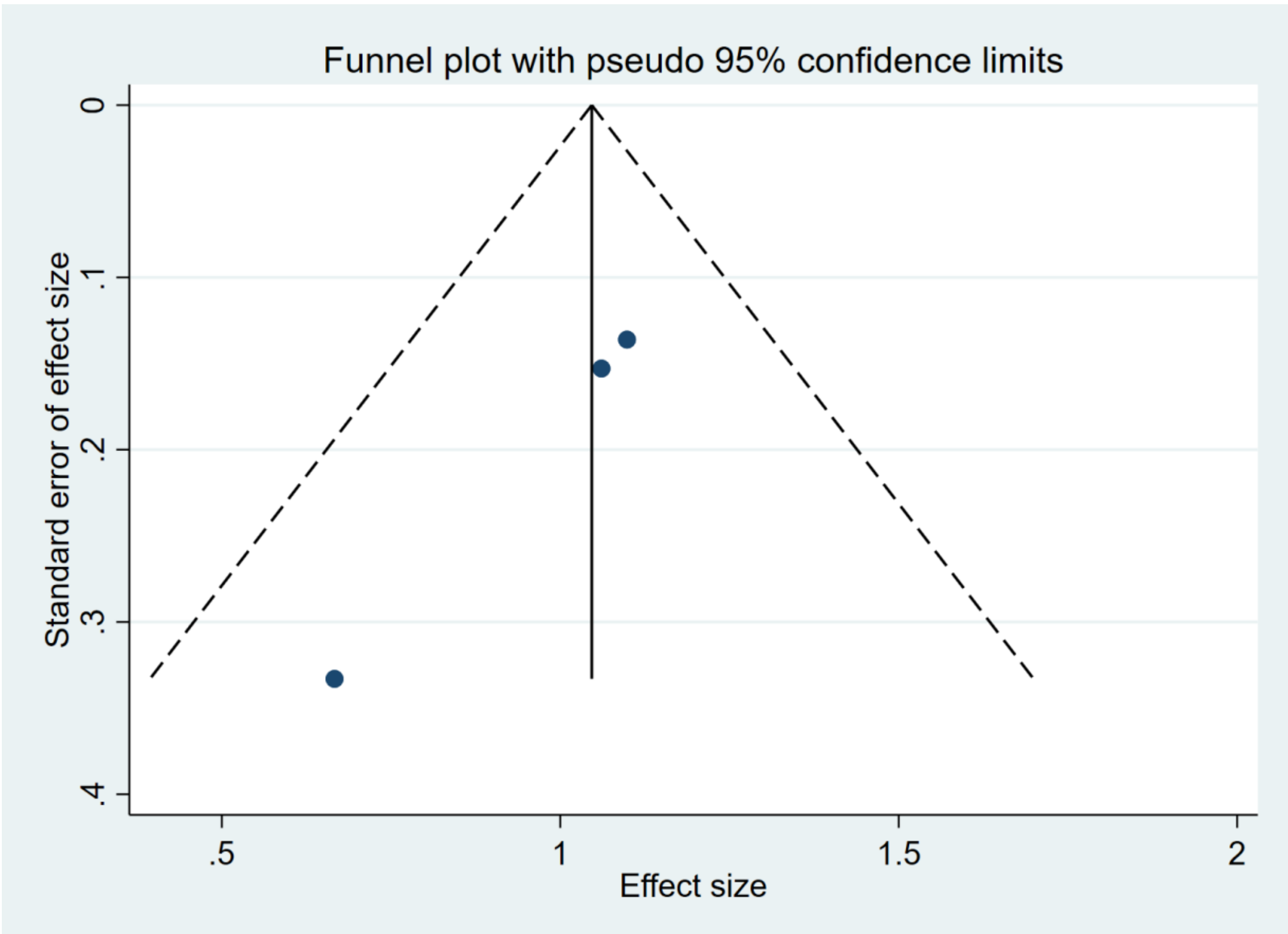

Figure S26. Funnel plot with pseudo 95% confidence limits for the endocrine therapy subgroup for serious adverse events.

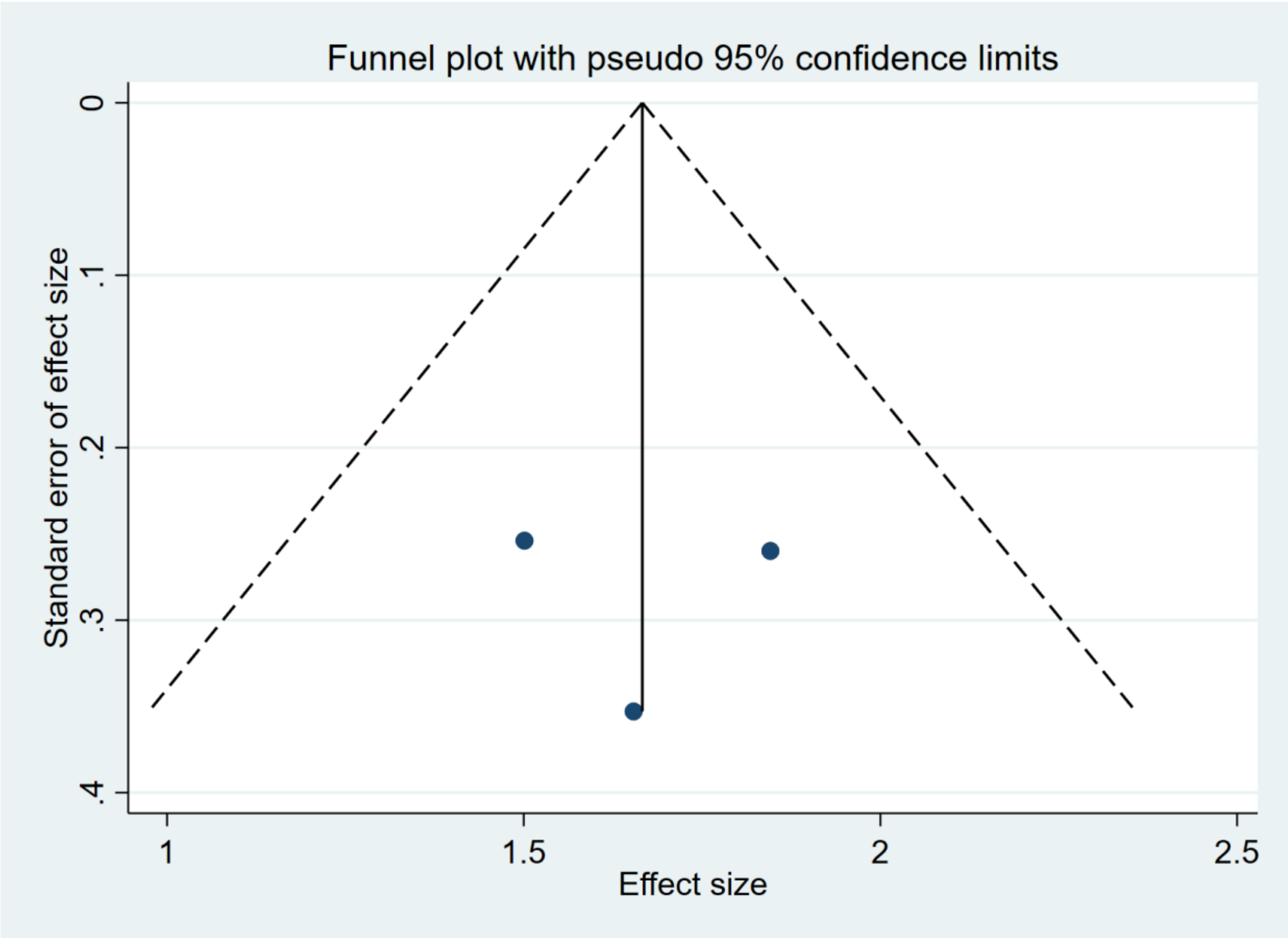

Figure S27. Funnel plot with pseudo 95% confidence limits for the endocrine therapy subgroup for fatal adverse events.

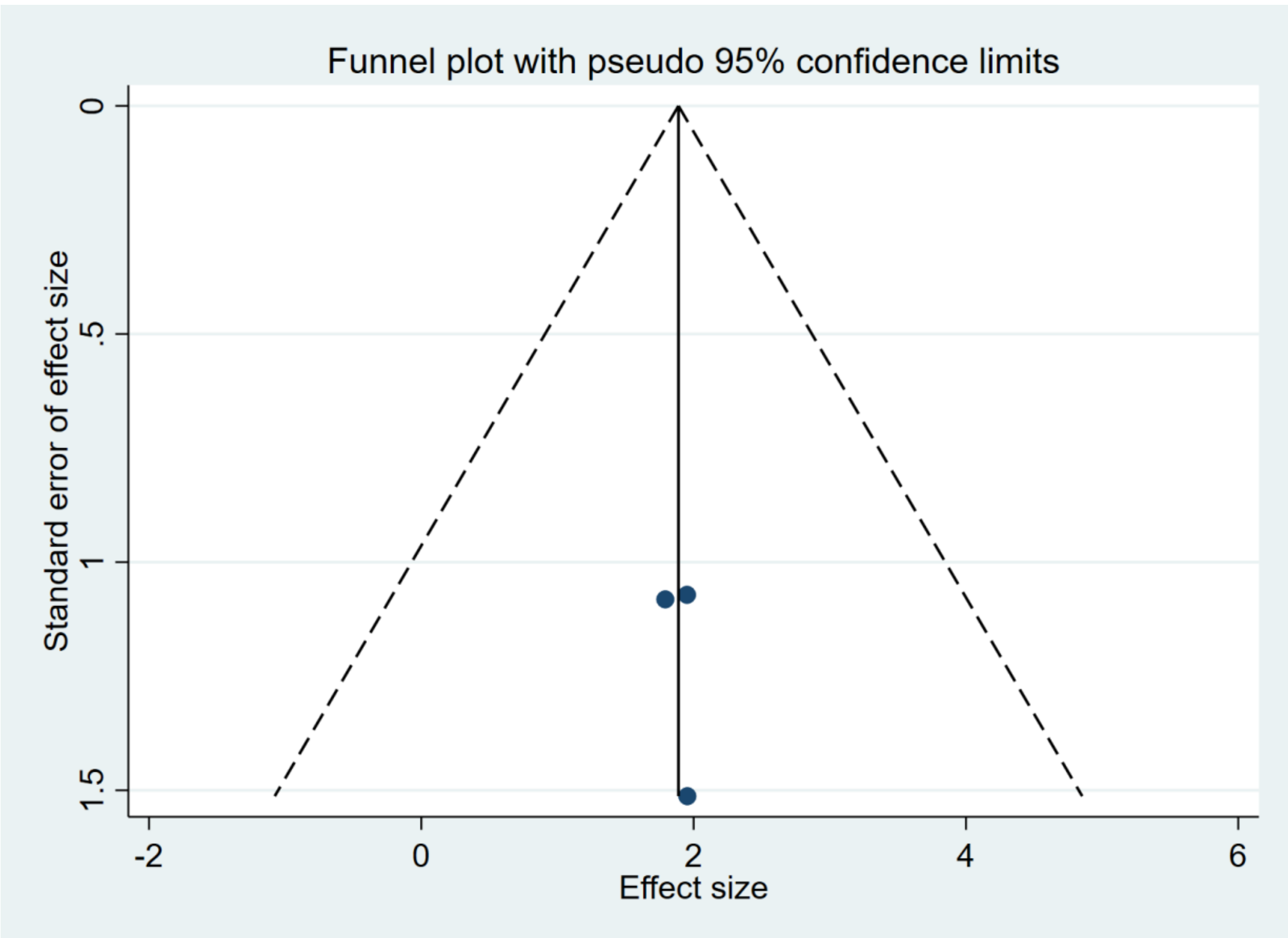

Figure S28. Funnel plot with pseudo 95% confidence limits for the endocrine therapy subgroup for any-cause adverse events.

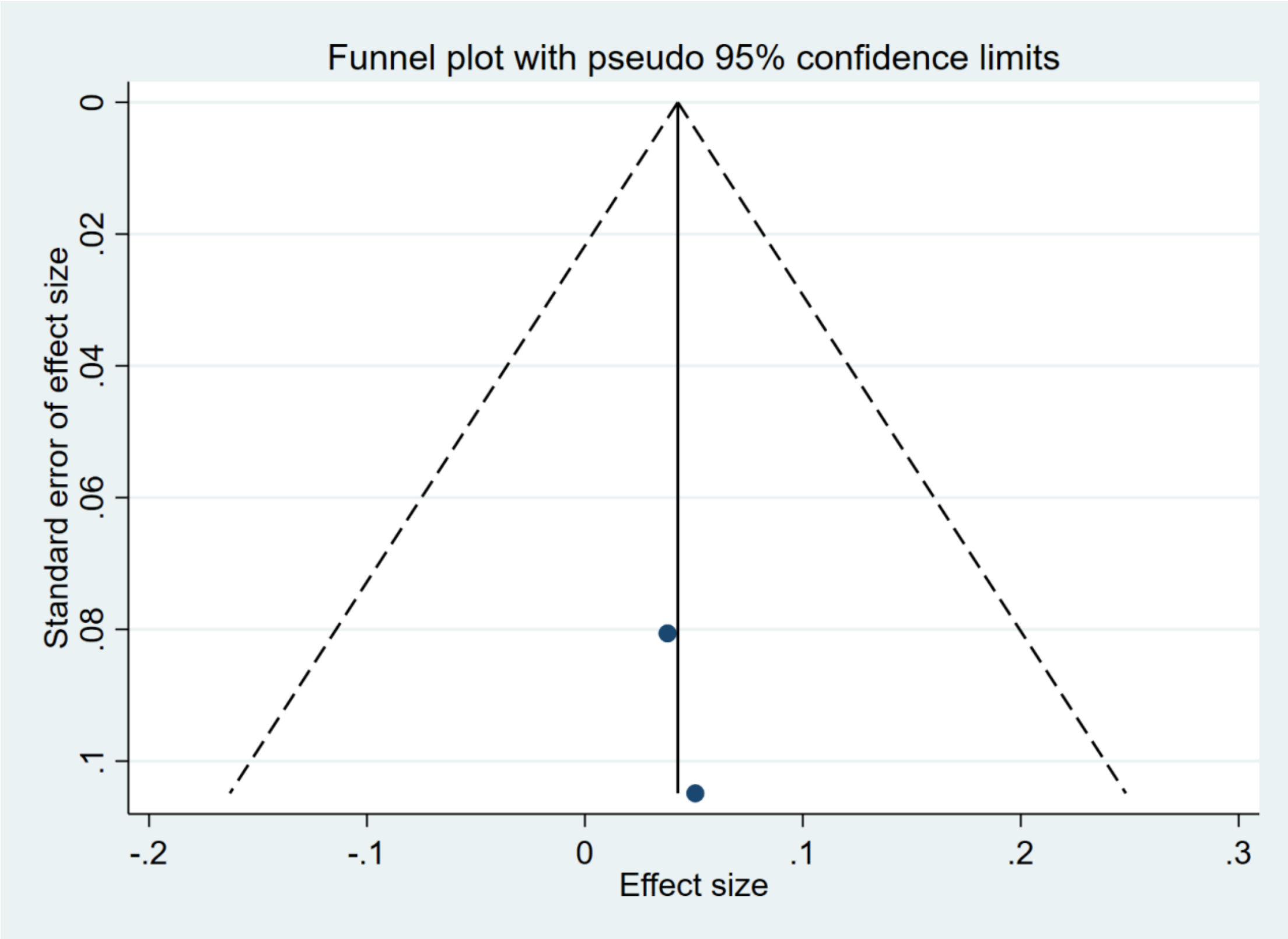

Appendix S1 Search strategy

Database (Search date)	Step	Search strategy	Number of results
Pubmed	#1	"Prostatic Neoplasms"[MeSH] OR Prostatic[tiab] OR prostate[tiab] OR "prostate neoplasm*"[tiab] OR "prostatic neoplasm*"[tiab] OR "prostate cancer*"[tiab] OR "prostatic cancer*"[tiab] OR "cancer of prostate"[tiab] OR "neoplasm of prostate"[tiab] OR "cancer of the prostate"[tiab] OR "neoplasm of the prostate"[tiab] OR "adenocarcinoma of prostate"[tiab] OR "adenocarcinoma of the prostate"[tiab] OR "squamous cell carcinoma of prostate"[tiab] OR "squamous cell carcinoma of the prostate"[tiab] OR "transitional cell carcinoma of prostate"[tiab] OR "transitional cell carcinoma of the prostate"[tiab] OR "cancers of prostate"[tiab] OR "neoplasms of prostate"[tiab] OR "cancers of the prostate"[tiab] OR "neoplasms of the prostate"[tiab] OR "adenocarcinomas of prostate"[tiab] OR "adenocarcinomas of the prostate"[tiab] OR "squamous cell carcinomas of prostate"[tiab] OR "squamous cell carcinomas of the prostate"[tiab] OR "transitional cell carcinomas of prostate"[tiab] OR "transitional cell carcinomas of the prostate"[tiab] OR "Prostatic Neoplasms, Castration-Resistant"[MeSH] OR "Castration-Resistant Prostate Neoplasm*"[tiab] OR "Castration Resistant Prostate Neoplasm*"[tiab] OR "Castration-Resistant Prostate Cancer*"[tiab] OR "Castration Resistant Prostate cancer*"[tiab] OR "Androgen-Resistant Prostate Neoplasm*"[tiab] OR "Androgen Resistant Prostate Neoplasm*"[tiab] OR "Androgen-Resistant Prostate Cancer*"[tiab] OR "Androgen Resistant Prostate cancer*"[tiab] OR "Androgen-Insensitive Prostate Neoplasm*"[tiab] OR "Androgen Insensitive Prostate Neoplasm*"[tiab] OR "Androgen-Insensitive Prostate Cancer*"[tiab] OR "Androgen Insensitive Prostate cancer*"[tiab] OR "Androgen-Independent Prostate Neoplasm*"[tiab] OR "Androgen Independent Prostate Neoplasm*"[tiab] OR "Androgen-Independent Prostate Cancer*"[tiab] OR "Androgen Independent Prostate cancer*"[tiab] OR "Hormone refractory Prostate Neoplasm*"[tiab] OR "Hormone refractory Prostate Neoplasm*"[tiab] OR "Hormone refractory Prostate Cancer*"[tiab] OR "Hormone refractory Prostate cancer*"[tiab] OR "Castration-Resistant Prostatic Neoplasm*"[tiab] OR "Castration Resistant Prostatic Neoplasm*"[tiab] OR "Castration-Resistant Prostatic Cancer*"[tiab] OR "Castration Resistant Prostatic cancer*"[tiab] OR "Androgen-Resistant Prostatic Neoplasm*"[tiab] OR "Androgen Resistant Prostatic Neoplasm*"[tiab] OR "Androgen-Resistant Prostatic Cancer*"[tiab] OR "Androgen Resistant Prostatic cancer*"[tiab] OR "Androgen-Insensitive Prostatic Neoplasm*"[tiab] OR "Androgen Insensitive Prostatic Neoplasm*"[tiab] OR "Androgen-Insensitive Prostatic Cancer*"[tiab] OR "Androgen Insensitive Prostatic cancer*"[tiab] OR "Androgen-Independent Prostatic Neoplasm*"[tiab] OR "Androgen Independent Prostatic Neoplasm*"[tiab] OR "Androgen-Independent Prostatic Cancer*"[tiab] OR "Androgen Independent Prostatic cancer*"[tiab] OR "Hormone refractory Prostatic Neoplasm*"[tiab] OR "Hormone refractory Prostatic Neoplasm*"[tiab] OR "Hormone refractory Prostatic Cancer*"[tiab] OR "Hormone refractory Prostatic cancer*"[tiab]	244,576
	#2	"Immune Checkpoint Inhibitors"[MeSH] OR "Immune Checkpoint Inhibitor*"[tiab] OR "Immune Checkpoint Blocker*"[tiab] OR "Immune Checkpoint Blockade*"[tiab] OR "Immune Checkpoint Inhibition*"[tiab] OR "PD-L1 Inhibitor*"[tiab] OR "PD L1 Inhibitor*"[tiab] OR "PDL1 Inhibitor*"[tiab] OR "PD-L1 blocker*"[tiab] OR "PD L1 blocker*"[tiab] OR "PDL1 blocker*"[tiab] OR "Programmed Death-Ligand 1 Inhibitor*"[tiab] OR "Programmed Death	41,660

		Ligand 1 Inhibitor*"[tiab] OR "Programmed Death-Ligand 1 blocker*"[tiab] OR "Programmed Death Ligand 1 blocker*"[tiab] OR "anti Programmed Death-Ligand 1"[tiab] OR "anti-Programmed Death-Ligand 1"[tiab] OR "anti Programmed Death Ligand 1"[tiab] OR "anti-Programmed Death Ligand 1"[tiab] OR "CTLA-4 Inhibitor*"[tiab] OR "CTLA 4 Inhibitor*"[tiab] OR "CTLA4 Inhibitor*"[tiab] OR "CTLA-4 blocker*"[tiab] OR "CTLA 4 blocker*"[tiab] OR "CTLA4 blocker*"[tiab] OR "Cytotoxic T-Lymphocyte Associated Protein 4 Inhibitor*"[tiab] OR "Cytotoxic T Lymphocyte-Associated Protein 4 Inhibitor*"[tiab] OR "Cytotoxic T-Lymphocyte Associated Protein 4 blocker*"[tiab] OR "Cytotoxic T Lymphocyte-Associated Protein 4 blocker*"[tiab] OR "Cytotoxic T-Lymphocyte Associated antigen 4 Inhibitor*"[tiab] OR "Cytotoxic T Lymphocyte-Associated antigen 4 Inhibitor*"[tiab] OR "Cytotoxic T-Lymphocyte Associated antigen 4 blocker*"[tiab] OR "Cytotoxic T Lymphocyte-Associated antigen 4 blocker*"[tiab] OR "Cytotoxic T-Lymphocyte-Associated Protein 4 Inhibitor*"[tiab] OR "Cytotoxic T Lymphocyte Associated Protein 4 Inhibitor*"[tiab] OR "Cytotoxic T-Lymphocyte-Associated Protein 4 blocker*"[tiab] OR "Cytotoxic T Lymphocyte Associated Protein 4 blocker*"[tiab] OR "Cytotoxic T-Lymphocyte-Associated antigen 4 Inhibitor*"[tiab] OR "Cytotoxic T Lymphocyte Associated antigen 4 Inhibitor*"[tiab] OR "Cytotoxic T-Lymphocyte-Associated antigen 4 blocker*"[tiab] OR "Cytotoxic T Lymphocyte Associated antigen 4 blocker*"[tiab] OR "anti-cytotoxic T lymphocyte-associated antigen 4"[tiab] OR "anti cytotoxic T lymphocyte-associated antigen 4"[tiab] OR "anti-cytotoxic T lymphocyte-associated protein 4"[tiab] OR "anti cytotoxic T lymphocyte-associated protein 4"[tiab] OR "anti-cytotoxic T-lymphocyte-associated antigen 4"[tiab] OR "anti-cytotoxic T-lymphocyte-associated antigen 4"[tiab] OR "PD-1 Inhibitor*"[tiab] OR "PD 1 Inhibitor*"[tiab] OR "PD1 Inhibitor*"[tiab] OR "PD 1 blocker*"[tiab] OR "PD1 blocker*"[tiab] OR "PD-1 blocker*"[tiab] OR "Programmed Cell Death Protein 1 Inhibitor*"[tiab] OR "Programmed Cell Death Protein 1 blocker*"[tiab] OR "anti Programmed Cell Death Protein 1"[tiab] OR "anti-Programmed Cell Death Protein 1"[tiab] OR "anti-PD1"[tiab] OR "anti PD1"[tiab] OR "anti-PD-1"[tiab] OR "anti PD-1"[tiab] OR "anti-PD-L1"[tiab] OR "anti PDL1"[tiab] OR "anti PD-L1"[tiab] OR "anti-PDL1"[tiab] OR "anti-PD L1"[tiab] OR "anti PD L1"[tiab] OR "anti-CTLA4"[tiab] OR "anti-CTLA 4"[tiab] OR "anti-CTLA-4"[tiab] OR "anti CTLA4"[tiab] OR "anti CTLA 4"[tiab] OR "anti CTLA-4"[tiab] OR "PD-1-PD-L1 Blockade*"[tiab] OR "PD 1 PD L1 Blockade*"[tiab] OR "Pembrolizumab"[tiab] OR "MK-3475"[tiab] OR "lambrolizumab"[tiab] OR "Keytruda"[tiab] OR "SCH-900475"[tiab] OR "Nivolumab"[tiab] OR "Opdivo"[tiab] OR "ONO-4538"[tiab] OR "ONO 4538"[tiab] OR "ONO4538"[tiab] OR "MDX-1106"[tiab] OR "MDX 1106"[tiab] OR "MDX1106"[tiab] OR "BMS-936558"[tiab] OR "BMS 936558"[tiab] OR "BMS936558"[tiab] OR "Ipilimumab"[tiab] OR "Yervoy"[tiab] OR "MDX 010"[tiab] OR "MDX010"[tiab] OR "MDX-010"[tiab] OR "MDX-CTLA-4"[tiab] OR "MDX CTLA 4"[tiab] OR "Durvalumab"[tiab] OR "MEDI4736"[tiab] OR "MEDI-4736"[tiab] OR "Imfinzi"[tiab] OR "Dostarlimab"[tiab] OR "GSK4057190"[tiab] OR "TSR-042"[tiab] OR "Cemiplimab"[tiab] OR "REGN2810"[tiab] OR "Avelumab"[tiab] OR "MSB-0010682"[tiab] OR "MSB0010682"[tiab] OR "bavencio"[tiab] OR "MSB0010718C"[tiab] OR "MSB-0010718C"[tiab] OR "Atezolizumab"[tiab] OR "MPDL3280A"[tiab] OR "MPDL-3280A"[tiab] OR "Tecentriq"[tiab] OR "RG7446"[tiab] OR "RG-7446"[tiab] OR "pidilizumab"[tiab] OR "CT-011"[tiab] OR "CT 011"[tiab] OR "tremelimumab"[tiab] OR "ticilimumab"[tiab] OR "CP 675"[tiab] OR "CP675 cpd"[tiab] OR "CP-675"[tiab] OR "CP-675,206"[tiab] OR "CP-	

		675206"[tiab] OR "CP675206"[tiab] OR "CP 675206"[tiab] OR "dostarlimab"[Supplementary Concept] OR "cemiplimab"[Supplementary Concept] OR "Ipilimumab"[Mesh] OR "tremelimumab"[Supplementary Concept] OR "pembrolizumab"[Supplementary Concept] OR "atezolizumab"[Supplementary Concept] OR "Nivolumab"[Mesh] OR "durvalumab"[Supplementary Concept] OR "avelumab" [Supplementary Concept] OR "pidilizumab" [Supplementary Concept] OR "sintilimab"[tiab] OR "SHR-1210"[taib] OR "camrelizumab"[tiab] OR "toripalimab"[tiab] OR "HX008"[tiab] OR "tremelimumab"[tiab]	
	#3	"RCT"[tiab] OR "trial*"[tiab] OR "intervention*"[tiab] OR "placebo"[tiab] OR "randomised trial*"[tiab] OR "randomized trial*"[tiab] OR "controlled trial*"[tiab] OR "Random Allocation"[Mesh] OR "Single-Blind Method"[Mesh] OR "Double-Blind Method"[Mesh] OR "Cross-Over Studies"[Mesh] OR "Clinical Trial"[Publication Type] OR "Clinical Trials as Topic"[Mesh] OR "Controlled Clinical Trial"[Publication Type] OR "Clinical Trial, Phase I"[Publication Type] OR "Clinical Trial, Phase II"[Publication Type] OR "Clinical Trial, Phase III"[Publication Type] OR "Clinical Trial, Phase IV"[Publication Type] OR "Non-Randomized Controlled Trials as Topic"[Mesh]	2,993,526
	#4	#1 AND #2 AND #3	409
Cochrane Library	#1	TITLE-ABS-KEY(Prostatic OR prostate OR "prostate neoplasm*" OR "prostatic neoplasm*" OR "prostate cancer*" OR "prostatic cancer*" OR "cancer of prostate" OR "neoplasm of prostate" OR "cancer of the prostate" OR "neoplasm of the prostate" OR "adenocarcinoma of prostate" OR "adenocarcinoma of the prostate" OR "squamous cell carcinoma of prostate" OR "squamous cell carcinoma of the prostate" OR "transitional cell carcinoma of prostate" OR "transitional cell carcinoma of the prostate" OR "cancers of prostate" OR "neoplasms of prostate" OR "cancers of the prostate" OR "neoplasms of the prostate" OR "adenocarcinomas of prostate" OR "adenocarcinomas of the prostate" OR "squamous cell carcinomas of prostate" OR "squamous cell carcinomas of the prostate" OR "transitional cell carcinomas of prostate" OR "transitional cell carcinomas of the prostate" OR "Castration-Resistant Prostate Neoplasm*" OR "Castration Resistant Prostate Neoplasm*" OR "Castration-Resistant Prostate Cancer*" OR "Castration Resistant Prostate cancer*" OR "Androgen-Resistant Prostate Neoplasm*" OR "Androgen Resistant Prostate Neoplasm*" OR "Androgen-Resistant Prostate Cancer*" OR "Androgen Resistant Prostate cancer*" OR "Androgen-Insensitive Prostate Neoplasm*" OR "Androgen Insensitive Prostate Neoplasm*" OR "Androgen-Insensitive Prostate Cancer*" OR "Androgen Insensitive Prostate cancer*" OR "Androgen-Independent Prostate Neoplasm*" OR "Androgen Independent Prostate Neoplasm*" OR "Androgen-Independent Prostate Cancer*" OR "Androgen Independent Prostate cancer*" OR "Hormone refractory Prostate Neoplasm*" OR "Hormone refractory Prostate Neoplasm*" OR "Hormone refractory Prostate Cancer*" OR "Hormone refractory Prostate cancer*" OR "Castration-Resistant Prostatic Neoplasm*" OR "Castration Resistant Prostatic Neoplasm*" OR "Castration-Resistant Prostatic Cancer*" OR "Castration Resistant Prostatic cancer*" OR "Androgen-Resistant Prostatic Neoplasm*" OR "Androgen Resistant Prostatic Neoplasm*" OR "Androgen-Resistant Prostatic Cancer*" OR "Androgen Resistant Prostatic cancer*" OR "Androgen-Insensitive Prostatic Neoplasm*" OR "Androgen Insensitive Prostatic Neoplasm*" OR "Androgen-Insensitive Prostatic Cancer*" OR "Androgen Insensitive Prostatic cancer*" OR "Androgen-Independent Prostatic Neoplasm*" OR "Androgen Independent Prostatic Neoplasm*" OR "Androgen-Independent Prostatic Cancer*" OR "Androgen Independent Prostatic cancer*" OR "Hormone refractory Prostatic Neoplasm*"	340,210

		OR “Hormone refractory Prostatic Neoplasm*” OR “Hormone refractory Prostatic Cancer*” OR “Hormone refractory Prostatic cancer*”)	
	#2	TITLE-ABS-KEY(“Immune Checkpoint Inhibitor*” OR “Immune Checkpoint Blocker*” OR “Immune Checkpoint Blockade*” OR “Immune Checkpoint Inhibition*” OR “PD-L1 Inhibitor*” OR “PD L1 Inhibitor*” OR “PDL1 Inhibitor*” OR “PD-L1 blocker*” OR “PD L1 blocker*” OR “PDL1 blocker*” OR “Programmed Death-Ligand 1 Inhibitor*” OR “Programmed Death Ligand 1 Inhibitor*” OR “Programmed Death-Ligand 1 blocker*” OR “Programmed Death Ligand 1 blocker*” OR “anti Programmed Death-Ligand 1” OR “anti-Programmed Death-Ligand 1” OR “anti Programmed Death Ligand 1” OR “anti-Programmed Death Ligand 1” OR “CTLA-4 Inhibitor*” OR “CTLA 4 Inhibitor*” OR “CTLA4 Inhibitor*” OR “CTLA-4 blocker*” OR “CTLA 4 blocker*” OR “CTLA4 blocker*” OR “Cytotoxic T-Lymphocyte Associated Protein 4 Inhibitor*” OR “Cytotoxic T Lymphocyte-Associated Protein 4 Inhibitor*” OR “Cytotoxic T-Lymphocyte Associated Protein 4 blocker*” OR “Cytotoxic T Lymphocyte-Associated Protein 4 blocker*” OR “Cytotoxic T-Lymphocyte Associated antigen 4 Inhibitor*” OR “Cytotoxic T Lymphocyte-Associated antigen 4 Inhibitor*” OR “Cytotoxic T-Lymphocyte Associated antigen 4 blocker*” OR “Cytotoxic T Lymphocyte-Associated antigen 4 blocker*” OR “Cytotoxic T-Lymphocyte-Associated Protein 4 Inhibitor*” OR “Cytotoxic T Lymphocyte Associated Protein 4 Inhibitor*” OR “Cytotoxic T-Lymphocyte-Associated Protein 4 blocker*” OR “Cytotoxic T Lymphocyte Associated Protein 4 blocker*” OR “Cytotoxic T-Lymphocyte-Associated antigen 4 Inhibitor*” OR “Cytotoxic T Lymphocyte Associated antigen 4 Inhibitor*” OR “Cytotoxic T-Lymphocyte-Associated antigen 4 blocker*” OR “Cytotoxic T Lymphocyte Associated antigen 4 blocker*” OR “anti-cytotoxic T lymphocyte-associated antigen 4” OR “anti cytotoxic T lymphocyte-associated antigen 4” OR “anti-cytotoxic T lymphocyte-associated protein 4” OR “anti cytotoxic T lymphocyte-associated protein 4” OR “anti-cytotoxic T-lymphocyte-associated antigen 4” OR “anti-cytotoxic T-lymphocyte-associated antigen 4” OR “PD-1 Inhibitor*” OR “PD 1 Inhibitor*” OR “PD1 Inhibitor*” OR “PD 1 blocker*” OR “PD1 blocker*” OR “PD-1 blocker*” OR “Programmed Cell Death Protein 1 Inhibitor*” OR “Programmed Cell Death Protein 1 blocker*” OR “anti Programmed Cell Death Protein 1” OR “anti-Programmed Cell Death Protein 1” OR “anti-PD1” OR “anti PD1” OR “anti-PD-1” OR “anti PD-1” OR “anti-PD-L1” OR “anti PDL1” OR “anti PD-L1” OR “anti-PDL1” OR “anti-PD L1” OR “anti PD L1” OR “anti-CTLA4” OR “anti-CTLA 4” OR “anti-CTLA-4” OR “anti CTLA4” OR “anti CTLA 4” OR “anti CTLA-4” OR “PD-1-PD-L1 Blockade*” OR “PD 1 PD L1 Blockade*” OR “Pembrolizumab” OR “MK-3475” OR “lambrolizumab” OR “Keytruda” OR “SCH-900475” OR “Nivolumab” OR “Opdivo” OR “ONO-4538” OR “ONO 4538” OR “ONO4538” OR “MDX-1106” OR “MDX 1106” OR “MDX1106” OR “BMS-936558” OR “BMS 936558” OR “BMS936558” OR “Ipilimumab” OR “Yervoy” OR “MDX 010” OR “MDX010” OR “MDX-010” OR “MDX-CTLA-4” OR “MDX CTLA 4” OR “Durvalumab” OR “MEDI4736” OR “MEDI-4736” OR “Imfinzi” OR “Dostarlimab” OR “GSK4057190” OR “TSR-042” OR “Cemiplimab” OR ”REGN2810” OR “Avelumab” OR “MSB-0010682” OR “MSB0010682” OR ”bavencio” OR ”MSB0010718C” OR ”MSB-0010718C” OR “Atezolizumab” OR “MPDL3280A” OR ”MPDL-3280A” OR “Tecentriq” OR “RG7446” OR “RG-7446” OR “pidilizumab” OR “CT-011” OR “CT 011” OR “tremelimumab” OR “ticilimumab” OR “CP 675” OR “CP675 cpd” OR “CP-675” OR “CP-	59,780

		675,206" OR "CP-675206" OR "CP675206" OR "CP 675206" OR "sintilimab" OR "SHR-1210" OR "camrelizumab" OR "toripalimab" OR "HX008" OR "tremelimumab")	
	#3	TITLE-ABS-KEY("RCT" OR "trial*" OR "intervention*" OR "placebo" OR "randomised trial*" OR "randomized trial*" OR "controlled trial*" OR "Random Allocation" OR "Single-Blind" OR "Double-Blind" OR "Cross-Over" OR "Clinical Trial*" OR "Controlled Clinical Trial" OR "Non-Randomized Controlled Trial*")	4,358,287
	#4	#1 AND #2 AND #3	1,865
Web of Science	#1	TS=(Prostatic OR prostate OR "prostate neoplasm*" OR "prostatic neoplasm*" OR "prostate cancer*" OR "prostatic cancer*" OR "cancer of prostate" OR "neoplasm of prostate" OR "cancer of the prostate" OR "neoplasm of the prostate" OR "adenocarcinoma of prostate" OR "adenocarcinoma of the prostate" OR "squamous cell carcinoma of prostate" OR "squamous cell carcinoma of the prostate" OR "transitional cell carcinoma of prostate" OR "transitional cell carcinoma of the prostate" OR "cancers of prostate" OR "neoplasms of prostate" OR "cancers of the prostate" OR "neoplasms of the prostate" OR "adenocarcinomas of prostate" OR "adenocarcinomas of the prostate" OR "squamous cell carcinomas of prostate" OR "squamous cell carcinomas of the prostate" OR "transitional cell carcinomas of prostate" OR "transitional cell carcinomas of the prostate" OR "Castration-Resistant Prostate Neoplasm*" OR "Castration Resistant Prostate Neoplasm*" OR "Castration-Resistant Prostate Cancer*" OR "Castration Resistant Prostate cancer*" OR "Androgen-Resistant Prostate Neoplasm*" OR "Androgen Resistant Prostate Neoplasm*" OR "Androgen-Resistant Prostate Cancer*" OR "Androgen Resistant Prostate cancer*" OR "Androgen-Insensitive Prostate Neoplasm*" OR "Androgen Insensitive Prostate Neoplasm*" OR "Androgen-Insensitive Prostate Cancer*" OR "Androgen Insensitive Prostate cancer*" OR "Androgen-Independent Prostate Neoplasm*" OR "Androgen Independent Prostate Neoplasm*" OR "Androgen-Independent Prostate Cancer*" OR "Androgen Independent Prostate cancer*" OR "Hormone refractory Prostate Neoplasm*" OR "Hormone refractory Prostate Neoplasm*" OR "Hormone refractory Prostate Cancer*" OR "Hormone refractory Prostate cancer*" OR "Castration-Resistant Prostatic Neoplasm*" OR "Castration Resistant Prostatic Neoplasm*" OR "Castration-Resistant Prostatic Cancer*" OR "Castration Resistant Prostatic cancer*" OR "Androgen-Resistant Prostatic Neoplasm*" OR "Androgen Resistant Prostatic Neoplasm*" OR "Androgen-Resistant Prostatic Cancer*" OR "Androgen Resistant Prostatic cancer*" OR "Androgen-Insensitive Prostatic Neoplasm*" OR "Androgen Insensitive Prostatic Neoplasm*" OR "Androgen-Insensitive Prostatic Cancer*" OR "Androgen Insensitive Prostatic cancer*" OR "Androgen-Independent Prostatic Neoplasm*" OR "Androgen Independent Prostatic Neoplasm*" OR "Androgen-Independent Prostatic Cancer*" OR "Androgen Independent Prostatic cancer*" OR "Hormone refractory Prostatic Neoplasm*" OR "Hormone refractory Prostatic Neoplasm*" OR "Hormone refractory Prostatic Cancer*" OR "Hormone refractory Prostatic cancer*")	336,544
	#2	TS=("Immune Checkpoint Inhibitor*" OR "Immune Checkpoint Blocker*" OR "Immune Checkpoint Blockade*" OR "Immune Checkpoint Inhibition*" OR "PD-L1 Inhibitor*" OR "PD L1 Inhibitor*" OR "PDL1 Inhibitor*" OR "PD-L1 blocker*" OR "PD L1 blocker*" OR "PDL1 blocker*" OR "Programmed Death-Ligand 1 Inhibitor*" OR "Programmed Death Ligand 1 Inhibitor*" OR "Programmed Death-Ligand 1 blocker*" OR "Programmed Death Ligand 1 blocker*" OR "anti Programmed Death-Ligand 1" OR "anti-Programmed Death-Ligand 1" OR "anti Programmed Death Ligand 1" OR "anti-Programmed Death Ligand 1" OR "CTLA-4	60,063

		Inhibitor*" OR "CTLA 4 Inhibitor*" OR "CTLA4 Inhibitor*" OR "CTLA-4 blocker*" OR "CTLA 4 blocker*" OR "CTLA4 blocker*" OR "Cytotoxic T-Lymphocyte Associated Protein 4 Inhibitor*" OR "Cytotoxic T Lymphocyte-Associated Protein 4 Inhibitor*" OR "Cytotoxic T-Lymphocyte Associated Protein 4 blocker*" OR "Cytotoxic T Lymphocyte-Associated Protein 4 blocker*" OR "Cytotoxic T-Lymphocyte Associated antigen 4 Inhibitor*" OR "Cytotoxic T Lymphocyte-Associated antigen 4 Inhibitor*" OR "Cytotoxic T-Lymphocyte Associated antigen 4 blocker*" OR "Cytotoxic T Lymphocyte-Associated antigen 4 blocker*" OR "Cytotoxic T-Lymphocyte-Associated Protein 4 Inhibitor*" OR "Cytotoxic T Lymphocyte Associated Protein 4 Inhibitor*" OR "Cytotoxic T-Lymphocyte-Associated Protein 4 blocker*" OR "Cytotoxic T Lymphocyte Associated Protein 4 blocker*" OR "Cytotoxic T-Lymphocyte-Associated antigen 4 Inhibitor*" OR "Cytotoxic T Lymphocyte Associated antigen 4 Inhibitor*" OR "Cytotoxic T-Lymphocyte-Associated antigen 4 blocker*" OR "Cytotoxic T Lymphocyte Associated antigen 4 blocker*" OR "anti-cytotoxic T lymphocyte-associated antigen 4" OR "anti cytotoxic T lymphocyte-associated antigen 4" OR "anti-cytotoxic T lymphocyte-associated protein 4" OR "anti cytotoxic T lymphocyte-associated protein 4" OR "anti-cytotoxic T-lymphocyte-associated antigen 4" OR "anti-cytotoxic T-lymphocyte-associated antigen 4" OR "PD-1 Inhibitor*" OR "PD 1 Inhibitor*" OR "PD1 Inhibitor*" OR "PD 1 blocker*" OR "PD1 blocker*" OR "PD-1 blocker*" OR "Programmed Cell Death Protein 1 Inhibitor*" OR "Programmed Cell Death Protein 1 blocker*" OR "anti Programmed Cell Death Protein 1" OR "anti-Programmed Cell Death Protein 1" OR "anti-PD1" OR "anti PD1" OR "anti-PD-1" OR "anti PD-1" OR "anti-PD-L1" OR "anti PDL1" OR "anti PD-L1" OR "anti-PDL1" OR "anti-PD L1" OR "anti PD L1" OR "anti-CTLA4" OR "anti-CTLA 4" OR "anti-CTLA-4" OR "anti CTLA4" OR "anti CTLA 4" OR "anti CTLA-4" OR "PD-1-PD-L1 Blockade*" OR "PD 1 PD L1 Blockade*" OR "Pembrolizumab" OR "MK-3475" OR "lambrolizumab" OR "Keytruda" OR "SCH-900475" OR "Nivolumab" OR "Opdivo" OR "ONO-4538" OR "ONO 4538" OR "ONO4538" OR "MDX-1106" OR "MDX 1106" OR "MDX1106" OR "BMS-936558" OR "BMS 936558" OR "BMS936558" OR "Ipilimumab" OR "Yervoy" OR "MDX 010" OR "MDX010" OR "MDX-010" OR "MDX-CTLA-4" OR "MDX CTLA 4" OR "Durvalumab" OR "MEDI4736" OR "MEDI-4736" OR "Imfinzi" OR "Dostarlimab" OR "GSK4057190" OR "TSR-042" OR "Cemiplimab" OR "REGN2810" OR "Avelumab" OR "MSB-0010682" OR "MSB0010682" OR "bavencio" OR "MSB0010718C" OR "MSB-0010718C" OR "Atezolizumab" OR "MPDL3280A" OR "MPDL-3280A" OR "Tecentriq" OR "RG7446" OR "RG-7446" OR "pidilizumab" OR "CT-011" OR "CT 011" OR "tremelimumab" OR "ticilimumab" OR "CP 675" OR "CP675 cpd" OR "CP-675" OR "CP-675,206" OR "CP-675206" OR "CP675206" OR "CP 675206" OR "sintilimab" OR "SHR-1210" OR "camrelizumab" OR "toripalimab" OR "HX008" OR "tremelimumab")	
	#3	TS=("RCT" OR "trial*" OR "intervention*" OR "placebo" OR "randomised trial*" OR "randomized trial*" OR "controlled trial*" OR "Random Allocation" OR "Single-Blind" OR "Double-Blind" OR "Cross-Over" OR "Clinical Trial*" OR "Controlled Clinical Trial" OR "Non-Randomized Controlled Trial*")	3,283,784
	#4	#1 AND #2 AND #3	797
Embase	#1	(Prostatic OR prostate OR "prostate neoplasm*" OR "prostatic neoplasm*" OR "prostate cancer*" OR "prostatic cancer*" OR "cancer of prostate" OR "neoplasm of prostate" OR "cancer of the prostate" OR "neoplasm of the prostate" OR "adenocarcinoma of prostate" OR	342,405

		“adenocarcinoma of the prostate” OR “squamous cell carcinoma of prostate” OR “squamous cell carcinoma of the prostate” OR “transitional cell carcinoma of prostate” OR “transitional cell carcinoma of the prostate” OR “cancers of prostate” OR “neoplasms of prostate” OR “cancers of the prostate” OR “neoplasms of the prostate” OR “adenocarcinomas of prostate” OR “adenocarcinomas of the prostate” OR “squamous cell carcinomas of prostate” OR “squamous cell carcinomas of the prostate” OR “transitional cell carcinomas of prostate” OR “transitional cell carcinomas of the prostate” OR “Castration-Resistant Prostate Neoplasm*” OR “Castration Resistant Prostate Neoplasm*” OR “Castration-Resistant Prostate Cancer*” OR “Castration Resistant Prostate cancer*” OR “Androgen-Resistant Prostate Neoplasm*” OR “Androgen Resistant Prostate Neoplasm*” OR “Androgen-Resistant Prostate Cancer*” OR “Androgen Resistant Prostate cancer*” OR “Androgen-Insensitive Prostate Neoplasm*” OR “Androgen Insensitive Prostate Neoplasm*” OR “Androgen-Insensitive Prostate Cancer*” OR “Androgen Insensitive Prostate cancer*” OR “Androgen-Independent Prostate Neoplasm*” OR “Androgen Independent Prostate Neoplasm*” OR “Androgen-Independent Prostate Cancer*” OR “Androgen Independent Prostate cancer*” OR “Hormone refractory Prostate Neoplasm*” OR “Hormone refractory Prostate Neoplasm*” OR “Hormone refractory Prostate Cancer*” OR “Hormone refractory Prostate cancer*” OR “Castration-Resistant Prostatic Neoplasm*” OR “Castration Resistant Prostatic Neoplasm*” OR “Castration-Resistant Prostatic Cancer*” OR “Castration Resistant Prostatic cancer*” OR “Androgen-Resistant Prostatic Neoplasm*” OR “Androgen Resistant Prostatic Neoplasm*” OR “Androgen-Resistant Prostatic Cancer*” OR “Androgen Resistant Prostatic cancer*” OR “Androgen-Insensitive Prostatic Neoplasm*” OR “Androgen Insensitive Prostatic Neoplasm*” OR “Androgen-Insensitive Prostatic Cancer*” OR “Androgen Insensitive Prostatic cancer*” OR “Androgen-Independent Prostatic Neoplasm*” OR “Androgen Independent Prostatic Neoplasm*” OR “Androgen-Independent Prostatic Cancer*” OR “Androgen Independent Prostatic cancer*” OR “Hormone refractory Prostatic Neoplasm*” OR “Hormone refractory Prostatic Neoplasm*” OR “Hormone refractory Prostatic Cancer*” OR “Hormone refractory Prostatic cancer*”):ab,ti	
	#2	(“Immune Checkpoint Inhibitor*” OR “Immune Checkpoint Blocker*” OR “Immune Checkpoint Blockade*” OR “Immune Checkpoint Inhibition*” OR “PD-L1 Inhibitor*” OR “PD L1 Inhibitor*” OR “PDL1 Inhibitor*” OR “PD-L1 blocker*” OR “PD L1 blocker*” OR “PDL1 blocker*” OR “Programmed Death-Ligand 1 Inhibitor*” OR “Programmed Death Ligand 1 Inhibitor*” OR “Programmed Death-Ligand 1 blocker*” OR “Programmed Death Ligand 1 blocker*” OR “anti Programmed Death-Ligand 1” OR “anti-Programmed Death-Ligand 1” OR “anti Programmed Death Ligand 1” OR “anti-Programmed Death Ligand 1” OR “CTLA-4 Inhibitor*” OR “CTLA 4 Inhibitor*” OR “CTLA4 Inhibitor*” OR “CTLA-4 blocker*” OR “CTLA 4 blocker*” OR “CTLA4 blocker*” OR “Cytotoxic T-Lymphocyte Associated Protein 4 Inhibitor*” OR “Cytotoxic T Lymphocyte-Associated Protein 4 Inhibitor*” OR “Cytotoxic T-Lymphocyte Associated Protein 4 blocker*” OR “Cytotoxic T Lymphocyte-Associated Protein 4 blocker*” OR “Cytotoxic T-Lymphocyte Associated antigen 4 Inhibitor*” OR “Cytotoxic T Lymphocyte-Associated antigen 4 Inhibitor*” OR “Cytotoxic T-Lymphocyte Associated antigen 4 blocker*” OR “Cytotoxic T Lymphocyte-Associated antigen 4 blocker*” OR “Cytotoxic T-Lymphocyte-Associated Protein 4 Inhibitor*” OR “Cytotoxic T Lymphocyte Associated Protein 4 Inhibitor*” OR “Cytotoxic T-Lymphocyte-Associated Protein 4 blocker*” OR “Cytotoxic T Lymphocyte Associated Protein 4 blocker*” OR “Cytotoxic T-Lymphocyte-Associated antigen 4	74,501

		Inhibitor*" OR "Cytotoxic T Lymphocyte Associated antigen 4 Inhibitor*" OR "Cytotoxic T-Lymphocyte-Associated antigen 4 blocker*" OR "Cytotoxic T Lymphocyte Associated antigen 4 blocker*" OR "anti-cytotoxic T lymphocyte-associated antigen 4" OR "anti cytotoxic T lymphocyte-associated antigen 4" OR "anti-cytotoxic T lymphocyte-associated protein 4" OR "anti cytotoxic T lymphocyte-associated protein 4" OR "anti-cytotoxic T-lymphocyte-associated antigen 4" OR "anti-cytotoxic T-lymphocyte-associated antigen 4" OR "PD-1 Inhibitor*" OR "PD 1 Inhibitor*" OR "PD1 Inhibitor*" OR "PD 1 blocker*" OR "PD1 blocker*" OR "PD-1 blocker*" OR "Programmed Cell Death Protein 1 Inhibitor*" OR "Programmed Cell Death Protein 1 blocker*" OR "anti Programmed Cell Death Protein 1" OR "anti-Programmed Cell Death Protein 1" OR "anti-PD1" OR "anti PD1" OR "anti-PD-1" OR "anti PD-1" OR "anti-PD-L1" OR "anti PDL1" OR "anti PD-L1" OR "anti-PDL1" OR "anti-PD L1" OR "anti PD L1" OR "anti-CTLA4" OR "anti-CTLA 4" OR "anti-CTLA-4" OR "anti CTLA4" OR "anti CTLA 4" OR "anti CTLA-4" OR "PD-1-PD-L1 Blockade*" OR "PD 1 PD L1 Blockade*" OR "Pembrolizumab" OR "MK-3475" OR "lambrolizumab" OR "Keytruda" OR "SCH-900475" OR "Nivolumab" OR "Opdivo" OR "ONO-4538" OR "ONO 4538" OR "ONO4538" OR "MDX-1106" OR "MDX 1106" OR "MDX1106" OR "BMS-936558" OR "BMS 936558" OR "BMS936558" OR "Ipilimumab" OR "Yervoy" OR "MDX 010" OR "MDX010" OR "MDX-010" OR "MDX-CTLA-4" OR "MDX CTLA 4" OR "Durvalumab" OR "MEDI4736" OR "MEDI-4736" OR "Imfinzi" OR "Dostarlimab" OR "GSK4057190" OR "TSR-042" OR "Cemiplimab" OR "REGN2810" OR "Avelumab" OR "MSB-0010682" OR "MSB0010682" OR "bavencio" OR "MSB0010718C" OR "MSB-0010718C" OR "Atezolizumab" OR "MPDL3280A" OR "MPDL-3280A" OR "Tecentriq" OR "RG7446" OR "RG-7446" OR "pidilizumab" OR "CT-011" OR "CT 011" OR "tremelimumab" OR "ticilimumab" OR "CP 675" OR "CP675 cpd" OR "CP-675" OR "CP-675,206" OR "CP-675206" OR "CP675206" OR "CP 675206" OR "sintilimab" OR "SHR-1210" OR "camrelizumab" OR "toripalimab" OR "HX008" OR "tremelimumab"):ab,ti	
	#3	("RCT" OR "trial*" OR "intervention*" OR "placebo" OR "randomised trial*" OR "randomized trial*" OR "controlled trial*" OR "Random Allocation" OR "Single-Blind" OR "Double-Blind" OR "Cross-Over" OR "Clinical Trial*" OR "Controlled Clinical Trial" OR "Non-Randomized Controlled Trial*"):ab,ti	3,356,739
	#4	#1 AND #2 AND #3	818
